# Design, Synthesis, and Evaluation of New Hybrid Derivatives of 5,6-Dihydro-4*H*-pyrrolo[3,2,1-*ij*]quinolin-2(1*H*)-one as Potential Dual Inhibitors of Blood Coagulation Factors Xa and XIa

**DOI:** 10.3390/molecules29020373

**Published:** 2024-01-11

**Authors:** Anna A. Skoptsova, Athina Geronikaki, Nadezhda P. Novichikhina, Alexey V. Sulimov, Ivan S. Ilin, Vladimir B. Sulimov, Georgii A. Bykov, Nadezhda A. Podoplelova, Oleg V. Pyankov, Khidmet S. Shikhaliev

**Affiliations:** 1 Department of Organic Chemistry, Faculty of Chemistry, Voronezh State University, 1 Universitetskaya Sq., 394018 Voronezh, Russia; annk0611@mail.ru (A.A.S.); novichikhina@chem.vsu.ru (N.P.N.); 2 School of Pharmacy, Aristotle University of Thessaloniki, 54124 Thessaloniki, Greece; 3 Research Computing Center, Lomonosov Moscow State University, 119992 Moscow, Russia; sulimovv@mail.ru (A.V.S.); ivan.ilyin@srcc.msu.ru (I.S.I.); vladimir.sulimov@gmail.com (V.B.S.); 4 Department of Biophysics at the Faculty of Physics, Lomonosov Moscow State University, 119992 Moscow, Russia; bga20000@gmail.com; 5 Center for Theoretical Problems of Physicochemical Pharmakology, 119991 Moscow, Russia; podoplelova.nadezhda@ctppcp.ru; 6 State Research Center of Virology and Biotechnology “Vector”, 630559 Koltsovo, Russia; pyankov_ov@vector.nsc.ru

**Keywords:** 5,6-dihydro-4*H*-pyrrolo[3,2,1-*ij*]quinolin-2-one, thiazole, anticoagulant activity, dual activity, inhibitor, factor Xa, factor XIa, thrombin, blood coagulation, molecular docking

## Abstract

Cardiovascular diseases caused by blood coagulation system disorders are one of the leading causes of morbidity and mortality in the world. Research shows that blood clotting factors are involved in these thrombotic processes. Among them, factor Xa occupies a key position in the blood coagulation cascade. Another coagulation factor, XIa, is also a promising target because its inhibition can suppress thrombosis with a limited contribution to normal hemostasis. In this regard, the development of dual inhibitors as new generation anticoagulants is an urgent problem. Here we report the synthesis and evaluation of novel potential dual inhibitors of coagulation factors Xa and XIa. Based on the principles of molecular design, we selected a series of compounds that combine in their structure fragments of pyrrolo[3,2,1-*ij*]quinolin-2-one and thiazole, connected through a hydrazine linker. The production of new hybrid molecules was carried out using a two-stage method. The reaction of 5,6-dihydropyrrolo[3,2,1-*ij*]quinoline-1,2-diones with thiosemicarbazide gave the corresponding hydrazinocarbothioamides. The reaction of the latter with DMAD led to the target methyl 2-(4-oxo-2-(2-(2-oxo-5,6-dihydro-*4H*-pyrrolo[3,2,1-*ij*]quinolin-1(*2H*)-ylidene)hydrazineyl)thiazol-5(*4H*)-ylidene)acetates in high yields. In vitro testing of the synthesized molecules revealed that ten of them showed high inhibition values for both the coagulation factors Xa and XIa, and the IC_50_ value for some compounds was also assessed. The resulting structures were also tested for their ability to inhibit thrombin.

## 1. Introduction

Diseases resulting from disorders of the blood coagulation system continue to be the leading cause of morbidity and mortality in the modern world [1]. Antithrombotic therapy is based on the use of anticoagulants—substances that directly inhibit the coagulation factors or disrupt their formation in the liver (vitamin K antagonists). In recent years, for the prevention of thromboembolic complications during general surgical and orthopedic interventions in operative gynecology and transplantology, preference is given to direct oral anticoagulants, since they have a number of advantages compared to vitamin K antagonists [2]. Over the past few years, this problem has become even more acute. Thromboembolic disorders have become one of the most serious clinical manifestations of COVID-19, leading to complications of the disease, as well as increased mortality [3,4]. Moreover, the risk of blood clots remains high months after the illness. Therefore, the prevention and treatment of thrombosis is currently one of the main tasks of medical practice.

Modern anticoagulant therapy has its limitations and disadvantages, which can lead to serious side effects, as well as increased mortality. Such disadvantages include, for example, the risk of bleeding [2]. The presence of imperfections in existing therapy has led to the search for new promising targets and the emergence of new approaches to the development of highly effective and non-toxic anticoagulant drugs. One of these developing areas is the development and use of small-molecule inhibitors of serine proteases [5].

Recent studies show that targeting multiple targets involved in blood coagulation has advantages over the use of selective inhibitors of individual targets [6,7,8,9,10]. In the development of dual inhibitors, research has focused mainly on the key enzymes in the coagulation cascade—thrombin and factor Xa [7,11,12,13,14,15].

Thrombin is the most obvious target for the development of anticoagulant inhibitors. However, studies show that it is thrombin inhibition that inevitably increases the likelihood of hemorrhagic complications, especially when used in conjunction with thrombolytic drugs [16,17,18]. Factor Xa, in turn, remains an extremely attractive target protein, inhibitors of which are gradually being included in clinical practice as agents for the treatment of various types of thrombosis [2]. However, the question of developing factor Xa inhibitors that do not bind to thrombin remains open, due to the structural similarity of both enzymes.

At the same time, in recent years there has been increasing interest by the scientific community in the internal pathway of blood coagulation, showing the importance of its contribution to thrombosis [19,20,21]. Increasing evidence suggests that factor XIa, which belongs to the intrinsic coagulation activation pathway, is an attractive target [21,22,23,24]. FXIa is a long-lived factor, which can circulate in the bloodstream for a long time and plays an important role in thrombosis. However, the data show that factor XIa has a limited effect on normal hemostasis [21,25]. This is a necessary condition in the development of new generation anticoagulant drugs with a reduced risk of internal bleeding.

Thus, obtaining effective and, at the same time, safe anticoagulant drugs for the prevention and treatment of thrombosis may become possible through the development of dual inhibitors of blood coagulation factors Xa and XIa, which at the same time have a limited effect on thrombin.

Thiazole derivatives have attracted the attention of the scientific community due to their wide spectrum of biological activities, such as antimicrobial [26,27], anti-inflammatory [28,29], anticancer [30,31], anti-HIV [32], antidiabetic [33], carbonic anhydrase inhibitory [34,35], antitubercular [36], anticoagulant [37,38], and many other [39,40] activities.

Another interesting core is quinoline, derivatives which are known also to perform a wide range of biological activities. They are reported to possess anticoagulant [41], antibacterial [42], anti-Alzheimer [43], anticancer [44], antidiabetic [45] properties and others [46,47,48]. On the other hand, pyrrole derivatives possess anticoagulant [49], antimicrobial [50], anti-inflammatory [51,52] and anticancer [53], anti-HIV [54] effects.

Prompted by all the above-mentioned, and our previous studies [55,56,57,58,59,60], we designed and synthesized, based on the hybridization approach, new methyl 2-(4-oxo-2-(2-(2-oxo-5,6-dihydro-4*H*-pyrrolo[3,2,1-*ij*]quinolin-1(2*H*)-ylidene)hydrazineyl)thiazol-5(4H)-ylidene)acetates and studied their inhibitory properties against blood clotting factors Xa and XIa in vitro.

## 2. Results and Discussion

### 2.1. Design of 5,6-Dihydro-4H-pyrrolo[3,2,1-ij]quinolin-2(1H)-one-Based Derivatives

In the modern design of biologically active compounds with specified properties, one of the common approaches is to obtain hybrid molecules that carry in their structure fragments of known pharmacophores, as well as substructures whose ability to interact with specified targets has been studied. This approach makes it possible to create complex molecules that potentially have dual or multiple activities [61,62,63,64,65].

In a number of our previous publications aimed at the synthesis and study of the anticoagulant activity of functional derivatives of pyrrolo[3,2,1-*ij*]quinolin-2-ones, promising results were obtained and the promise of these structures in the synthesis of new inhibitors of factors Xa and XIa shown [55,56,57,58,59,60]. It has been shown that 5,6-dihydro-4*H*-pyrrolo[3,2,1-*ij*]quinolin-2-ones, and in particular 6-aryl-substituted ones, tend to exhibit higher activity compared to their unsaturated analogues [55,56,66].

Furthermore, we also demonstrated that molecules combining in their structure fragments of 5,6-dihydro-4*H*-pyrrolo[3,2,1-*ij*]quinolin-2-one and thiazole act as both selective and dual inhibitors of Xa factors and XIa [56,57]. High inhibition values for both factors were also found for a compound in which pyrrolo[3,2,1-*ij*]quinolin-2-one is linked to a thiazole derivative via a hydrazine linker [58].

Another possible modification of the starting 5,6-dihydro-4*H*-pyrrolo[3,2,1-*ij*]quinolin-2-ones to improve the characteristics of the final biologically active products is the introduction of substituents into the benzene ring of the quinoline ring, for example, halogen, and also their variation. A similar approach had previously been repeatedly implemented to increase the biological activity of compounds, change their physical and chemical properties, increase the selectivity of action, and also reduce side effects [47,48,56,66,67,68].

In the continuation of our studies on the search for compounds with dual inhibitory activity, in this work we also focused on combining the described fragments in the frame of a single molecule. Thus, methyl 2-(4-oxothiazol-5(4*H*)-ylidene)acetate, which is easy to prepare, was chosen as the thiazole-containing fragment. This heterocyclic fragment is a well-known pharmacophore among the functional derivatives of which compounds with antitumor [69,70,71,72], antimicrobial [65,73,74,75], antifungal [75], antiviral [76,77], antioxidant [65,78], anti-inflammatory [79], as well as anticoagulant [80] activity have been found.

Thus, herein we report the design and synthesis of new methyl 2-(4-oxo-2-(2-(2-oxo-5,6-dihydro-4*H*-pyrrolo[3,2,1-*ij*]quinolin-1(2*H*)-ylidene)hydrazineyl)thiazol-5(4*H*)-ylidene)acetates and study their inhibitory properties against blood clotting factors Xa and XIa in vitro.

Furthermore, computer docking was also carried out, which made it possible to evaluate the presented molecules as inhibitors by placing them in the active center of the target protein, with the subsequent calculation of interactions.

### 2.2. Synthesis

To obtain the target 1-(2-(4,5-dihydrothiazol-2-yl)hydrazinylidene)-5,6-dihydro-4*H*-pyrrolo[3,2,1-*ij*]quinolin-2-ones **3**, we used the following strategy. At the first stage, the corresponding hydrazinocarbothioamides **2a-q** were obtained by condensation of pyrrolo[3,2,1-*ij*]quinoline-1,2-diones **1a-q** with thiosemicarbazide. The reaction was carried out by analogy with [58,81], in which the reagents were boiled in isopropyl alcohol. However, in our case the best results were achieved by boiling the starting compounds in methanol with the addition of catalytic amounts of HCl. The reaction carried out using this method, according to an LCMS analysis of the reaction masses (shown using the example of compound **2b**, see Supplementary Materials, Figure S70), proceeded with high conversion, reaching more than 90%. It was found that isolation of the obtained thiosemicarbazones **2a-q** was not required for the second stage. The reaction mass was, if necessary, evaporated using a rotary evaporator, and the resulting residue containing intermediate compounds **2** was then introduced into the next stage of the reaction.

**Scheme 1:**
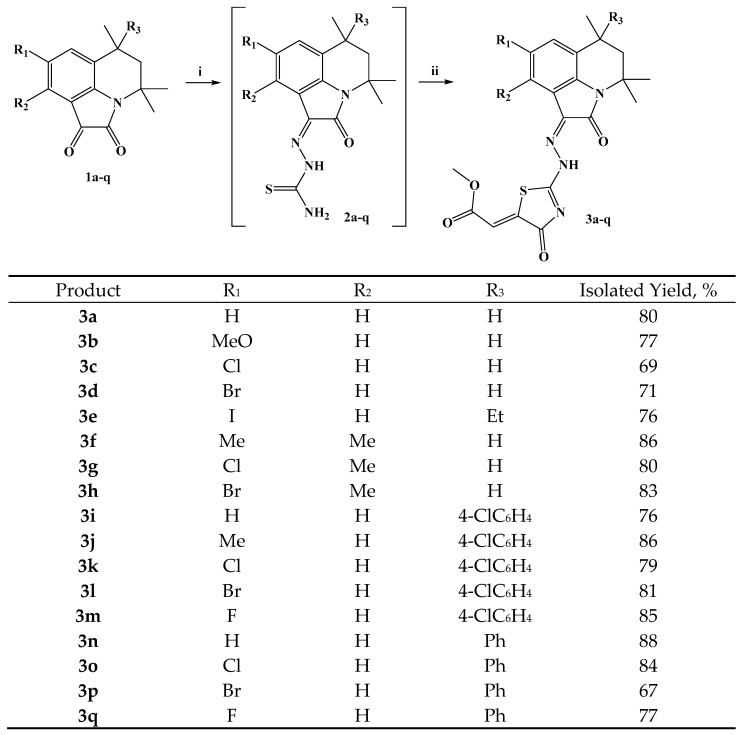
Preparation of 2-oxo-5,6-dihydropyrrolo[3,2,1-*ij*]quinolin-1-ylidene)hydrazineyl)-4-oxothiazol-5-ylidene)acetates **3a-q**. Reagents and conditions: (i) thiosemicarbazide, MeOH/HCl, reflux, 1 h; (ii) DMAD, MeOH/AcOH (*v*/*v* 4:1), reflux, 30–60 min.

To carry out the heterocyclization reaction of intermediate hydrazinocarbothioamides **2a-q** with commercially available dimethylacetylenedicarboxylate (DMAD), the conditions described in the literature for similar systems were used. By carrying out the reaction in methanol [70,71,73] or ethanol [65,82,83,84], it was not possible to achieve complete conversion of the reagents. Using the MeOH/AcOH system (*v*/*v* 4:1) as a medium led to a reduction in the reaction time and an increase in the yield of products **3a-q** to almost quantitative ones. Thus, the reaction of hydrazinocarbothioamides **2** with DMAD by boiling in methanol with the addition of acetic acid for 30–60 min made it possible to obtain a series of new methyl 2-oxo-5,6-dihydropyrrolo[3,2,1-*ij*]quinolin-1-ylidene)hydrazineyl)-4-oxothiazol-5-ylidene)acetates **3a-q** in high yields of 67–88% (Scheme 1).

The presence of a C=N double bond, as well as an exocyclic C=C double bond in the thiazole ring, leads to the possibility of the existence of structures **3** in the form of geometric isomers. Based on the data of HPLC MS analysis and ^1^H and ^13^C NMR spectroscopy, it was found that most of the compounds **3a-d**, **f-i**, **k-l**, **n-q** are a single isomer. According to the literature data [58,81], thiosemicarbazones of type **2**, as well as compounds derived from them, have predominantly a *Z*-configuration of the C=N double bond due to the possibility of forming an intramolecular hydrogen bond with the C=O group of pyrrolo[3,2,1-*ij*]quinolin-2-one fragment.

We determined the configuration of the exocyclic double bond C=C at the thiazole ring in compounds **3** to be a *Z*-isomer based on ^1^H NMR spectroscopy data. In the ^1^H NMR spectra of these structures, resonance of the proton of the methine fragment is observed, as expected, at lower chemical shift values (6.69–6.76 ppm) than would be the case for *E*-isomers [85,86]. The proposed *Z*-configuration is also consistent with the previously reported mechanism of trans-addition of the SH-group to the triple bond in DMAD in similar systems [87]. Thus, we assumed that compounds **3** exist predominantly in the form of the *Z,Z*-configuration.

The compounds **3e,j,m** were obtained and studied as a mixture of geometric isomers. Based on ^1^H NMR spectroscopy data, we suggest that compounds **3e,m** were isolated as a mixture of *Z*,*Z*- and *E*,*Z*-isomers, as evidenced by the duplication of the characteristic olefinic proton signals. In the case of compound **3j**, the duplication of the singlet signal of the olefin proton is not observed in the 1H NMR spectrum, in contrast to the duplication of the signals of the methylene and aromatic protons. This may indicate the existence of **3j** in the form of a mixture of *Z*,*Z*- and *Z*,*E*-isomers.

### 2.3. Anticoagulant Studies

All the synthesized compounds were primary in vitro screened toward factors Xa and XIa to determine their anticoagulant activity, and the data are presented in Table 1. As a reference compound, Rivaroxaban was used [88].

For compounds with high inhibition values of both factors **3b-c**, **e-f**, **k-l**, **n-p**, the kinetics of hydrolysis of specific substrates S2765 for factor Xa and S2366 for factor XIa in a buffer solution and in the presence of various concentrations of compounds were measured to determine the IC_50_ values (Table 1). The dependence of inhibition of Factor Xa- and XIa-induced chromogenic substrate hydrolysis on the concentration of compounds **3b-c**, **e-f**, **k-l**, **n-p** is presented in Supplementary Materials, Figures S52–S69.

Among the compounds studied, ten molecules exhibited high inhibition values for both blood clotting factors Xa and XIa **3c-f**, **k-l**, **n-q** (>75%). This fact shows the advantage of the above dual molecules compared to Rivaroxaban, which is only a factor Xa inhibitor. Along with this, two compounds showed high inhibition values selectively against FXa **3b,i** (87–91%), while compound **3j** showed the same against FXIa (83%). It is important to note that compounds **3i** and **3j** contain a 4-chlorophenyl moiety at position 6 of the tricyclic system.

Diversity in this line of compounds was achieved by varying the substituents in the 6, 8, 9 positions of the pyrrolo[3,2,1-*ij*]quinolinone fragment. It can be noted that FXa was more tolerant to various combinations of substituents than FXIa. In this case, an increase in the percentage of factor Xa inhibition by compounds containing an aryl substituent in the 6th position is clearly observed. On the other hand, the introduction of substituents, including halogen ones, into the 8th position of the pyrrolo[3,2,1-*ij*]quinolin-2-one fragment led to an increase in the inhibitory activity against FXIa.

The resulting dual inhibitors of blood clotting factors Xa and XIa can also affect thrombin, due to the structural similarity of the active centers of thrombin and factor Xa. As mentioned earlier, the inhibition of thrombin entails pathological processes associated with the disruption of normal hemostasis. This effect is undesirable when searching for new generation inhibitors.

Therefore, we conducted additional research. The results of the in vitro experiment, shown in Table 2, indicate a fairly low percentage of thrombin inhibition for most of the obtained compounds **3a-d, g-j, m-n, q**, which is maybe an indication of a potentially insignificant effect of these compounds on normal human hemostasis.

It was found, that only one compound **3e** with an iodine atom at position 8 of the tricyclic system is a multitarget inhibitor, acting equally strongly on both factors Xa and XIa and thrombin.

### 2.4. Identifying Compound-Protein Interactions

To determine the structural patterns important for binding to the target protein, the geometries of protein–ligand complexes predicted by docking for the identified factor Xa and factor XIa inhibitors were analyzed.

**Figure 1:**
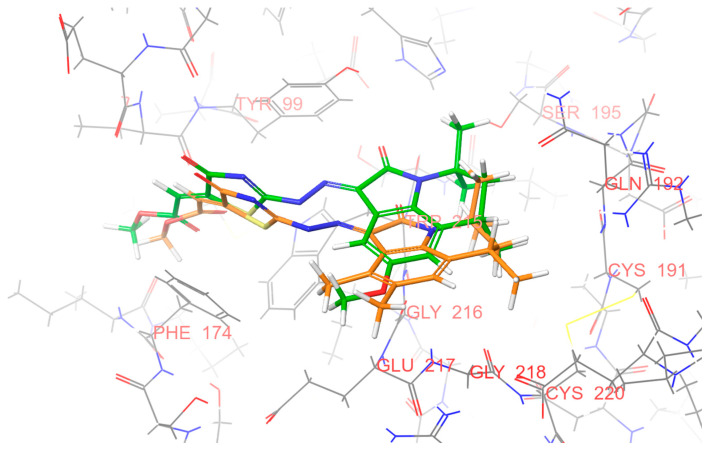
Docking pose of **3b** and **3f** in an active site of factor Xa.

The active site of factor Xa is represented by the S1 pocket and the S4 pocket, as well as the surrounding residues. The S1 pocket comprised Trp215-Gly216 on one side and Ala190-Cys191-Gln192 on the other. The lower part of the S1 pocket is similar to the S1 pocket of thrombin and is formed by two residues—Asp189 and Tyr228—as well as residues Cys191-Cys220. Therefore, engagement to this part of the target protein can lead to the inhibition of both thrombin and factor Xa. On the contrary, the S4 pocket is different from other serine proteases and is formed by the side chains of Tyr99, Phe174 and Trp215. Because of this, one of the possible approaches to obtaining factor Xa inhibitors selective over thrombin is specifically targeting the S4 pocket [67,89]. For the compounds we synthesized and studied, two distinct binding modes are predicted by docking. The first mode of interaction is observed for compounds **3a**-**h** and is shown in Figure 1, where **3b** and **3f** are depicted as examples. As can be seen from the figure, the central scaffold of **3b** and **3f** is positioned over Gly-216 with the amide oxygen of the pyrrole moiety directed toward Tyr-99. Such an orientation of the scaffold favors binding of the thiazolidine warhead inside the S4 pocket. The thiazolidine ring can potentially interact via π-stacking with the aromatic rings of Tyr-99 and Phe-174. The molecule **3b** is positioned slightly closer to Tyr-99 and can form a hydrogen bond with the phenolic hydroxyl of this residue. For both **3b** and **3f**, the S1 pocket is left unoccupied.

**Figure 2:**
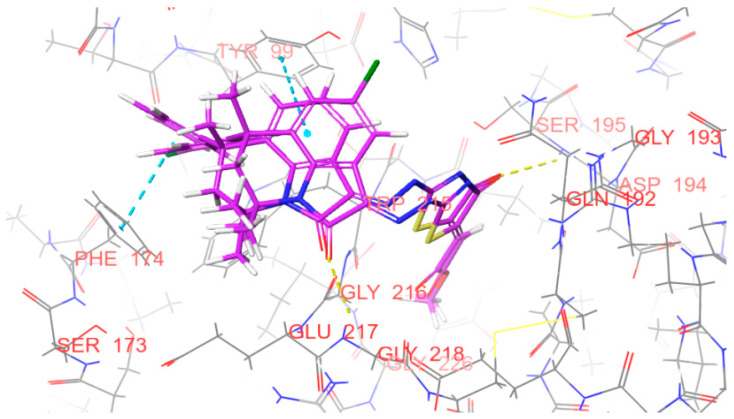
Docking poses of **3k** and **3n** in the active site of factor Xa. The blue dotted lines indicate π-stacking. Yellow dotted lines indicate hydrogen bonds.

Compounds **3i**-**q** that are substituted at C6 of the pyrroloquinolinone core with an aryl substituent show the second binding mode to factor Xa, depicted in Figure 2 with **3k** and **3n** used as examples. Compared to the first binding mode, where the amide oxygen is directed toward Tyr-99, in factor Xa complexes with **3k** and **3n** the scaffold is inverted in such a way that this oxygen points out to Gly-217. In the case of **3k**, this amide oxygen visually forms a hydrogen bond with Gly-217NH. The thiazolidine warhead **3k** and **3n** is positioned near the catalytic Ser-195. In this regard, the potential formation of a covalent bond between this residue and the reactive part of the warhead can be proposed. Also, the presence of an aryl fragment in structures **3k** and **3n** provides the opportunity to occupy the S4 pocket, in which π-stacking interactions with Tyr-99 and Phe-174 are detected.

It is noteworthy that in both the binding modes, the S1 pocket remains unoccupied, while the S4 pocket is occupied by a thiazole fragment in the first case and by an aryl substituent at the 6th position of the pyrrolo[3,2,1-*ij*]quinoline core in the second case.

The active site of factor XIa is also represented by several pockets. The S1 pocket is the only deep pocket in the binding interface. It is formed by β-strands ending in a disulfide bridge between Cys191-Cys219 and Asp189, which is located at its base. The S2 pocket is located adjacent to His57 and bounded by Tyr58B. The S1′ pocket is located opposite the catalytic triad His57b, Asp102, and Ser195, and next to another disulfide bridge formed by Cys40-Cys58. The S2′ pocket contains the β-strand which includes Arg39, His40, Leu41, Ile151, and Tyr143.

**Figure 3:**
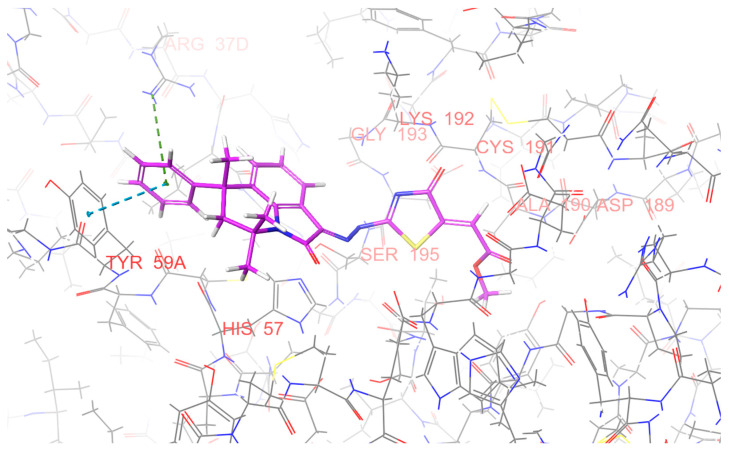
**3n** docked into an active site of FXIa. Green lines and blue lines indicate pi-cation and pi-pi-stacking interaction, respectively.

In the case of factor XIa, the compounds **3a**-**q** are located in the active site of the target protein in a similar way, irrespective of the substituent at C6. The generalized binding mode of structures **3i**-**q** in the active site of factor XIa is shown in Figure 3 with compound **3n** depicted as an example. A docking pose of **3n** reveals that this compound could occupy two of the three pockets of an active site of factor XIa. A thiazolone moiety linked to an ester group is extended into the S1 pocket blocking the catalytic triad of enzyme. At the other end of the molecule, a phenyl ring attached to a pyrrolo[3,2,1-*ij*]quinoline scaffold occupies the S2 pocket and forms two interactions: T-shaped pi-pi stacking with Tyr-59 and pi-cation interaction with Arg-37. The planar aromatic part of the scaffold is placed above His-57 and could potentially interact with His-57. The S2′ pocket is left unoccupied and in theory could be explored via adding ring substituents of the core at C8 or C9.

**Figure 4:**
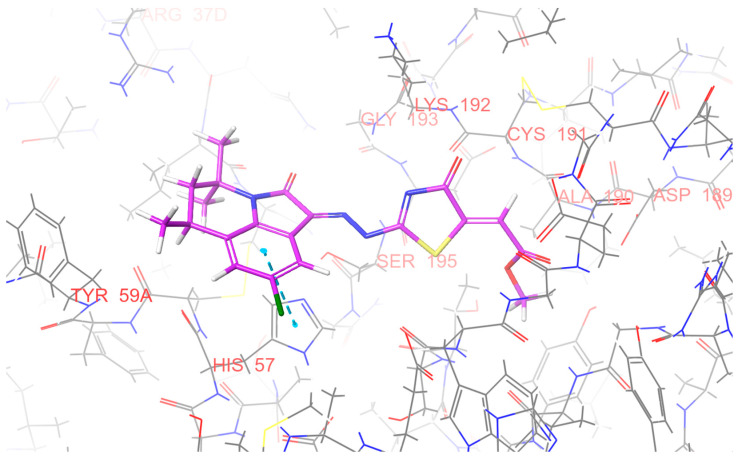
**3c** docked into an active site of FXIa. Blue line indicates pi stacking interaction.

The binding mode common for compounds **3a**-**h** in the active site of factor XIa is shown in Figure 4 using compound **3c** as an example. As can be seen from Figure 4, according to docking, **3c** binds very similarly to **3n**. The main differences are the S2 pocket occupation and the orientation of the central core. In **3c**, the S2 pocket is only partly occupied, and no specific interactions are formed with the residues of the pocket. On the contrary, owing to the presence of a 6-phenyl substituent, **3n** is predicted to engage to the S2 pocket effectively by forming pi-pi stacking and pi-cation interaction with the pocket residues. Besides that, the central pyrrolo[3,2,1-*ij*]quinoline is inverted in the case of **3c** if compared to its orientation in **3n**. Irrespective of the inversion, the scaffold can interact with His-57 via pi stacking interaction in both cases.

## 3. Materials and Methods

The purity of the starting materials and the synthesized compounds, as well as the analysis of reaction mixtures, was monitored by TLC on Silica gel 60 F_254_ plates (Merck, Albany, GA, USA) using chloroform, methanol, or their mixtures as eluent. Chromatograms were developed using UV irradiation, treatment with iodine vapor, or heating the plate. The purity of the obtained compounds is confirmed by HPLC analysis. HPLC analysis was performed using an Agilent 1260 Infinity liquid chromatograph equipped with a UV detector in combination with an Agilent 6230 TOF LC/MS detector. The melting points of the resulting compounds were determined on a Stuart SMP30 instrument. ^1^H NMR spectra were recorded on spectrometers “Bruker AV400”, “Bruker DRX-500”, and “Bruker AV600” operating frequency 400.16, 500.13, and 600.13 MHz, respectively. ^13^C NMR spectra were recorded on a Bruker AV400 spectrometer with an operating frequency of 100.62 MHz. The spectra were recorded at 20 °C using DMSO-d6 as a solvent. For synthetic purposes, commercially available solvents and reagents (SigmaAldrich (St. Louis, MO, USA), Merck, Acros Organics (Geel, Belgium)) were used.

### 3.1. Synthesis

General procedure for the synthesis of methyl 2-(2-(2-(4,4,6-trimethyl-2-oxo-5,6-dihydro-4*H*-pyrrolo[3,2,1-*ij*]quinoline-1(2*H*)-ylidene)hydrazinyl)-4-oxothiazol-5(4*H*)-ylidene)acetates **3a-q**.

The preparation of the target molecules **3a-q** was carried out in two stages by analogy with [58,81]. Initially, to the corresponding 5,6-dihydro-4*H*-pyrrolo[3,2,1-*ij*]quinoline-1,2-dione **1a-q** (1 mmol) and thiosemicarbazide (1.1 mmol), methanol (10 mL) and 1–2 drops of HCl was added. The resulting mixture was refluxed for 1 h. Upon completion of the reaction (control by TLC, eluent CHCl_3_/MeOH 8:1), the volatile components were removed from the reaction and the resulting residue containing intermediate hydrazinocarbothioamide **2** was used in the second stage. In the second stage, hydrazinocarbothioamides **2a-q** (1 mmol) in methanol (12 mL), DMAD (1.1 mmol), and acetic acid (3 mL) were refluxed for 30–60 min. Upon completion of the reaction (monitored by the TLC, eluent CHCl_3_/MeOH 10:1) and cooling of the reaction mixture, the precipitate that formed was filtered off, dried, and, if necessary, recrystallized from a mixture of i-PrOH/AcOH (*v*/*v* 2:1).

*Methyl (Z)-2-(4-oxo-2-(2-((Z)-4,4,6-trimethyl-2-oxo-5,6-dihydro-4H-pyrrolo[3,2,1-ij]quinolin-1(2H)-ylidene)hydrazineyl)thiazol-5(4H)-ylidene)acetate* (**3a**). Orange solid; 0.33 g; yield 80%; m.p. 285–287 °C; ^1^H NMR (400.16 MHz, DMSO-d_6_), δ (ppm): 1.30–1.34 (6H, m, C^6^-CH_3_+C^4^-CH_3_); 1.56 (1H, t, *J* = 12.9 Hz, C^5^-H); 1.70 (3H, s, C^4^-CH_3_); 1.86 (1H, dd, *J* = 13.7 Hz, *J* = 4.5 Hz, C^5^-H); 2.86–2.93 (1H, m, C^6^-H), 3.80 (3H, s, CH_3_O), 6.72 (1H, s, C=CH); 7.04 (1H, t, *J* = 7.6 Hz, CHarom); 7.37 (1H, d, *J* = 7.8 Hz, CHarom); 7.41 (1H, d, *J* = 7.4 Hz, CHarom); 13.35 (1H, br.s, NH). ^13^C NMR, δ (ppm): 18.0, 24.0, 25.3, 26.6, 45.2, 52.5, 53.7, 115.3, 117.9, 119.2, 122.3, 125.3, 129.4, 139.9, 140.8, 142.1, 147.2, 156.4, 165.8. HPLC-HRMS-ESI, m/z ([M + H]+), calcd for C_20_H_20_N_4_O_4_S + H^+^ 413.1279, found 413.1282.

*Methyl (Z)-2-(2-(2-((Z)-8-methoxy-4,4,6-trimethyl-2-oxo-5,6-dihydro-4H-pyrrolo[3,2,1-ij]quinolin-1(2H)-ylidene)hydrazineyl)-4-oxothiazol-5(4H)-ylidene)acetate* (**3b**). Brown solid; 0.34 g; yield 77%; m.p. 232–234 °C; ^1^H NMR (500.13 MHz, DMSO-d_6_), δ (ppm): 1.29–1.32 (6H, m, C^6^-CH_3_+C^4^-CH_3_); 1.54 (1H, t, *J* = 12.9 Hz, C^5^-H); 1.68 (3H, s, C^4^-CH_3_); 1.82–1.87 (1H, m, C^5^-H); 2.82–2.91 (1H, m, C^6^-H), 3.79 (6H, s, 2-CH_3_O), 6.73 (1H, s, C=CH); 6.92–6.93 (1H, m, CHarom); 6.95–6.96 (1H, m, CHarom); 13.47 (1H, br.s, NH). ^13^C NMR, δ (ppm): 18.0, 23.8, 25.5, 26.5, 45.2, 52.5, 53.6, 55.7, 104.0, 112.0, 115.3, 115.6, 116.1, 118.3, 126.6, 134.9, 142.1, 147.5, 155.5, 156.3, 165.8. HPLC-HRMS-ESI, *m*/*z* ([M + H]^+^), calcd for C_21_H_22_N_4_O_5_S + H^+^ 443.1385, found 443.1389.

*Methyl (Z)-2-(2-(2-((Z)-8-chloro-4,4,6-trimethyl-2-oxo-5,6-dihydro-4H-pyrrolo[3,2,1-ij]quinolin-1(2H)-ylidene)hydrazineyl)-4-oxothiazol-5(4H)-ylidene)acetate* (**3c**). Red solid; 0.31 g; yield 69%; m.p. 285–287 °C; ^1^H NMR (600.13 MHz, DMSO-d_6_), δ (ppm): 1.31–1.33 (6H, m, C^6^-CH_3_+C^4^-CH_3_); 1.56 (1H, t, *J* = 12.9 Hz, C^5^-H); 1.69 (3H, s, C^4^-CH_3_); 1.87 (1H, dd, *J* = 13.7 Hz, *J* = 4.5 Hz, C^5^-H); 2.82–2.93 (1H, m, C^6^-H), 3.80 (3H, s, CH_3_O), 6.75 (1H, s, C=CH); 7.36 (1H, s, CHarom); 7.42 (1H, s, CHarom); 13.45 (1H, br.s, NH). ^13^C NMR, δ (ppm): 17.8, 24.1, 25.5, 26.4, 44.9, 52.5, 53.9, 115.5, 118.5, 119.5, 126.6, 127.5, 128.8, 139.5, 141.9, 146.1, 155.9, 165.7, 166.2. HPLC-HRMS-ESI, *m*/*z* ([M + H]^+^), calcd for C_20_H_19_ClN_4_O_4_S + H^+^ 447.0889, found 447.0884.

*Methyl (Z)-2-(2-(2-((Z)-8-bromo-4,4,6-trimethyl-2-oxo-5,6-dihydro-4H-pyrrolo[3,2,1-ij]quinolin-1(2H)-ylidene)hydrazineyl)-4-oxothiazol-5(4H)-ylidene)acetate* (**3d**). Orange solid; 0.35 g; yield 71%; m.p. 185–187 °C; ^1^H NMR (600.13 MHz, DMSO-d_6_), δ (ppm): 1.30–1.33 (6H, m, C^6^-CH_3_+C^4^-CH_3_); 1.55 (1H, t, *J* = 12.9 Hz, C^5^-H); 1.69 (3H, s, C^4^-CH_3_); 1.85 (1H, dd, *J* = 13.7 Hz, *J* = 4.6 Hz, C^5^-H); 2.88–2.93 (1H, m, C^6^-H), 3.80 (3H, s, CH_3_O), 6.73 (1H, s, C=CH); 7.46 (1H, s, CHarom); 7.52 (1H, s, CHarom); 13.45 (1H, br.s, NH). ^13^C NMR, δ (ppm): 17.8, 24.1, 25.5, 26.4, 44.9, 52.5, 53.9, 114.2, 115.5, 119.9, 121.2, 127.8, 131.4, 139.9, 141.8, 145.9, 155.8, 165.7, 166.1, 167.3. HPLC-HRMS-ESI, *m*/*z* ([M + H]^+^), calcd for C_20_H_19_BrN_4_O_4_S + H^+^ 491.0384, found 491.0381.

*Methyl 2-(2-(2-(8-iodo-4,4,6-trimethyl-2-oxo-5,6-dihydro-4H-pyrrolo[3,2,1-ij]quinolin-1(2H)-ylidene)hydrazineyl)-4-oxothiazol-5(4H)-ylidene)acetate* (**3e**). Red solid; 0.41g; yield 76%; m.p. 175–177 °C; A mixture of isomers at the ratio of 11:1. ^1^H NMR (400.16 MHz, DMSO-d_6_), δ (ppm): 1.27–1.32 (6H, m, C^6^-CH_3_+C^4^-CH_3_); 1.33* (3H, s, C^4^-CH_3_); 1.53 (1H, t, *J* = 13.0 Hz, C^5^-H); 1.67, 1.70* (3H, s, C^4^-CH_3_); 1.82, 1.82–1.86* (1H, dd, *J* = 13.7 Hz, *J* = 4.6 Hz, C^5^-H); 2.84–2.91 (1H, m, C^6^-H), 3.79, 3.80* (3H, s, CH_3_O), 6.71, 6.73* (1H, s, C=CH); 7.59 (1H, s, CHarom); 7.64 (1H, s, CHarom); 13.45 (1H, br.s, NH). ^13^C NMR, δ (ppm): 17.8, 24.1, 24.3*, 25.4, 26.4, 26.6*, 44.8*, 44.9, 52.6, 53.9, 85.3, 115.5, 115.8*, 120.2, 126.8, 128.1, 133.7, 137.2, 137.6*, 140.3, 141.9, 145.8, 155.6, 165.7, 166.0. HPLC-HRMS-ESI, *m*/*z* ([M + H]^+^), calcd for C_20_H_19_IN_4_O_4_S + H^+^ 539.0246, found 539.0242.

*Hereinafter, the symbol “*” denotes signals of the minor isomer.

*Methyl (Z)-2-(4-oxo-2-(2-((Z)-4,4,6,8,9-pentamethyl-2-oxo-5,6-dihydro-4H-pyrrolo[3,2,1-ij]quinolin-1(2H)-ylidene)hydrazineyl)thiazol-5(4H)-ylidene)acetate* (**3f**). Orange solid; 0.38 g; yield 86%; m.p. 273–275 °C; ^1^H NMR (500.13 MHz, DMSO-d_6_), δ (ppm): 1.28–1.32 (6H, m, C^6^-CH_3_+C^4^-CH_3_); 1.51 (1H, t, *J* = 12.9 Hz, C^5^-H); 1.69 (3H, s, C^4^-CH_3_); 1.80–1.85 (1H, m, C^5^-H); 2.24 (3H, s, C^8^-CH_3_); 2.47 (3H, s, C^9^-CH_3_); 2.78–2.86 (1H, m, C^6^-H), 3.79 (3H, s, 2-CH_3_O), 6.71 (1H, s, C=CH); 7.14 (1H, s, CHarom); 13.37 (1H, br.s, NH). ^13^C NMR, δ (ppm): 15.0, 18.2, 18.8, 23.9, 25.1, 26.7, 45.6, 52.4, 53.6, 115.5, 115.6, 122.2, 130.2, 130.8, 131.5, 132.8, 138.9, 142.1, 142.5, 156.7, 165.7. HPLC-HRMS-ESI, *m*/*z* ([M + H]^+^), calcd for C_22_H_24_N_4_O_4_S + H^+^ 441.1592, found 441.1590.

*Methyl (Z)-2-(2-(2-(8-chloro-4,4,6,9-tetramethyl-2-oxo-5,6-dihydro-4H-pyrrolo[3,2,1-ij]quinolin-1(2H)-ylidene)hydrazineyl)-4-oxothiazol-5(4H)-ylidene)acetate* (**3g**). Orange solid; 0.37 g; yield 80%; m.p. 264–266 °C; ^1^H NMR (400.16 MHz, DMSO-d_6_), δ (ppm): 1.28–1.33 (6H, m, C^6^-CH_3_+C^4^-CH_3_); 1.53 (1H, t, *J* = 12.9 Hz, C^5^-H); 1.69 (3H, s, C^4^-CH_3_); 1.85 (1H, dd, *J* = 13.7 Hz, *J* = 4.5 Hz, C^5^-H); 2.56 (3H, s, C^9^-CH_3_); 2.82–2.90 (1H, m, C^6^-H), 3.79 (3H, s, CH_3_O), 6.71 (1H, s, C=CH); 7.37 (1H, s, CHarom); 13.40 (1H, br.s, NH). ^13^C NMR, δ (ppm): 15.5, 18.0, 23.9, 25.2, 26.6, 45.0, 52.5, 53.8, 115.5, 116.9, 124.4, 127.7, 128.9, 131.1, 139.7, 142.1, 148.1, 155.9, 165.7, 166.5, 167.5. HPLC-HRMS-ESI, *m*/*z* ([M + H]^+^), calcd for C_21_H_21_ClN_4_O_4_S + H^+^ 461.1046, found 461.1041.

*Methyl (Z)-(2-(2-(2-(8-bromo-4,4,6,9-tetramethyl-2-oxo-5,6-dihydro-4H-pyrrolo[3,2,1-ij]quinolin-1(2H)-ylidene)hydrazineyl)-4-oxothiazol-5(4H)-ylidene)acetate* (**3h**). Orange solid; 0.42 g; yield 83%; m.p. 266–268 °C; ^1^H NMR (400.16 MHz, DMSO-d_6_), δ (ppm): 1.28–1.33 (6H, m, C^6^-CH_3_+C^4^-CH_3_); 1.49–1.57 (1H, m, C^5^-H); 1.69 (3H, s, C^4^-CH_3_); 1.84 (1H, dd, *J* = 13.7 Hz, *J* = 4.6 Hz, C^5^-H); 2.57 (3H, s, C^9^-CH_3_); 2.83–2.90 (1H, m, C^6^-H), 3.79 (3H, s, CH_3_O), 6.69 (1H, s, C=CH); 7.50 (1H, s, CHarom); 13.46 (1H, br.s, NH). ^13^C NMR, δ (ppm): 18.0, 18.5, 23.9, 25.2, 26.5, 45.0, 52.5, 53.8, 115.5, 116.9, 118.4, 124.9, 132.0, 132.8, 140.3, 142.1, 147.8, 155.8, 165.7, 166.7. HPLC-HRMS-ESI, *m*/*z* ([M + H]^+^), calcd for C_21_H_21_BrN_4_O_4_S + H^+^ 505.0541, found 505.0543.

*Methyl (Z)-2-(2-(2-((Z)-6-(4-chlorophenyl)-4,4,6-trimethyl-2-oxo-5,6-dihydro-4H-pyrrolo[3,2,1-ij]quinolin-1(2H)-ylidene)hydrazineyl)-4-oxothiazol-5(4H)-ylidene)acetate* (**3i**). Orange solid; 0.40 g; yield 76%; m.p. 165–167 °C; ^1^H NMR (600.13 MHz, DMSO-d_6_), δ (ppm): 0.75 (3H, s, C^6^-CH_3_); 1.62 (3H, s, C^4^-CH_3_); 1.68 (3H, s, C^4^-CH_3_); 2.13 (1H, d, *J* = 14.4 Hz, C^5^-H); 2.47 (1H, d, *J* = 14.4 Hz, C^5^-H); 3.81 (3H, s, CH_3_O), 6.75 (1H, s, C=CH); 7.13 (2H, d, *J* = 8.4 Hz, CHarom); 7.17 (1H, t, *J* = 7.6 Hz, CHarom); 7.32 (2H, d, *J* = 8.5 Hz, CHarom); 7.45 (1H, d, *J* = 7.9 Hz, CHarom); 7.56 (1H, d, *J* = 7.4 Hz, CHarom); 13.43 (1H, br.s, NH). ^13^C NMR, δ (ppm): 24.7, 27.6, 30.2, 39.1, 50.7, 52.5, 53.7, 115.3, 118.7, 120.0, 122.5, 125.5, 128.1, 128.5, 130.8, 131.1, 140.6, 142.1, 146.9, 147.0, 156.1, 165.8, 166.2. HPLC-HRMS-ESI, m/z ([M + H]^+^), calcd for C_26_H_23_ClN_4_O_4_S + H^+^ 523.1203, found 523.1199.

*Methyl 2-(2-(2-(6-(4-chlorophenyl)-4,4,6,8-tetramethyl-2-oxo-5,6-dihydro-4H-pyrrolo[3,2,1-ij]quinolin-1(2H)-ylidene)hydrazineyl)-4-oxothiazol-5(4H)-ylidene)acetate* (**3j**). Orange solid; 0.46 g; yield 86%; m.p. 193–195 °C; a mixture of isomers at the ratio of 4:1. ^1^H NMR (400.16 MHz, DMSO-d_6_), δ (ppm): 0.75, 0.77* (3H, s, C^6^-CH_3_); 1.59, 1.62* (3H, s, C^4^-CH_3_); 1.67 (3H, s, C^4^-CH_3_); 2.10 (1H, d, *J* = 14.5 Hz, C^5^-H); 2.33*, 2.37 (3H, s, C^8^-CH_3_); 2.43, 2.44* (1H, d, *J* = 14.4 Hz, C^5^-H); 3.81 (3H, s, CH_3_O), 6.73 (1H, s, C=CH); 7.10–7.14*, 7.11–7.15 (2H, m, CHarom); 7.26 (1H, s, CHarom); 7.32 (2H, d, *J* = 8.7 Hz, CHarom); 7.37 (1H, s, CHarom); 13.33 (1H, br.s, NH). ^13^C NMR, δ (ppm): 20.7, 20.8, 24.7, 24.7, 27.6, 27.8*, 30.2, 51.0, 52.4, 52.6, 53.5*, 53.6, 115.1, 115.4*, 118.7, 120.4, 125.2*, 125.4, 127.6*, 128.1, 128.6, 130.7, 131.3*, 131.4, 131.7, 138.5, 139.1*, 142.4, 147.0, 156.2, 162.0, 165.9, 166.5. HPLC-HRMS-ESI, *m*/*z* ([M + H]^+^), calcd for C_27_H_25_ClN_4_O_4_S + H^+^ 537.1359, found 537.1360.

*Methyl (Z)-2-(2-(2-((Z)-8-chloro-6-(4-chlorophenyl)-4,4,6-trimethyl-2-oxo-5,6-dihydro-4H-pyrrolo[3,2,1-ij]quinolin-1(2H)-ylidene)hydrazineyl)-4-oxothiazol-5(4H)-ylidene)acetate* (**3k**). Orange solid; 0.44 g; yield 79%; m.p. 210–212 °C; ^1^H NMR (400.16 MHz, DMSO-d_6_), δ (ppm): 0.75 (3H, s, C^6^-CH_3_); 1.60 (3H, s, C^4^-CH_3_); 1.69 (3H, s, C^4^-CH_3_); 2.11 (1H, d, *J* = 14.3 Hz, C^5^-H); 2.46 (1H, d, *J* = 14.4 Hz, C^5^-H); 3.81 (3H, s, CH_3_O), 6.75 (1H, s, C=CH); 7.14 (2H, d, *J* = 8.7 Hz, CHarom); 7.34 (2H, d, *J* = 8.8 Hz, CHarom); 7.48–7.51 (2H, m, CHarom); 13.34 (1H, br.s, NH). ^13^C NMR, δ (ppm): 24.7, 24.7, 27.5, 30.0, 50.7, 52.5, 52.6, 53.9, 115.6, 115.6, 119.4, 119.5, 120.4, 126.8, 127.6, 128.2, 128.5, 130.2, 130.2, 130.9, 139.5, 141.9, 146.0, 146.3, 155.7, 165.8, 166.2, 167.4. HPLC-HRMS-ESI, *m*/*z* ([M + H]^+^), calcd for C_26_H_22_Cl_2_N_4_O_4_S + H^+^ 557.0813, found 557.0810.

*Methyl (Z)-2-(2-(2-((Z)-8-bromo-6-(4-chlorophenyl)-4,4,6-trimethyl-2-oxo-5,6-dihydro-4H-pyrrolo[3,2,1-ij]quinolin-1(2H)-ylidene)hydrazineyl)-4-oxothiazol-5(4H)-ylidene)acetate* (**3l**). Orange solid; 0.49 g; yield 81%; m.p. 208–210 °C; ^1^H NMR (400.16 MHz, DMSO-d_6_), δ (ppm): 0.75 (3H, s, C^6^-CH_3_); 1.60 (3H, s, C^4^-CH_3_); 1.69 (3H, s, C^4^-CH_3_); 2.11 (1H, d, *J* = 14.6 Hz, C^5^-H); 2.45 (1H, d, *J* = 14.4 Hz, C^5^-H); 3.81 (3H, s, CH_3_O), 6.75 (1H, s, C=CH); 7.14 (2H, d, *J* = 8.5 Hz, CHarom); 7.34 (2H, d, *J* = 8.5 Hz, CHarom); 7.59–7.62 (2H, m, CHarom); 13.49 (1H, br.s, NH). ^13^C NMR, δ (ppm): 24.7, 24.8, 27.5, 29.9, 50.7, 52.5, 52.6, 53.9, 114.4, 115.6, 115.6, 120.8, 122.2, 128.2, 128.5, 130.9, 132.9, 139.9, 141.9, 145.8, 146.4, 155.6, 165.8, 166.2. HPLC-HRMS-ESI, *m*/*z* ([M + H]^+^), calcd for C_26_H_22_BrClN_4_O_4_S + H^+^ 601.0308, found 601.0303.

*Methyl 2-(2-(2-(6-(4-chlorophenyl)-8-fluoro-4,4,6-trimethyl-2-oxo-5,6-dihydro-4H-pyrrolo[3,2,1-ij]quinolin-1(2H)-ylidene)hydrazineyl)-4-oxothiazol-5(4H)-ylidene)acetate* (**3m**). Orange solid; 0.46 g; yield 85%; m.p. 181–183 °C; a mixture of isomers at the ratio of 3:1. ^1^H NMR (400.16 MHz, DMSO-d_6_), δ (ppm): 0.74, 0.76* (3H, s, C^6^-CH_3_); 1.60, 1.63* (3H, s, C^4^-CH_3_); 1.68 (3H, s, C^4^-CH_3_); 2.11, 2.12* (1H, d, *J* = 14.5 Hz, C^5^-H); 2.46, 2.47* (1H, d, *J* = 14.5 Hz, C^5^-H); 3.81 (3H, s, CH_3_O), 6.72, 6.74* (1H, s, C=CH); 7.13*, 7.14 (2H, d, *J* = 8.7 Hz, CHarom); 7.30–7.37 (4H, m, CHarom); 13.49 (1H, br.s, NH). ^13^C NMR, δ (ppm): 24.6, 24.9*, 27.5, 27.7*, 30.1, 50.5*, 50.7, 52.5, 52.6*, 53.7*, 53.8, 107.0, 107.3, 113.9*, 114.2*, 115.5, 115.8*, 117.3, 117.5, 117.7*, 117.9*, 119.8, 119.9, 127.0*, 127.2, 127.3, 128.1, 128.5, 130.9, 137.0, 137.7*, 142.0, 146.4, 155.9, 156.9*, 157.3, 159.3*, 159.7, 161.7. 165.7, 166.2, 167.2. HPLC-HRMS-ESI, *m*/*z* ([M + H]^+^), calcd for C_26_H_22_ClFN_4_O_4_S + H^+^ 541.1108, found 541.1111.

*Methyl (Z)-2-(4-oxo-2-(2-((Z)-4,4,6-trimethyl-2-oxo-6-phenyl-5,6-dihydro-4H-pyrrolo[3,2,1-ij]quinolin-1(2H)-ylidene)hydrazineyl)thiazol-5(4H)-ylidene)acetate* (**3n**). Orange solid; 0.43 g; yield 88%; m.p. 232–234 °C; ^1^H NMR (500.13 MHz, DMSO-d_6_), δ (ppm): 0.72 (3H, s, C^6^-CH_3_); 1.61 (3H, s, C^4^-CH_3_); 1.70 (3H, s, C^4^-CH_3_); 2.13 (1H, d, *J* = 14.3 Hz, C^5^-H); 2.46–2.51 (m, C^5^-H+H_2_O); 3.81 (3H, s, CH_3_O), 6.75 (1H, s, C=CH); 7.10 (2H, d, *J* = 7.5 Hz, CHarom); 7.15–7.19 (2H, m, CHarom); 7.24–7.28 (2H, m, CHarom); 7.45 (1H, d, *J* = 7.7 Hz, CHarom); 7.56 (1H, d, *J* = 7.3 Hz, CHarom); 13.34 (1H, br.s, NH). ^13^C NMR, δ (ppm): 24.6, 27.7, 30.4, 51.0, 52.5, 53.8, 115.4, 119.0, 119.9, 122.4, 122.7, 126.1, 126.5, 128.1, 129.4, 131.3, 140.7, 142.1, 147.7, 147.8, 156.2, 160.2, 165.8. HPLC-HRMS-ESI, *m*/*z* ([M + H]^+^), calcd for C_26_H_24_N_4_O_4_S + H^+^ 489.1592, found 489.1590.

*Methyl (Z)-2-(2-(2-((Z)-8-chloro-4,4,6-trimethyl-2-oxo-6-phenyl-5,6-dihydro-4H-pyrrolo[3,2,1-ij]quinolin-1(2H)-ylidene)hydrazineyl)-4-oxothiazol-5(4H)-ylidene)acetate* (**3o**). Orange solid; 0.44 g; yield 84%; m.p. 165–167 °C; ^1^H NMR (400.16 MHz, DMSO-d_6_), δ (ppm): 0.72 (3H, s, C^6^-CH_3_); 1.60 (3H, s, C^4^-CH_3_); 1.71 (3H, s, C^4^-CH_3_); 2.11 (1H, d, *J* = 14.4 Hz, C^5^-H); 2.45–2.51 (m, C^5^-H+H_2_O); 3.82 (3H, s, CH_3_O), 6.76 (1H, s, C=CH); 7.10 (2H, d, *J* = 7.6 Hz, CHarom); 7.17–7.21 (1H, m, CHarom); 7.26–7.31 (2H, m, CHarom); 7.48–7.51 (2H, m, CHarom); 13.35 (1H, br.s, NH). ^13^C NMR, δ (ppm): 24.6, 27/5, 30.0, 50.8, 52.5, 53.9, 115.5, 119.3, 120.3, 126.2, 126.5, 126.7, 128.2, 128.3, 130.3, 139.5, 147.3, 155.8, 165.8. HPLC-HRMS-ESI, *m*/*z* ([M + H]^+^), calcd for C_26_H_23_ClN_4_O_4_S + H^+^ 523.1203, found 523.1198.

*Methyl (Z)-2-(2-(2-((Z)-8-bromo-4,4,6-trimethyl-2-oxo-6-phenyl-5,6-dihydro-4H-pyrrolo[3,2,1-ij]quinolin-1(2H)-ylidene)hydrazineyl)-4-oxothiazol-5(4H)-ylidene)acetate* (**3p**). Orange solid; 0.38 g; yield 67%; m.p. 208–210 °C; ^1^H NMR (500.13 MHz, DMSO-d_6_), δ (ppm): 0.72 (3H, s, C^6^-CH_3_); 1.60 (3H, s, C^4^-CH_3_); 1.71 (3H, s, C^4^-CH_3_); 2.11 (1H, d, *J* = 14.4 Hz, C^5^-H); 2.46 (1H, d, *J* = 14.4 Hz, C^5^-H); 3.82 (3H, s, CH_3_O), 6.76 (1H, s, C=CH); 7.10 (2H, d, *J* = 7.7 Hz, CHarom); 7.19 (1H, t, *J* = 7.3 Hz, CHarom); 7.28 (2H, d, *J* = 7.7 Hz, CHarom); 7.60–7.62 (2H, m, CHarom); 13.44 (1H, br.s, NH). ^13^C NMR, δ (ppm): 24.5, 24.6, 27.5, 30.0, 50.9, 52.5, 54.0, 114.3, 115.6, 115.6, 120.6, 122.0, 126.2, 126.5, 128.3, 128.7, 133.0, 139.9, 141.9, 145.9, 147.3, 155.6, 165.8. HPLC-HRMS-ESI, *m*/*z* ([M + H]^+^), calcd for C_26_H_23_BrN_4_O_4_S + H^+^ 567.0697, found 567.0699.

*Methyl (Z)-2-(2-(2-((Z)-8-fluoro-4,4,6-trimethyl-2-oxo-6-phenyl-5,6-dihydro-4H-pyrrolo[3,2,1-ij]quinolin-1(2H)-ylidene)hydrazineyl)-4-oxothiazol-5(4H)-ylidene)acetate* (**3q**). Orange solid; 0.39 g; yield 77%; m.p. 171–173 °C; ^1^H NMR (400.16 MHz, DMSO-d_6_), δ (ppm): 0.69 (3H, s, C^6^-CH_3_); 1.60 (3H, s, C^4^-CH_3_); 1.68 (3H, s, C^4^-CH_3_); 2.10 (1H, d, *J* = 14.3 Hz, C^5^-H); 2.44–2.51 (m, C^5^-H+H_2_O); 3.80 (3H, s, CH_3_O), 6.72 (1H, s, C=CH); 7.11 (2H, d, *J* = 7.8 Hz, CHarom); 7.18 (1H, t, *J* = 7.3 Hz, CHarom); 7.24–7.36 (4H, m, CHarom); 13.43 (1H, br.s, NH). ^13^C NMR, δ (ppm): 24.5, 27.6, 30.2, 50.8, 52.5, 53.8, 106.9, 107.1, 115.5, 117.5, 117.7, 119.6, 119.7, 126.2, 126.4, 127.8, 127.9, 128.2, 137.1, 141.9, 146.6, 147.3, 155.9, 157.3, 159.7, 165.7, 166.1, 167.1. HPLC-HRMS-ESI, *m*/*z* ([M + H]^+^), calcd for C_26_H_23_FN_4_O_4_S + H^+^ 507.1498, found 507.1502.

### 3.2. In Vitro Assays

The inhibition of the blood clotting factors Xa and XIa and thrombin by the synthesized compounds **3a-q** was studied by measuring the kinetics of hydrolysis of substrates specific for each of these enzymes in the presence of the compounds. A specific low-molecular-weight chromogenic substrate S2765 (Z-D-Arg-Gly-Arg-pNA·2HCl) was used in the case of factor Xa, substrate S2366 (pyroGlu-Pro-ArgpNA·HCl) (both from Chromogenix, USA) was used for factor XIa, and Tos-Gly-Pro-Arg-pNa was used for thrombin.

A buffer containing 140 m*M* NaCl, 20 mM HEPES, and 0.1% PEG 6000 (pH 8.0) was placed in the wells of a 96-well plate, followed by the addition of factor Xa or XIa (final concentration 5 nmol·L^−1^), or thrombin (final concentration 2.5 nmol·L^−1^) the substrate S2765 or S2366 (final concentration 200 μmol·L^−1^), or Tos-Gly-Pro-Arg-pNa (final concentration 100 μmol·L^−1^) and a solution of the test compound in DMSO (final concentration 30 μmol·L^−1^, the DMSO content in the well was no more than 2%). The kinetics of the formation of *p*-nitroaniline was measured using an Eppendorf PlateReader AF2200 microplate reader (Eppendorf, Hamburg, Germany) by the absorption of light with a wavelength of 405 nm. The initial rate of substrate degradation was determined from the initial slope of the 4-nitroaniline formation curve. The rate of substrate degradation by the enzyme in the presence of the inhibitor was expressed as a percentage relative to the rate of substrate degradation in the absence of the inhibitor. The results are presented in Table 1 and Table 2. The data obtained were processed using the GraphPad Prism 8.0.1 (244) and OriginPro 8 software.

### 3.3. Molecular Docking Studies

To obtain insights about the binding modes, we executed docking for some of identified inhibitors into an active site of FXa and FXIa using an in-house SOL docking program [90]. It applies a genetic algorithm for exploring the conformational space of a ligand in the frame of the rigid protein approximation. The SOL conducts grid-based docking, where a series of grids is generated through the assistance of an auxiliary program known as a SOLGRID. To be consistent with the SOLGRID, the input protein structure should contain atom types parametrized in MMFF94 [91] (in-house version slightly reduced to types relevant for a drug-like space). This is mainly since the SOL scoring function is based on this force field. The function represents a linear combination of weighted terms that account for different types of interactions occurring between a ligand and a protein. They include electrostatic and van der Waals interactions inferred from MMFF94 force field, as well as the desolvation energy term calculated using a simplified generalized Born implicit solvent model [92] and the entropy term that is estimated as the reduction in torsional degrees of freedom for the ligand. Similar to other widely used docking programs with stochastic sampling methods, upon completion of the docking process the SOL performs ranking and clustering of the docking solutions to organize the identified ligand poses based on their respective scores and conformations. This analysis helps to evaluate the reliability of the conformational space exploration under the specific genetic algorithm parameters chosen for the ligand. The use of the SOL for virtual screening for identification of active molecules for different targets can be found in [58,93,94,95,96].

All compounds subjected to docking were prepared using Marvin pKa protonation plugin [97] (to generate protomers relevant for pH 7.4) and Open Babel [98] (to generate conformers). The protein structure of FXa was taken from the 3CEN complex and the 4CRC complex was used to prepare FXIa protein. The protonation of both protein structures as well as atom parametrization was carried out in the in-house preparation program, Aplite. Validation of the prepared protein models implied docking of the corresponding co-crystalized ligand. For both FXa and FXIa, the reproduction of co-crystallized ligand conformation with an RMSD value less than 1.5 Å was obtained.

## 4. Conclusions

Seventeen new hybrid molecules—methyl 2-(2-(2-(4,4,6-trimethyl-2-oxo-5,6-dihydro-4*H*-pyrrolo[3,2,1-*ij*]quinoline-1(2*H*)-ylidene)hydrazinyl)-4-oxothiazol-5(4*H*)-ylidene)acetates were synthesized and evaluated for their inhibitory activity toward FXa and FXIa. The synthesis was performed by a two-step approach, where the corresponding hydrazinocarbothioamides were obtained by condensation of pyrrolo[3,2,1-*ij*]quinoline-1,2-diones with thiosemicarbazide, followed by a heterocyclization reaction with DMAD, which led to the target structures. The in vitro experimental data on inhibitory activity against factors Xa and XIa demonstrate the high potential of these compounds to be dual inhibitors. The rather low values of the inhibitory activity of the described compounds against thrombin determine wide possibilities for their targeted optimization for the further search for new generation anticoagulants with a reduced risk of possible bleeding.

## Figures and Tables

**Table 1:** Results of docking and the experimental measurements of anticoagulant activity of compounds **3a-q** against factors Xa and XIa. The SOL score is the estimation of the protein–ligand-binding free energy calculated by the SOL docking program.

№	Factor Xa, SOL Score, kcal/mol	Factor XIa, SOL Score, kcal/mol	Percent Inhibition at 30 μM		IC_50_, μM	
			Xa	XIa	Xa	XIa
**3a**	−5.39	−4.16	−1	−11	–	–
**3b**	−5.49	−4.46	91	60	5.76 ± 1.08	8.10 ± 0.20
**3c**	−5.76	−4.69	87	93	2.84 ± 0.04	12.20 ± 0.20
**3d**	−5.86	−4.85	77	96	–	–
**3e**	−5.93	−4.86	99	100	4.23 ± 0.31	8.03 ± 0.68
**3f**	−5.91	−4.78	90	87	4.02 ± 0.60	7.97 ± 0.63
**3g**	−5.90	−4.79	24	62	–	–
**3h**	−5.98	−4.90	−1	−16	–	–
**3i**	−6.03	−4.73	87	65	–	–
**3j**	−6.10	−4.55	64	83	–	–
**3k**	−6.35	−5.00	95	79	1.17 ± 0.26	4.59 ± 0.54
**3l**	−5.76	−5.13	92	90	1.74 ± 0.21	3.61 ± 1.70
**3m**	−6.14	−4.43	63	16	–	–
**3n**	−5.42	−4.44	93	85	1.86 ± 0.07	11.46 ± 0.98
**3o**	−5.86	−4.74	87	90	1.34 ± 0.16	7.21 ± 0.22
**3p**	−5.97	−4.93	83	97	1.83 ± 0.05	3.87 ± 0.33
**3q**	−5.78	−4.22	85	84	–	–
Rivaroxaban	−6.89		94	8	0.007 ± 0.001	

**Table 2:** Results of docking and the experimental measurements of anticoagulant activity of compounds **3a-q** against thrombin. The SOL score is the estimation of the protein–ligand-binding free energy calculated by the SOL docking program.

№	Thrombin, SOL Score, kcal/mol	Percent Inhibition of Thrombin at 30 μM
**3a**	−5.53	2
**3b**	−5.78	24
**3c**	−6.01	29
**3d**	−5.94	31
**3e**	−5.72	96
**3f**	−5.68	43
**3g**	−5.72	20
**3h**	−6.12	−6
**3i**	−5.27	39
**3j**	−5.43	28
**3k**	−5.60	43
**3l**	−5.70	48
**3m**	−5.19	18
**3n**	−5.97	27
**3o**	−6.28	71
**3p**	−6.55	45
**3q**	−5.92	26
Argatroban	-	99

## Data Availability

The data presented in this study are available in article and Supplementary Materials.

## Supplementary Materials

The following supporting information can be downloaded at: https://www.mdpi.com/article/10.3390/molecules29020373/s1. The copies of ^1^H- and ^13^C-NMR spectra and data of HPLC-MS-ESI analysis for all new synthesized compounds have been submitted along with the manuscript. The dependence of inhibition of Factor Xa- and XIa-induced chromogenic substrate hydrolysis on the concentration of compounds **3b-c**, **e-f**, **k-l**, **n-p** is also presented. Supplementary Materials containing ^1^H and ^13^C NMR, data of HPLC-MS-ESI analysis for new synthesized compounds (Figures S1–S51), the dependence of inhibition of Factor Xa- and XIa-induced chromogenic substrate hydrolysis on the concentration of compounds (Figures S52–S69) and LCMS analysis of the reaction mass (Figure S70).
