# Supplementary material for: Design, Synthesis, and Evaluation of New Hybrid Derivatives of 5,6-Dihydro-4H-pyrrolo[3,2,1-ij]quinolin-2(1H)-one as Potential Dual Inhibitors of Blood Coagulation Factors Xa and XIa
Source: Molecules. 2024 Jan 11;29(2):373. doi: 10.3390/molecules29020373 (PMC10818416; doi:10.3390/molecules29020373)
Supplement: Supplementary file 1 [file molecules-29-00373-s001.zip › molecules-2717899-supplementary.pdf]

Electronic Supplementary Material file

Design, synthesis and evaluation of new hybrid derivatives of 5,6-dihydro-4*H*-pyrrolo[3,2,1-*ij*]quinolin-2(1*H*)-one as potential dual inhibitors of blood coagulation factors Xa and XIa

Anna A. Skoptsova [1], Athina Geronikaki [2], Nadezhda P. Novichikhina [1], Alexey V. Sulimov [3], Ivan S. Ilin [3], Vladimir B. Sulimov [3], Georgii A. Bykov [4], Nadezhda A. Podoplelova [5], Oleg V. Pyankov [6] and Khidmet S. Shikhaliev[1]

[1] Department of Organic Chemistry, Faculty of Chemistry, Voronezh State University, 1 Universitetskaya Sq., 394018 Voronezh, Russia
[2] School of Pharmacy, Aristotle University of Thessaloniki, 54124 Thessaloniki, Greece
[3] Research Computing Center, Lomonosov Moscow State University, 119992 Moscow, Russia
[4] Department of Biophysics at the Faculty of Physics, Lomonosov Moscow State University, 119992 Moscow, Russia
[5] Center for Theoretical Problems of Physicochemical Pharmakology, 119991 Moscow, Russia
[6] State Research Center of Virology and Biotechnology "Vector", 630559 Koltsovo, Russia;
* Correspondence: geronik@pharm.auth.gr (A.G.); shikh1961@yandex.ru (K.S.S.)

Contents.

Copies of spectral data of synthesized compounds S4

^{1}H, ^{13}C NMR and data HPLC-HRMS-ESI spectra of methyl (Z)-2-(4-oxo-2-(2-((Z)-4,4,6-trimethyl-2-oxo-5,6-dihydro-4*H*-pyrrolo[3,2,1-*ij*]quinolin-1(2*H*)-ylidene)hydrazineyl)thiazol-5(4*H*)-ylidene)acetate 3a S4

^{1}H, ^{13}C NMR and data HPLC-HRMS-ESI spectra of methyl (Z)-2-(2-(2-((Z)-8-methoxy-4,4,6-trimethyl-2-oxo-5,6-dihydro-4*H*-pyrrolo[3,2,1-*ij*]quinolin-1(2*H*)-ylidene)hydrazineyl)-4-oxothiazol-5(4*H*)-ylidene)acetate 3b S6

^{1}H, ^{13}C NMR and data HPLC-HRMS-ESI spectra of methyl (Z)-2-(2-(2-((Z)-8-chloro-4,4,6-trimethyl-2-oxo-5,6-dihydro-4*H*-pyrrolo[3,2,1-*ij*]quinolin-1(2*H*)-ylidene)hydrazineyl)-4-oxothiazol-5(4*H*)-ylidene)acetate 3c S8

^{1}H, ^{13}C NMR and data HPLC-HRMS-ESI spectra of methyl (Z)-2-(2-(2-((Z)-8-bromo-4,4,6-trimethyl-2-oxo-5,6-dihydro-4*H*-pyrrolo[3,2,1-*ij*]quinolin-1(2*H*)-ylidene)hydrazineyl)-4-oxothiazol-5(4*H*)-ylidene)acetate 3d S10

^{1}H, ^{13}C NMR and data HPLC-HRMS-ESI spectra of methyl 2-(2-(2-(8-iodo-4,4,6-trimethyl-2-oxo-5,6-dihydro-4*H*-pyrrolo[3,2,1-*ij*]quinolin-1(2*H*)-ylidene)hydrazineyl)-4-oxothiazol-5(4*H*)-ylidene)acetate 3e S12

^{1}H, ^{13}C NMR and data HPLC-HRMS-ESI spectra of methyl (Z)-2-(4-oxo-2-(2-((Z)-4,4,6,8,9-pentamethyl-2-oxo-5,6-dihydro-4*H*-pyrrolo[3,2,1-*ij*]quinolin-1(2*H*)-ylidene)hydrazineyl)thiazol-5(4*H*)-ylidene)acetate 3f S14

^{1}H, ^{13}C NMR and data HPLC-HRMS-ESI spectra of methyl (Z)-2-(2-(2-(8-chloro-4,4,6,9-tetramethyl-2-oxo-5,6-dihydro-4*H*-pyrrolo[3,2,1-*ij*]quinolin-1(2*H*)-ylidene)hydrazineyl)-4-oxothiazol-5(4*H*)-ylidene)acetate 3g S16

^{1}H, ^{13}C NMR and data HPLC-HRMS-ESI spectra of methyl (Z)-(2-(2-(2-(8-bromo-4,4,6,9-tetramethyl-2-oxo-5,6-dihydro-4*H*-pyrrolo[3,2,1-*ij*]quinolin-1(2*H*)-ylidene)hydrazineyl)-4-oxothiazol-5(4*H*)-ylidene)acetate 3h S18

^{1}H, ^{13}C NMR and data HPLC-HRMS-ESI spectra of methyl (Z)-2-(2-(2-((Z)-6-(4-chlorophenyl)-4,4,6-trimethyl-2-oxo-5,6-dihydro-4*H*-pyrrolo[3,2,1-*ij*]quinolin-1(2*H*)-ylidene)hydrazineyl)-4-oxothiazol-5(4*H*)-ylidene)acetate 3i S20

^{1}H, ^{13}C NMR and data HPLC-HRMS-ESI spectra of methyl 2-(2-(2-(6-(4-chlorophenyl)-4,4,6,8-tetramethyl-2-oxo-5,6-dihydro-4*H*-pyrrolo[3,2,1-*ij*]quinolin-1(2*H*)-ylidene)hydrazineyl)-4-oxothiazol-5(4*H*)-ylidene)acetate 3j S22

^{1}H, ^{13}C NMR and data HPLC-HRMS-ESI spectra of methyl (Z)-2-(2-(2-((Z)-8-chloro-6-(4-chlorophenyl)-4,4,6-trimethyl-2-oxo-5,6-dihydro-4*H*-pyrrolo[3,2,1-*ij*]quinolin-1(2*H*)-ylidene)hydrazineyl)-4-oxothiazol-5(4*H*)-ylidene)acetate 3k S24

^{1}H, ^{13}C NMR and data HPLC-HRMS-ESI spectra of methyl (Z)-2-(2-(2-((Z)-8-bromo-6-(4-chlorophenyl)-4,4,6-trimethyl-2-oxo-5,6-dihydro-4*H*-pyrrolo[3,2,1-*ij*]quinolin-1(2*H*)-ylidene)hydrazineyl)-4-oxothiazol-5(4*H*)-ylidene)acetate 3l S26

^{1}H, ^{13}C NMR and data HPLC-HRMS-ESI spectra of methyl 2-(2-(2-(6-(4-chlorophenyl)-8-fluoro-4,4,6-trimethyl-2-oxo-5,6-dihydro-4*H*-pyrrolo[3,2,1-*ij*]quinolin-1(2*H*)-ylidene)hydrazineyl)-4-oxothiazol-5(4*H*)-ylidene)acetate 3m S28

^{1}H, ^{13}C NMR and data HPLC-HRMS-ESI spectra of methyl (Z)-2-(4-oxo-2-(2-((Z)-4,4,6-trimethyl-2-oxo-6-phenyl-5,6-dihydro-4*H*-pyrrolo[3,2,1-*ij*]quinolin-1(2*H*)-ylidene)hydrazineyl)thiazol-5(4*H*)-ylidene)acetate 3n S30

^{1}H, ^{13}C NMR and data HPLC-HRMS-ESI spectra of methyl (Z)-2-(2-(2-((Z)-8-chloro-4,4,6-trimethyl-2-oxo-6-phenyl-5,6-dihydro-4*H*-pyrrolo[3,2,1-*ij*]quinolin-1(2*H*)-ylidene)hydrazineyl)-4-oxothiazol-5(4*H*)-ylidene)acetate 3o S32

^{1}H, ^{13}C NMR and data HPLC-HRMS-ESI spectra of methyl (Z)-2-(2-(2-((Z)-8-bromo-4,4,6-trimethyl-2-oxo-6-phenyl-5,6-dihydro-4*H*-pyrrolo[3,2,1-*ij*]quinolin-1(2*H*)-ylidene)hydrazineyl)-4-oxothiazol-5(4*H*)-ylidene)acetate 3p S34

^{1}H, ^{13}C NMR and data HPLC-HRMS-ESI spectra of methyl (Z)-2-(2-(2-((Z)-8-fluoro-4,4,6-trimethyl-2-oxo-6-phenyl-5,6-dihydro-4*H*-pyrrolo[3,2,1-*ij*]quinolin-1(2*H*)-ylidene)hydrazineyl)-4-oxothiazol-5(4*H*)-ylidene)acetate 3q S36

The dependence of inhibition of factor Xa- and XIa-induced chromogenic substrate hydrolysis on the compound concentration. S38

The dependence of inhibition of factor Xa- and XIa-induced chromogenic substrate hydrolysis on the concentration of 3b S38

The dependence of inhibition of factor Xa- and XIa-induced chromogenic substrate hydrolysis on the concentration of 3c... S39

The dependence of inhibition of factor Xa- and XIa-induced chromogenic substrate hydrolysis on the concentration of 3e... S40

The dependence of inhibition of factor Xa- and XIa-induced chromogenic substrate hydrolysis on the concentration of 3f... S41

The dependence of inhibition of factor Xa- and XIa-induced chromogenic substrate hydrolysis on the concentration of 3k S42

The dependence of inhibition of factor Xa- and XIa-induced chromogenic substrate hydrolysis on the concentration of 3l... S43

The dependence of inhibition of factor Xa- and XIa-induced chromogenic substrate hydrolysis on the concentration of 3n S44

The dependence of inhibition of factor Xa- and XIa-induced chromogenic substrate hydrolysis on the concentration of 3o S45

The dependence of inhibition of factor Xa- and XIa-induced chromogenic substrate hydrolysis on the concentration of 3p S46

LCMS analysis of the reaction mass after 1 h of interaction of compound 1b with thiosemicarbazide to obtain 2b... S47

Copies of spectral data of synthesized compounds

^{1}H, ^{13}C NMR and data HPLC-HRMS-ESI spectra of methyl (Z)-2-(4-oxo-2-(2-((Z)-4,4,6-trimethyl-2-oxo-5,6-dihydro-4*H*-pyrrolo[3,2,1-*ij*]quinolin-1(2*H*)-ylidene)hydrazineyl)thiazol-5(4*H*)-ylidene)acetate 3a

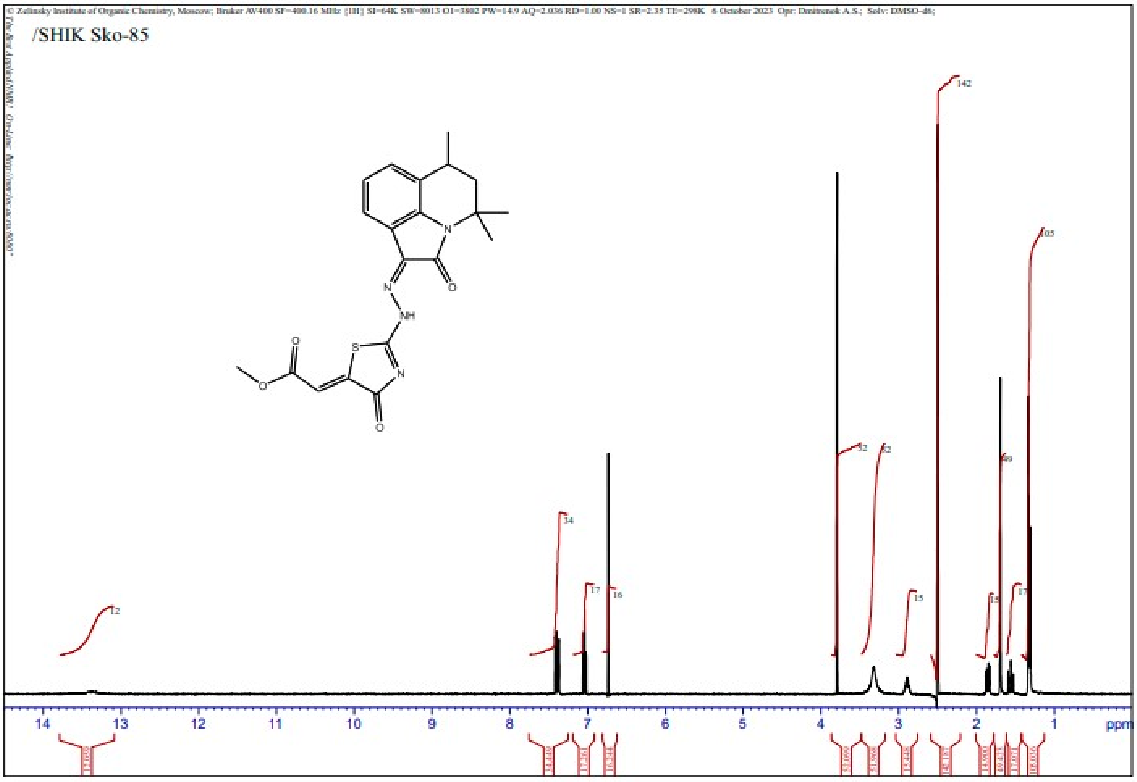

Figure S1. ^{1}H NMR spectrum of compound **3a**

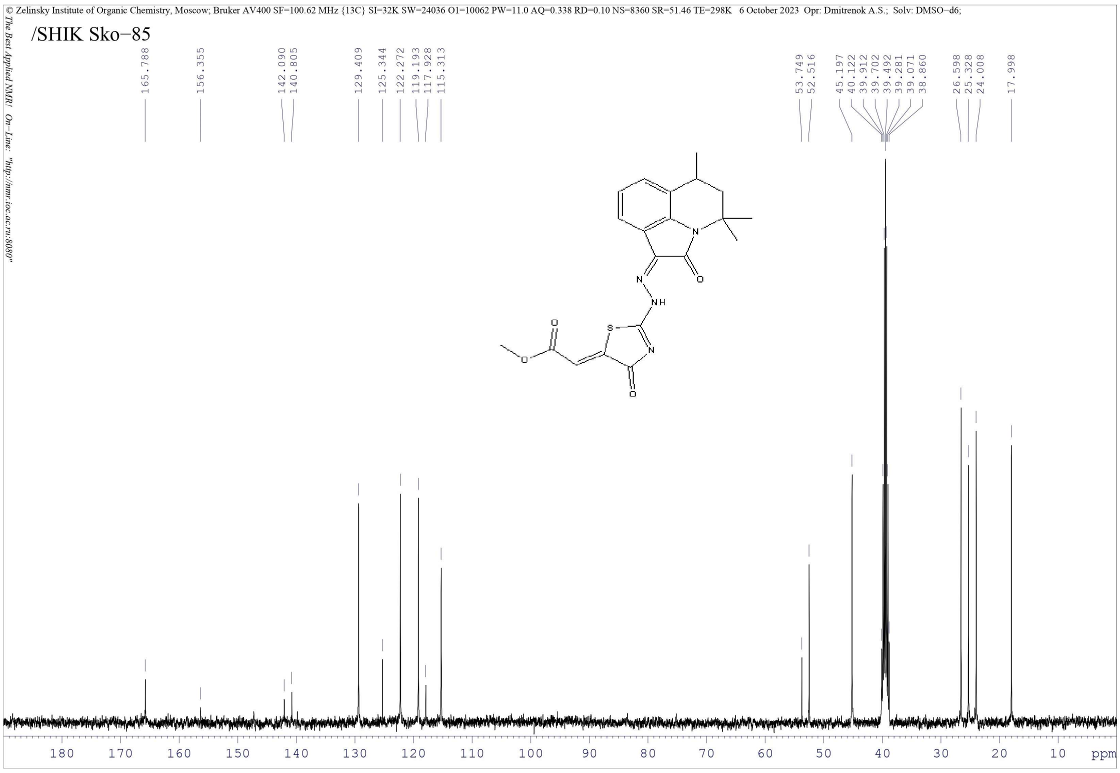

Figure S2. ^{13}C NMR spectrum of compound **3a**

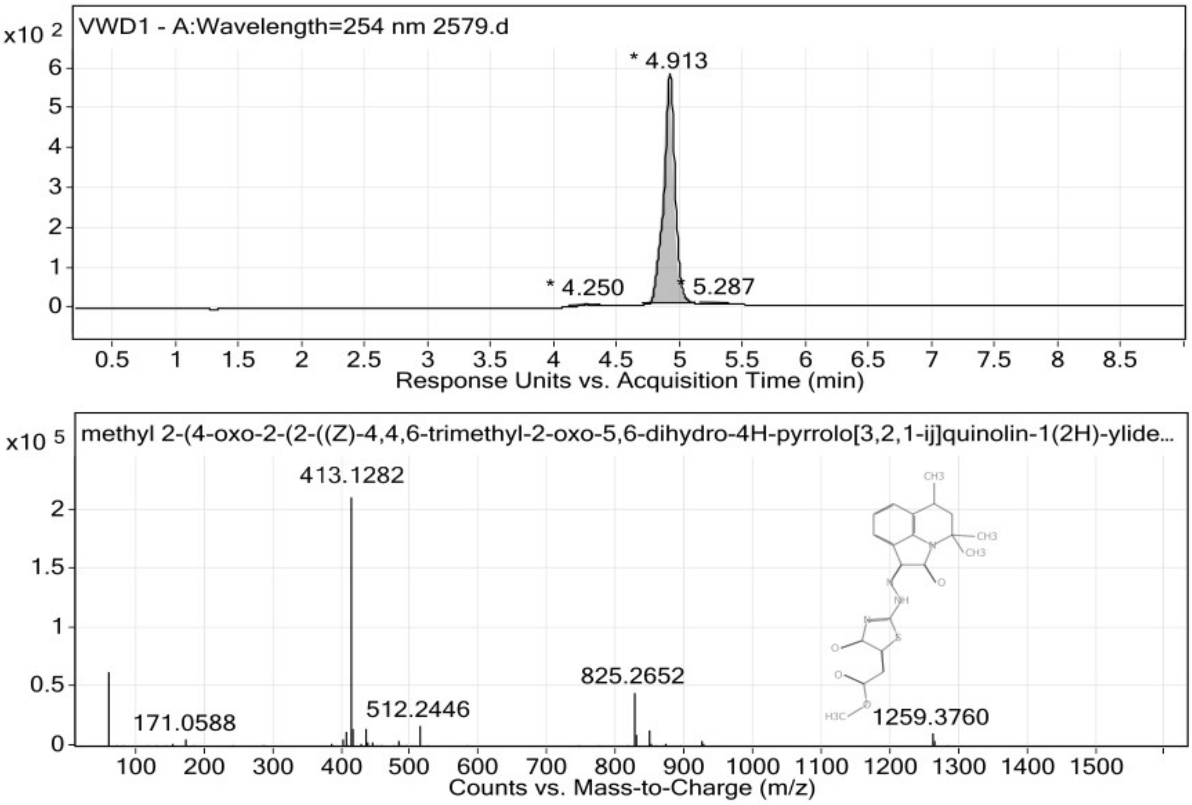

Figure S3. Data of HPLC-MS-ESI analysis of **3a**

^{1}H, ^{13}C NMR and data HPLC-HRMS-ESI spectra of methyl (Z)-2-(2-(2-((Z)-8-methoxy-4,4,6-trimethyl-2-oxo-5,6-dihydro-4*H*-pyrrolo[3,2,1-*ij*]quinolin-1(2*H*)-ylidene)hydrazineyl)-4-oxothiazol-5(4*H*)-ylidene)acetate 3b

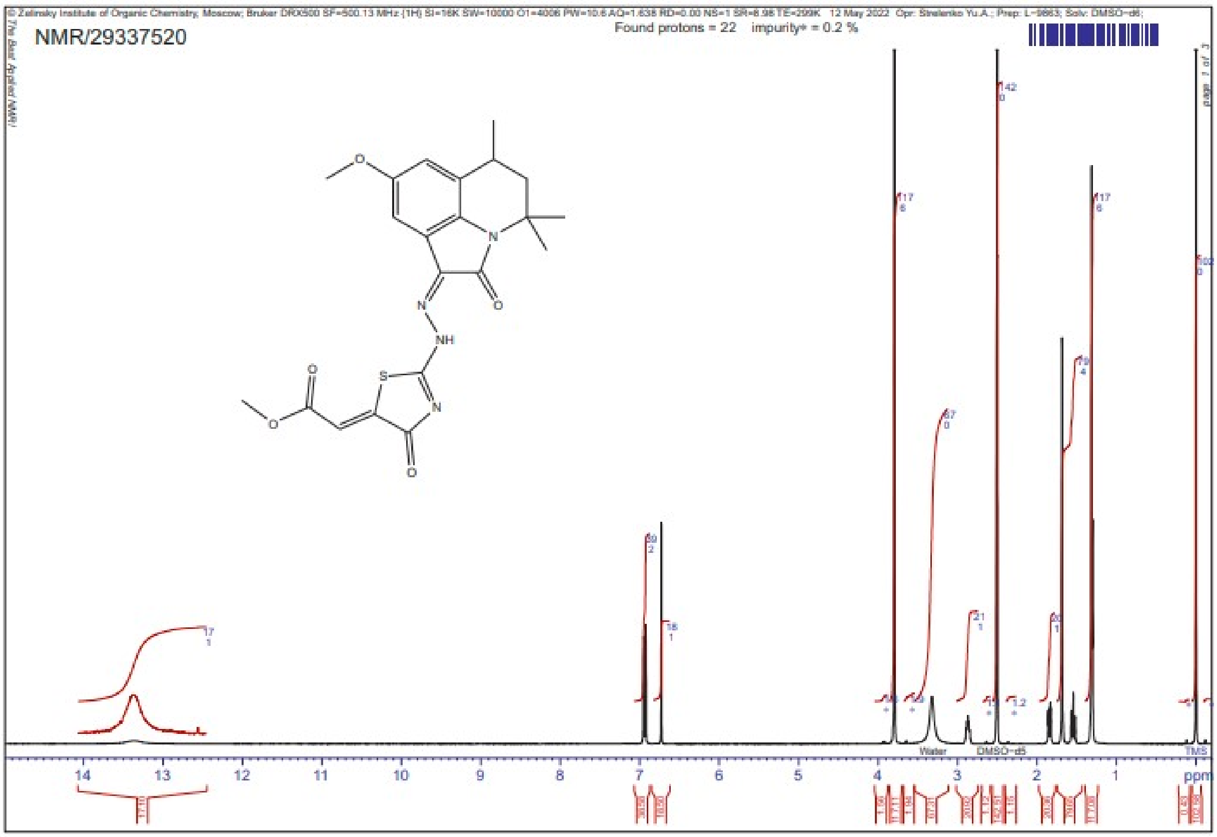

Figure S4. ^{1}H NMR spectrum of compound **3b**

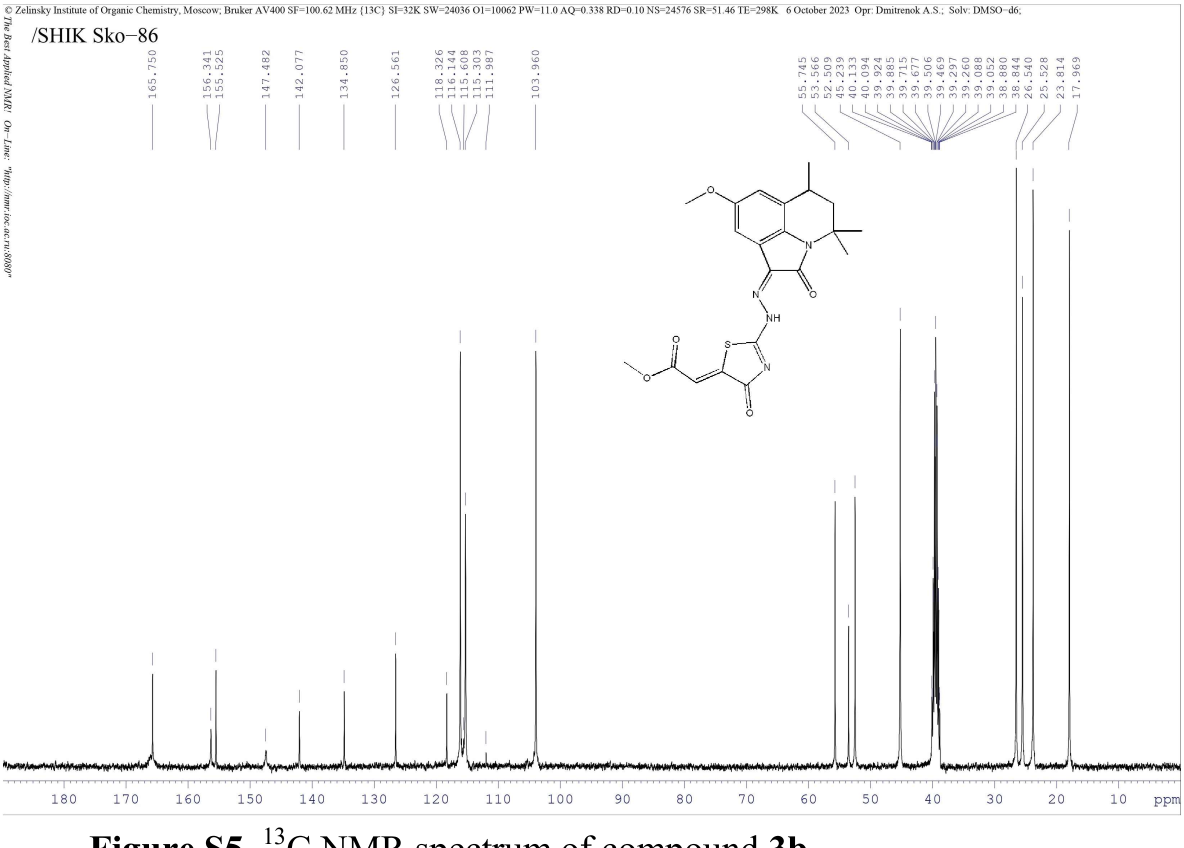

Figure S5. ^{13}C NMR spectrum of compound **3b**

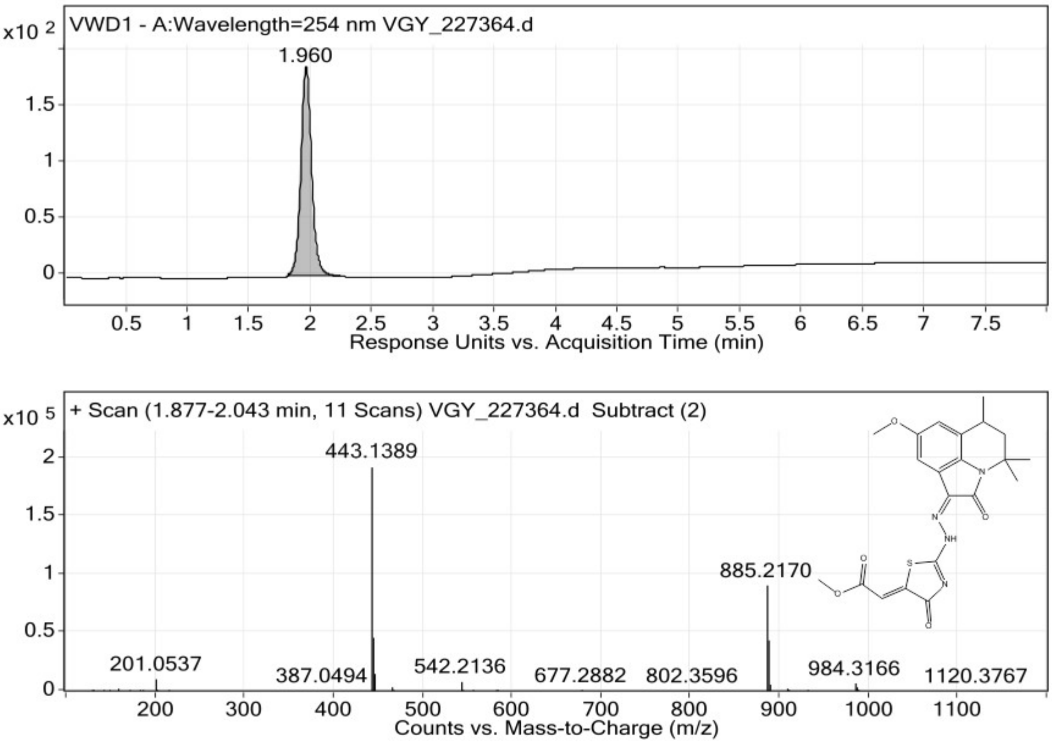

Figure S6. Data of HPLC-MS-ESI analysis of **3b**

^{1}H, ^{13}C NMR and data HPLC-HRMS-ESI spectra of methyl (Z)-2-(2-(2-((Z)-8-chloro-4,4,6-trimethyl-2-oxo-5,6-dihydro-4*H*-pyrrolo[3,2,1-*ij*]quinolin-1(2*H*)-ylidene)hydrazineyl)-4-oxothiazol-5(4*H*)-ylidene)acetate 3c

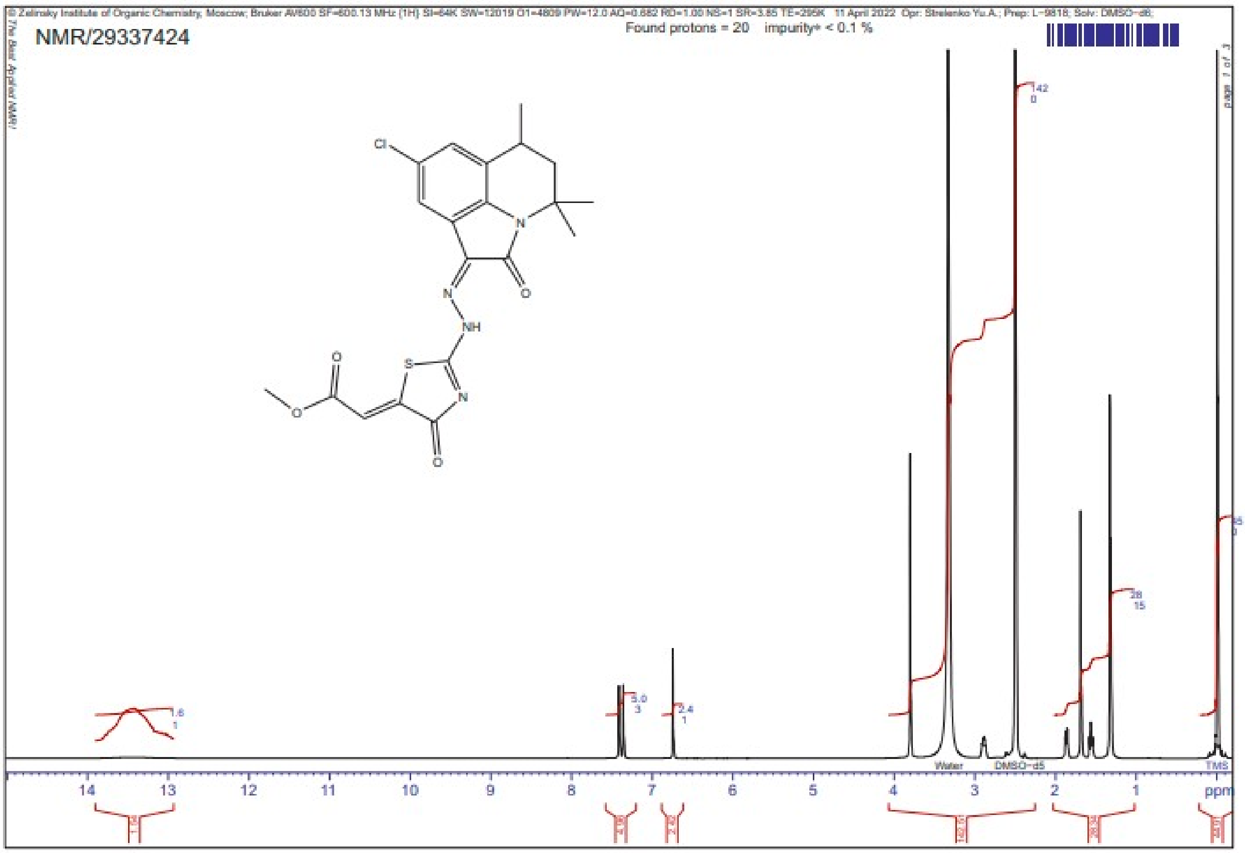

Figure S7. ^{1}H NMR spectrum of compound **3c**

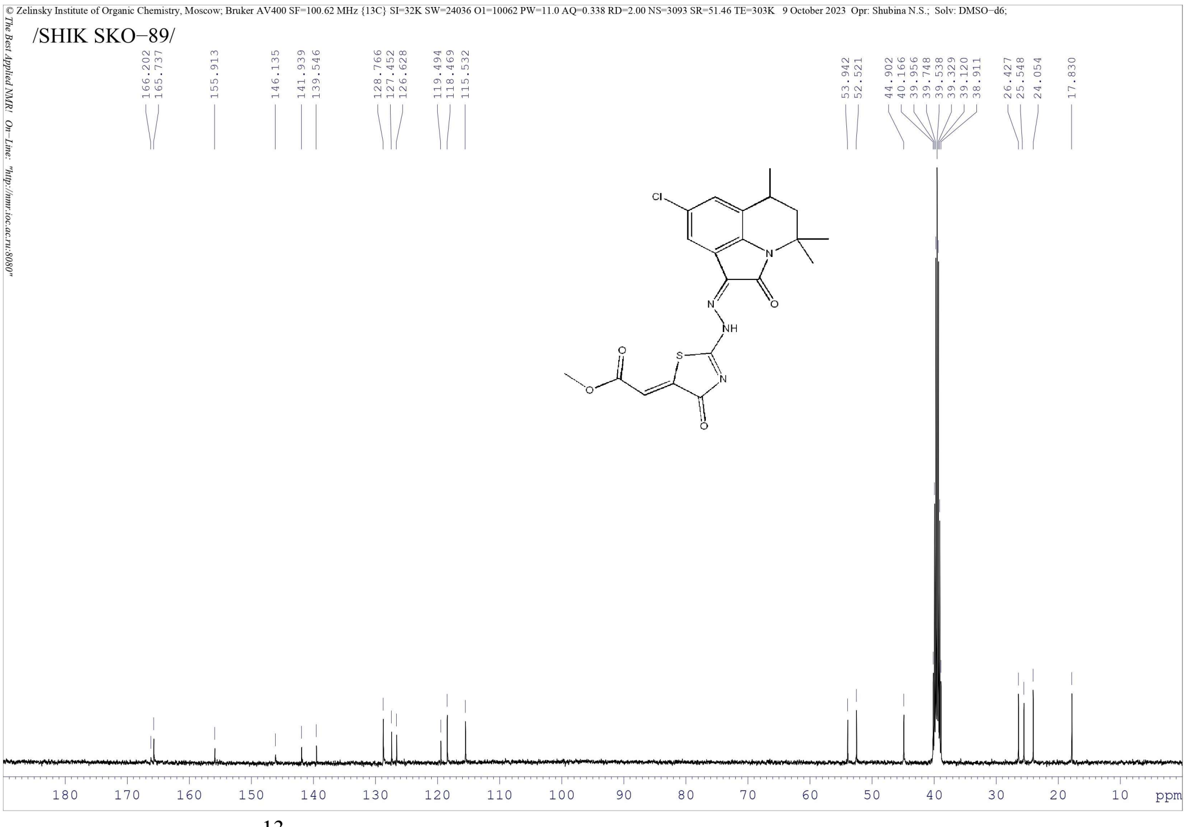

Figure S8. ^{13}C NMR spectrum of compound **3c**

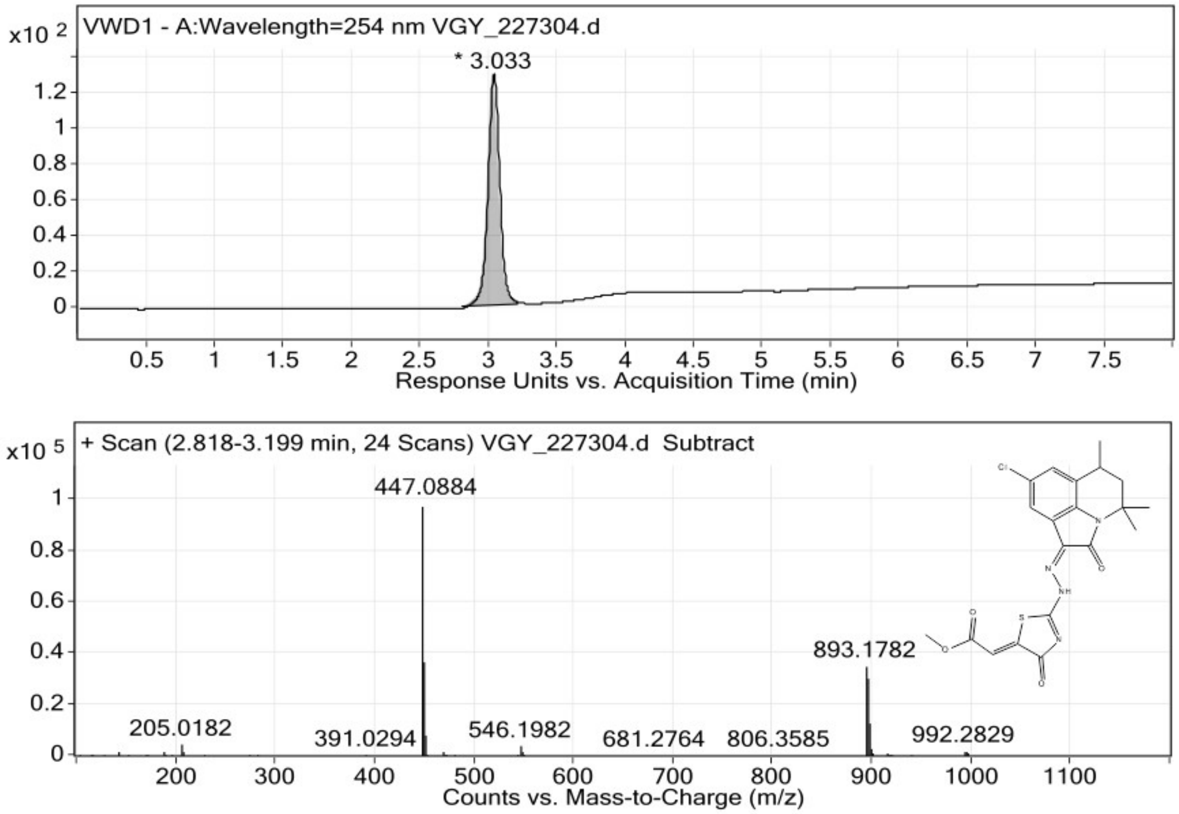

Figure S9. Data of HPLC-MS-ESI analysis of **3c**

^{1}H, ^{13}C NMR and data HPLC-HRMS-ESI spectra of methyl (Z)-2-(2-(2-((Z)-8-bromo-4,4,6-trimethyl-2-oxo-5,6-dihydro-4*H*-pyrrolo[3,2,1-*ij*]quinolin-1(2*H*)-ylidene)hydrazineyl)-4-oxothiazol-5(4*H*)-ylidene)acetate 3d

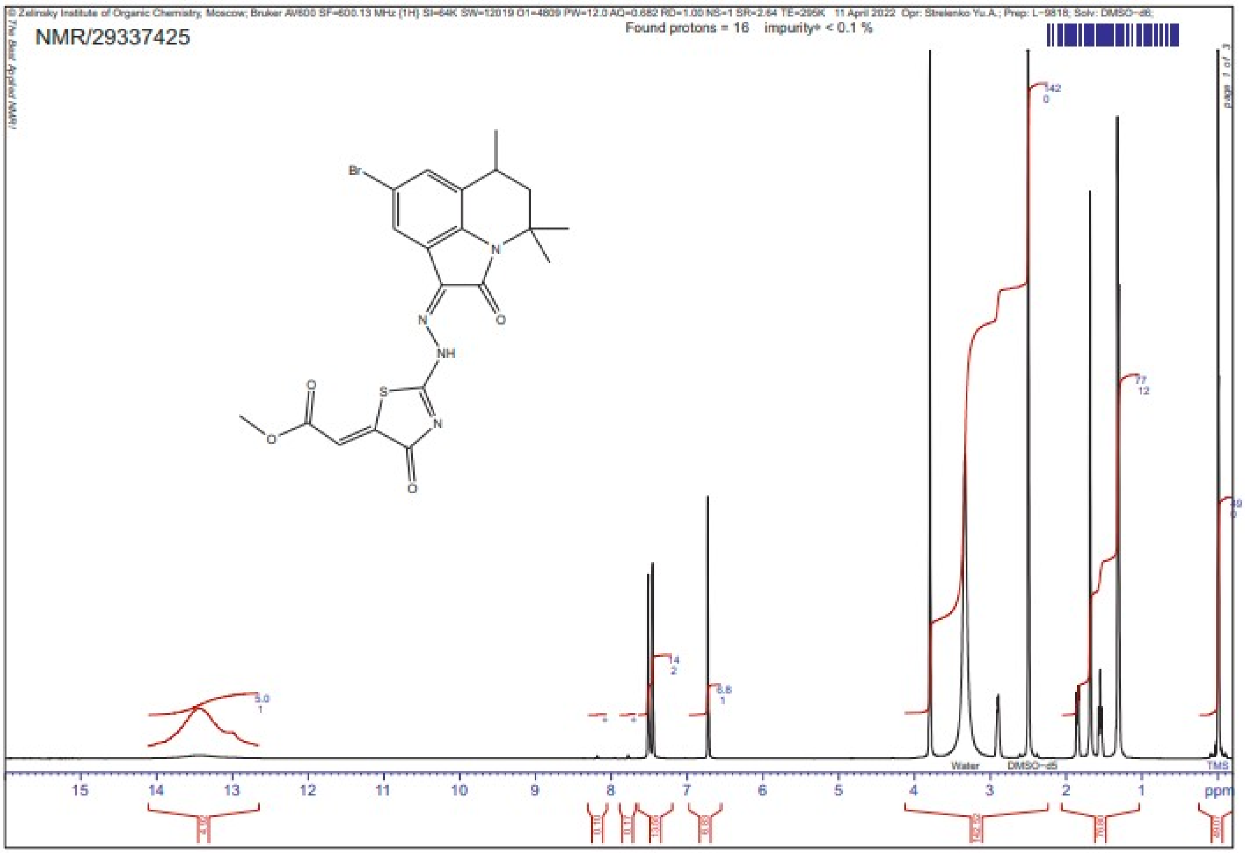

Figure S10. ^{1}H NMR spectrum of compound **3d**

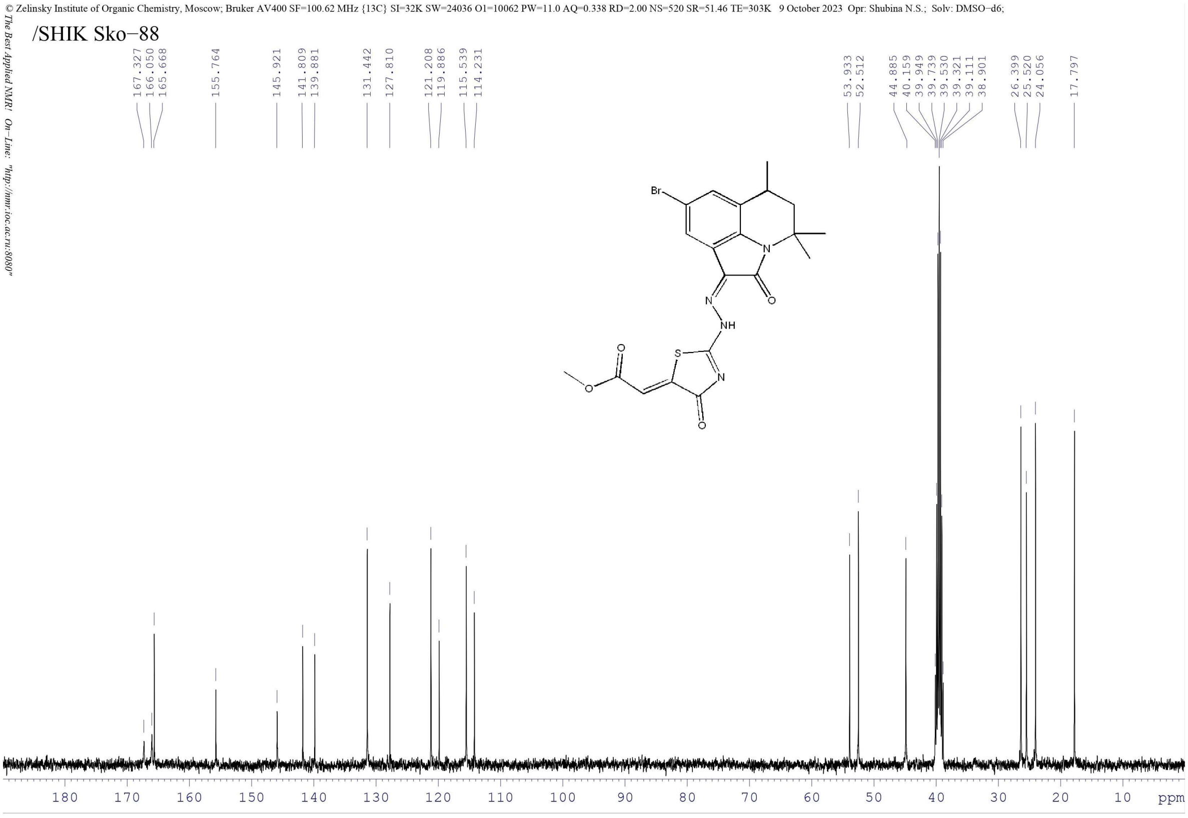

Figure S11. ^{13}C NMR spectrum of compound **3d**

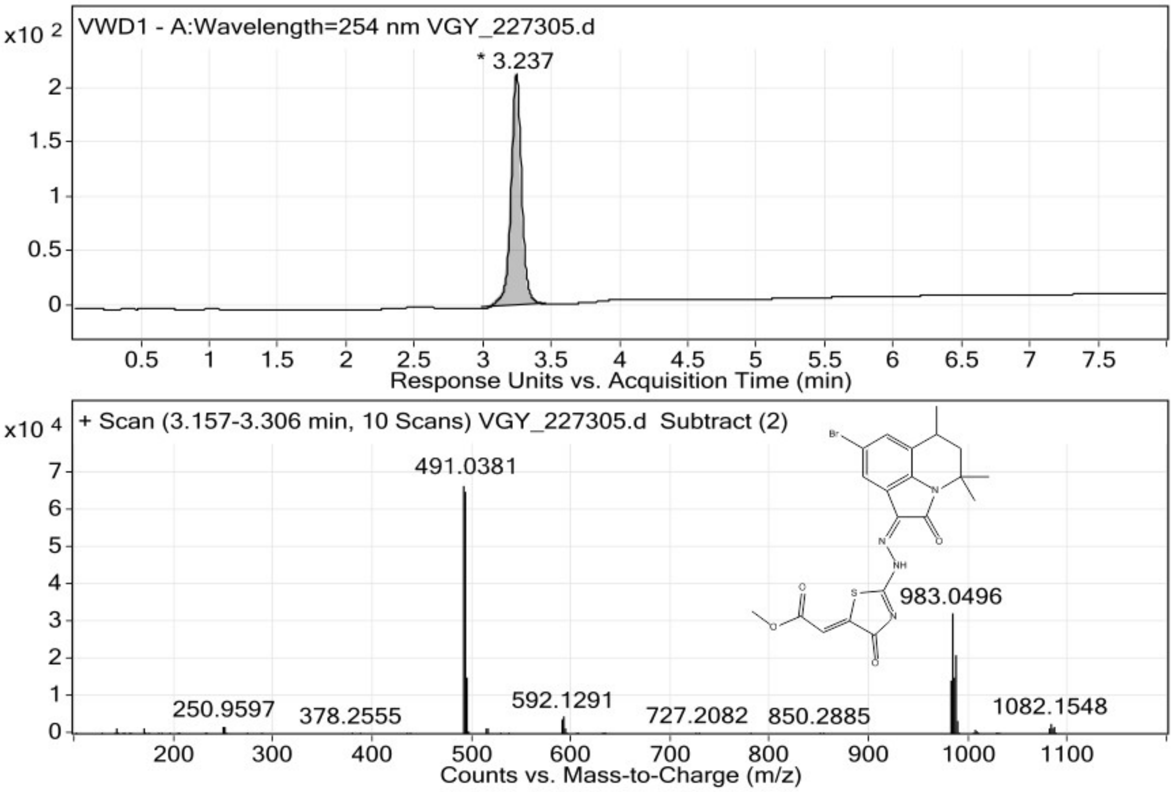

Figure S12. Data of HPLC-MS-ESI analysis of **3d**

^{1}H, ^{13}C NMR and data HPLC-HRMS-ESI spectra of methyl 2-(2-(2-(8-iodo-4,4,6-trimethyl-2-oxo-5,6-dihydro-4*H*-pyrrolo[3,2,1-*ij*]quinolin-1(2*H*)-ylidene)hydrazineyl)-4-oxothiazol-5(4*H*)-ylidene)acetate 3e

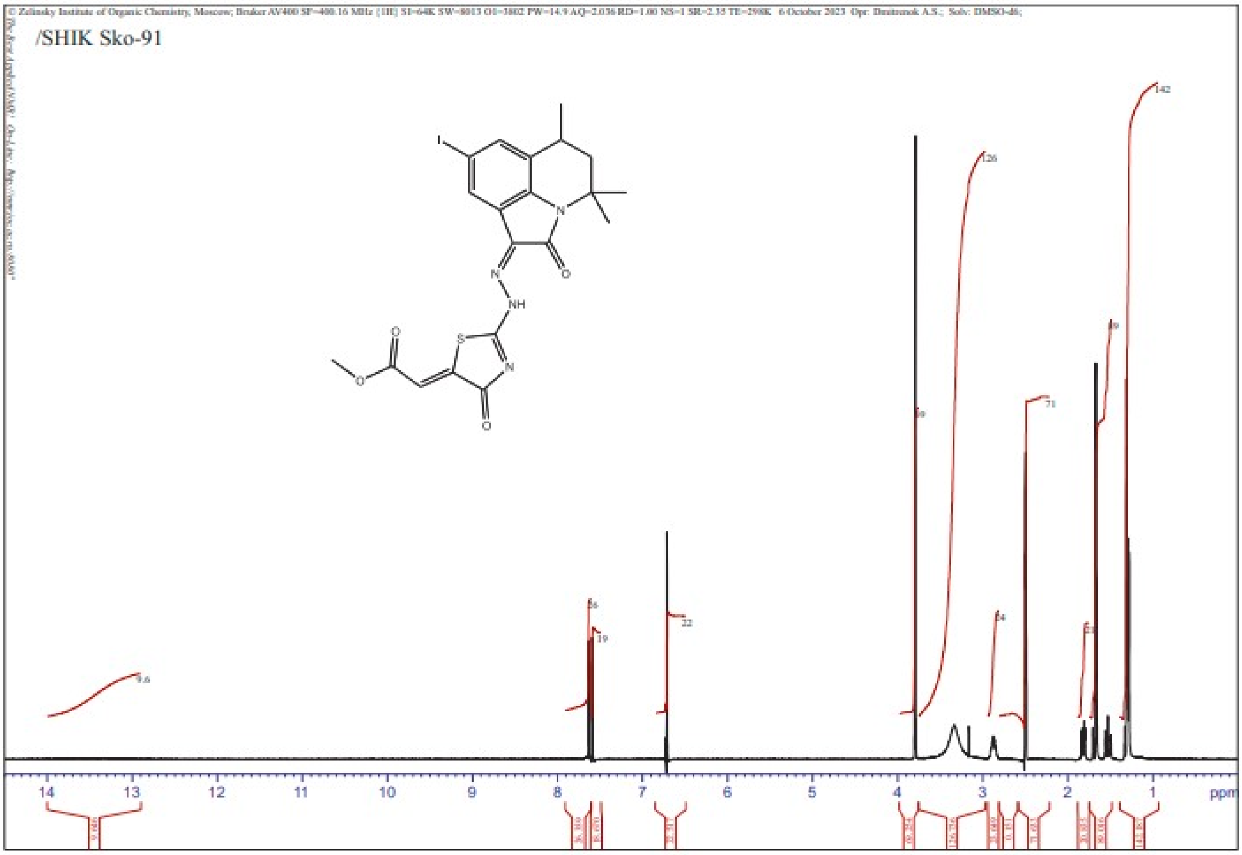

Figure S13. ^{1}H NMR spectrum of compound **3e**

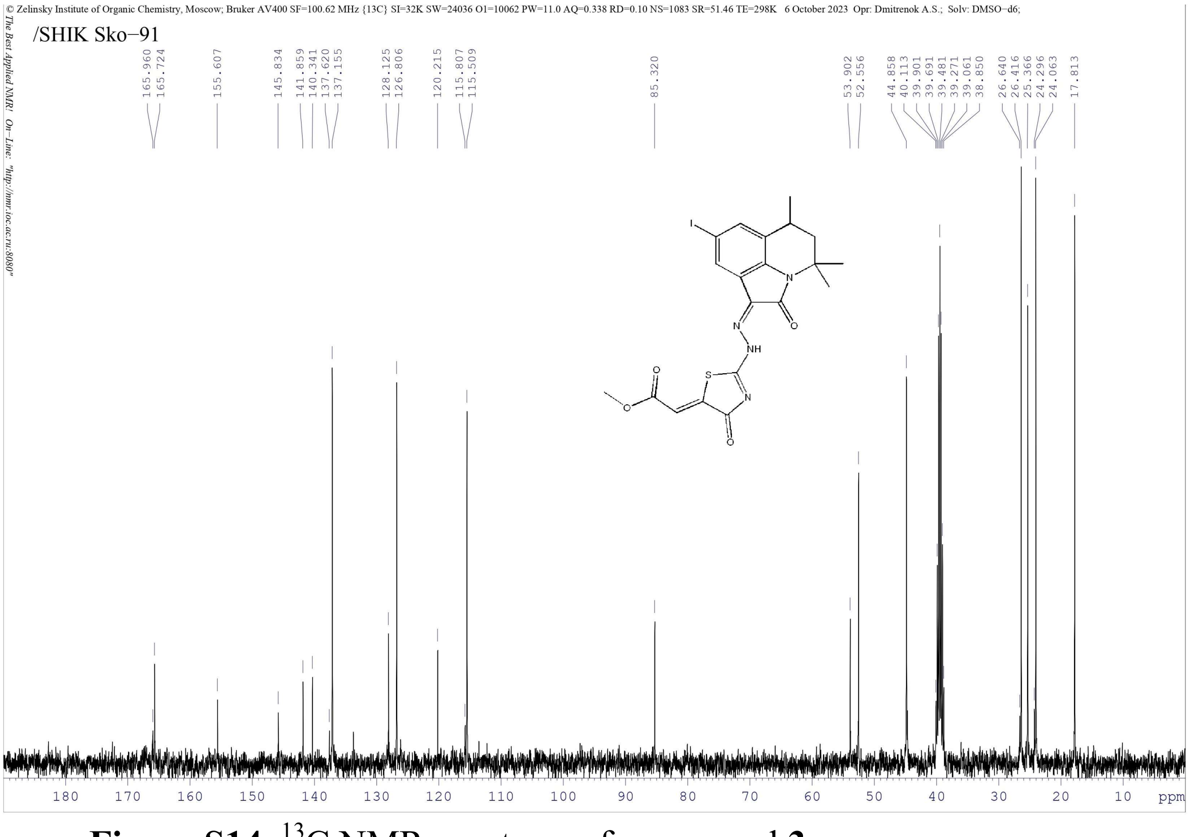

Figure S14. ^{13}C NMR spectrum of compound **3e**

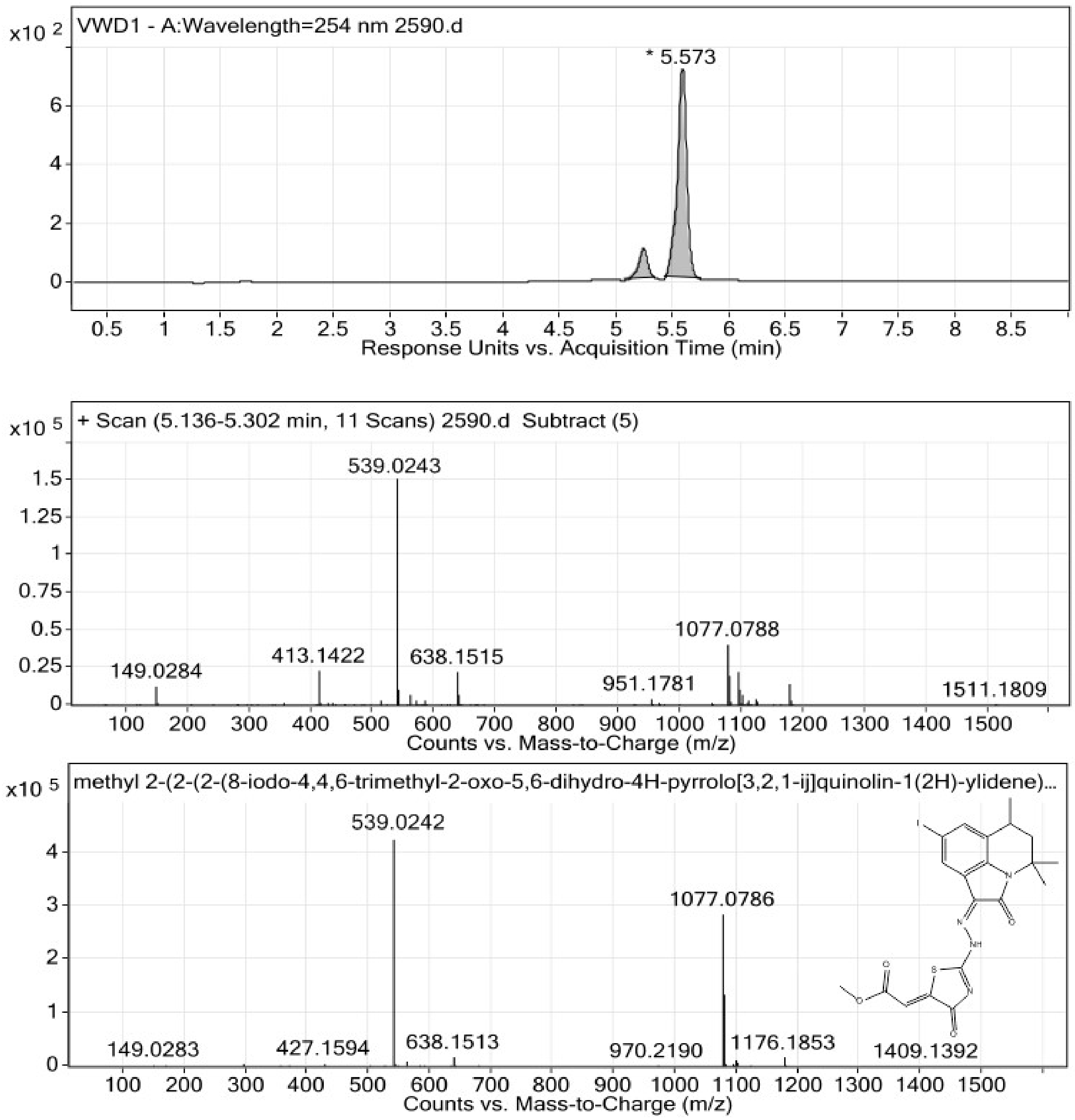

Figure S15. Data of HPLC-MS-ESI analysis of **3e**

^{1}H, ^{13}C NMR and data HPLC-HRMS-ESI spectra of methyl (Z)-2-(4-oxo-2-(2-((Z)-4,4,6,8,9-pentamethyl-2-oxo-5,6-dihydro-4*H*-pyrrolo[3,2,1-*ij*]quinolin-1(2*H*)-ylidene)hydrazineyl)thiazol-5(4*H*)-ylidene)acetate 3f

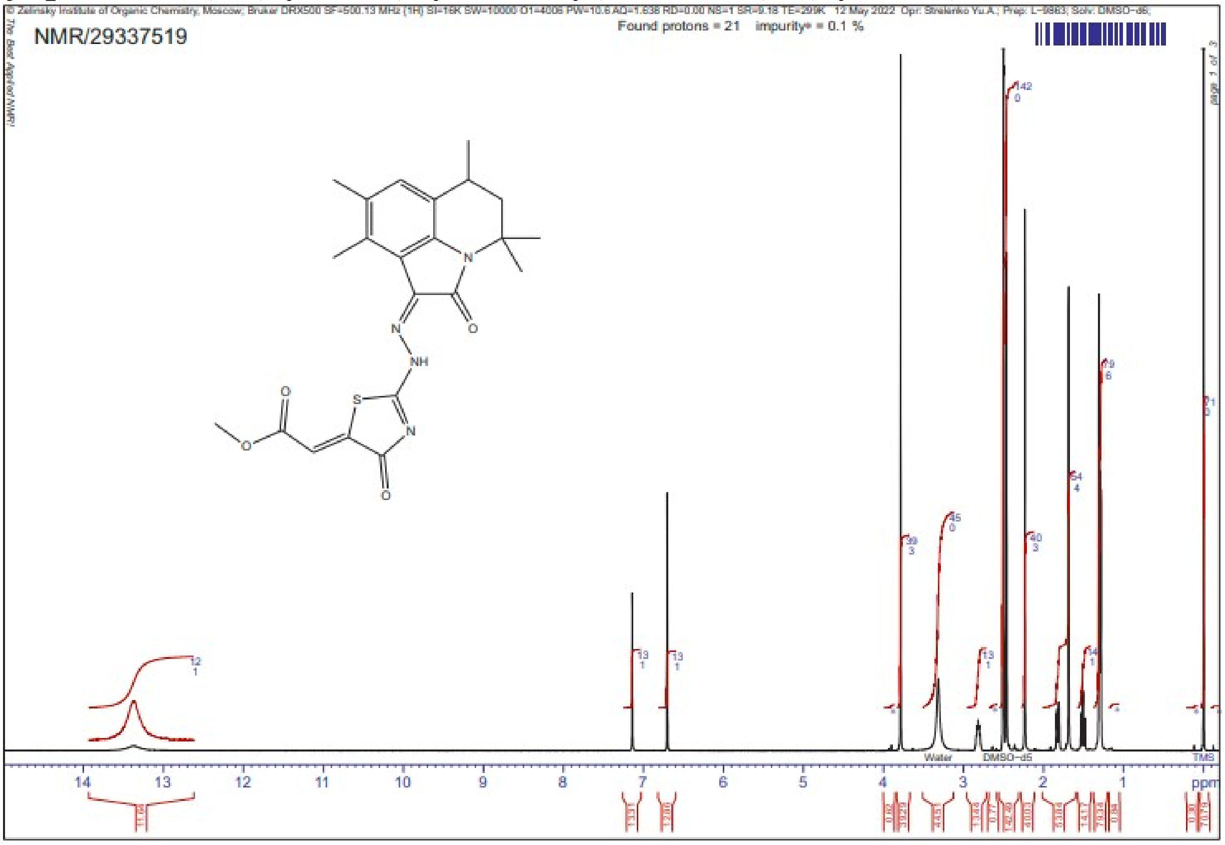

Figure S16. ^{1}H NMR spectrum of compound **3f**

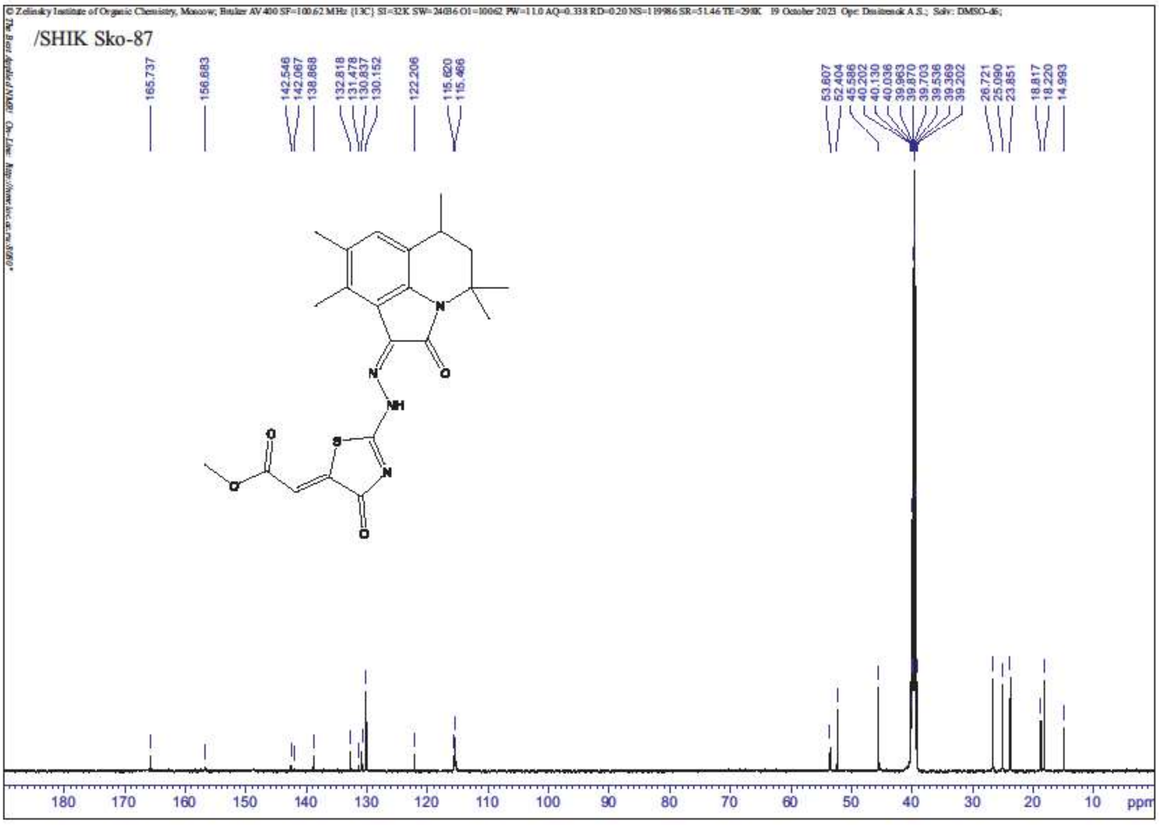

Figure S17. ^{13}C NMR spectrum of compound **3f**

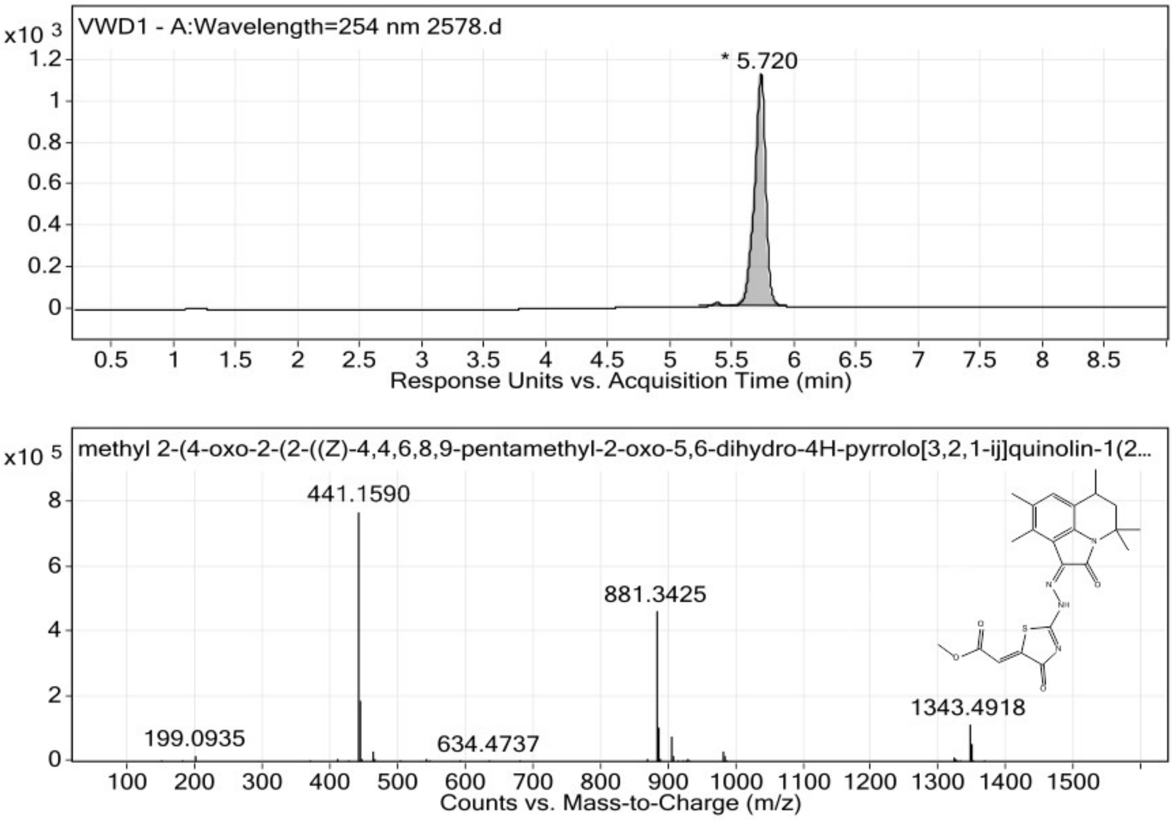

Figure S18. Data of HPLC-MS-ESI analysis of **3f**

^{1}H, ^{13}C NMR and data HPLC-HRMS-ESI spectra of methyl (Z)-2-(2-(2-(8-chloro-4,4,6,9-tetramethyl-2-oxo-5,6-dihydro-4*H*-pyrrolo[3,2,1-*ij*]quinolin-1(2*H*)-ylidene)hydrazineyl)-4-oxothiazol-5(4*H*)-ylidene)acetate 3g

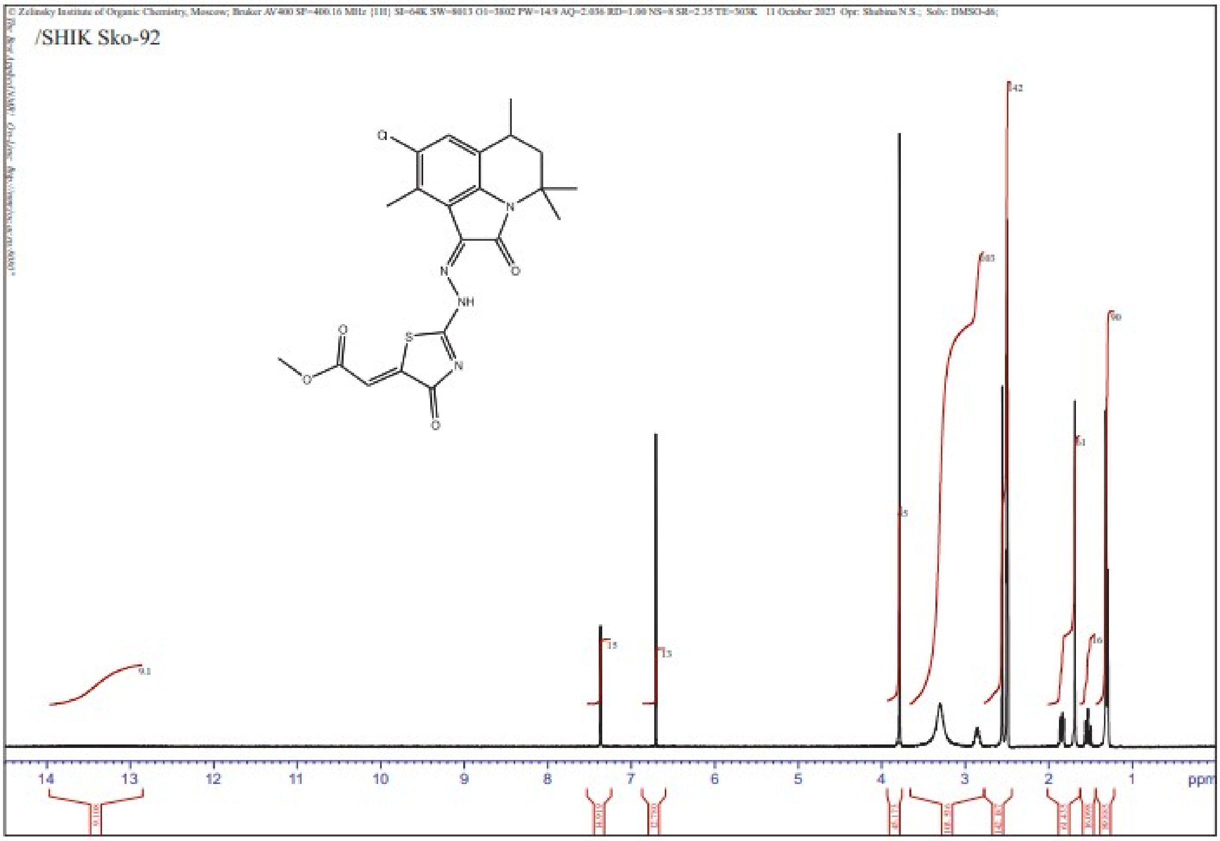

Figure S19. ^{1}H NMR spectrum of compound **3g**

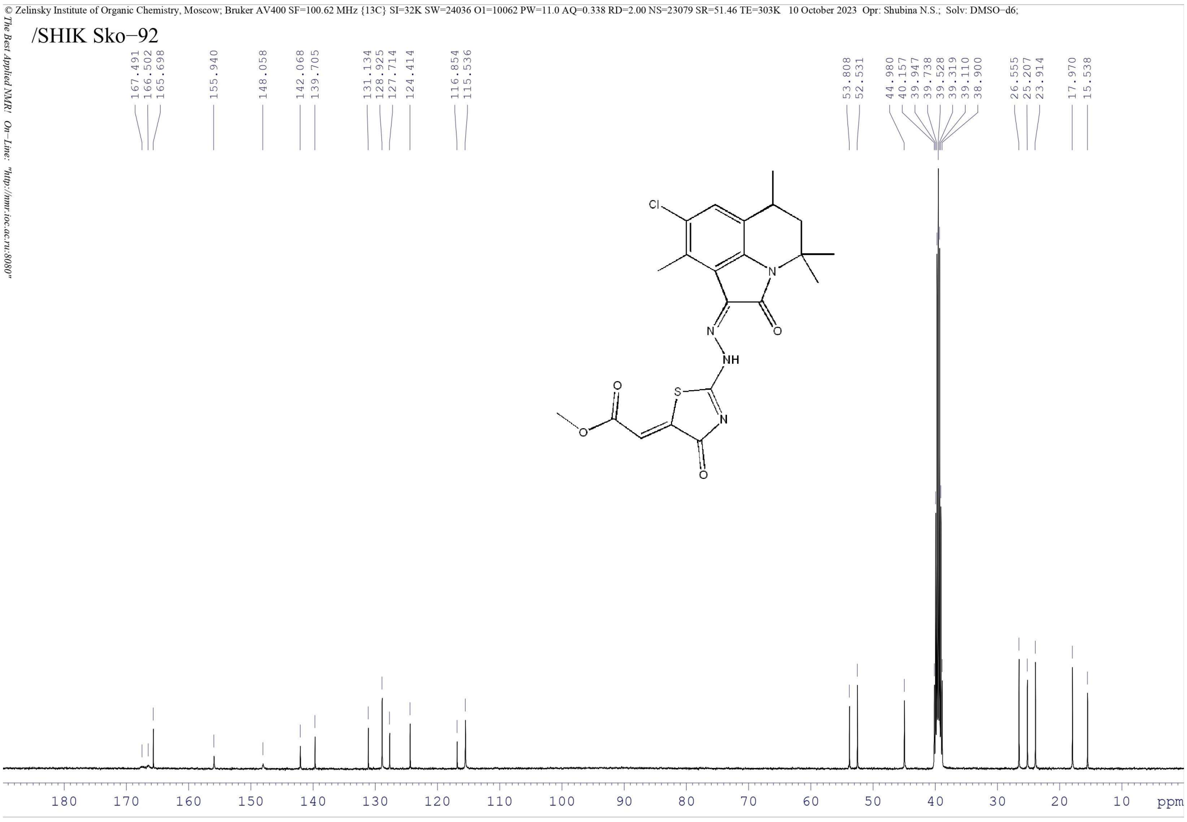

Figure S20. ^{13}C NMR spectrum of compound **3g**

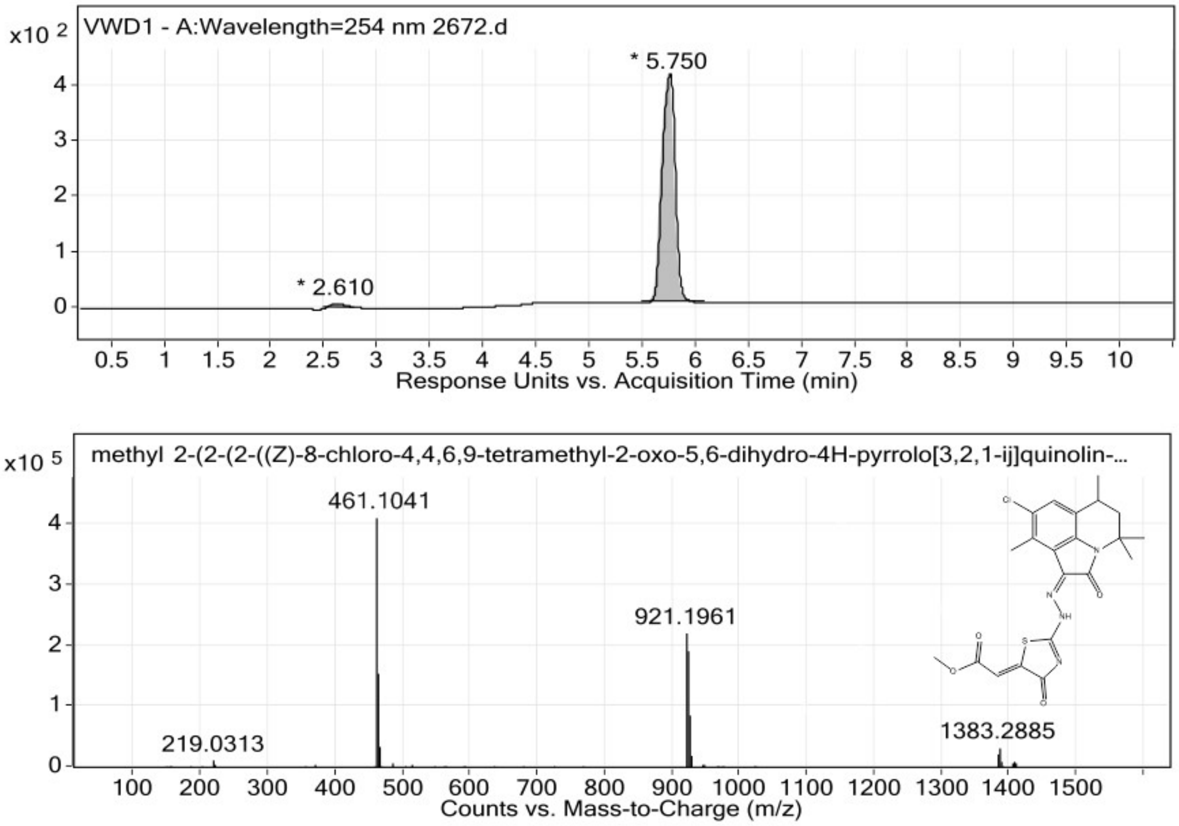

Figure S21. Data of HPLC-MS-ESI analysis of **3g**

^{1}H, ^{13}C NMR and data HPLC-HRMS-ESI spectra of methyl (Z)-(2-(2-(2-(8-bromo-4,4,6,9-tetramethyl-2-oxo-5,6-dihydro-4*H*-pyrrolo[3,2,1-*ij*]quinolin-1(2*H*)-ylidene)hydrazineyl)-4-oxothiazol-5(4*H*)-ylidene)acetate 3h

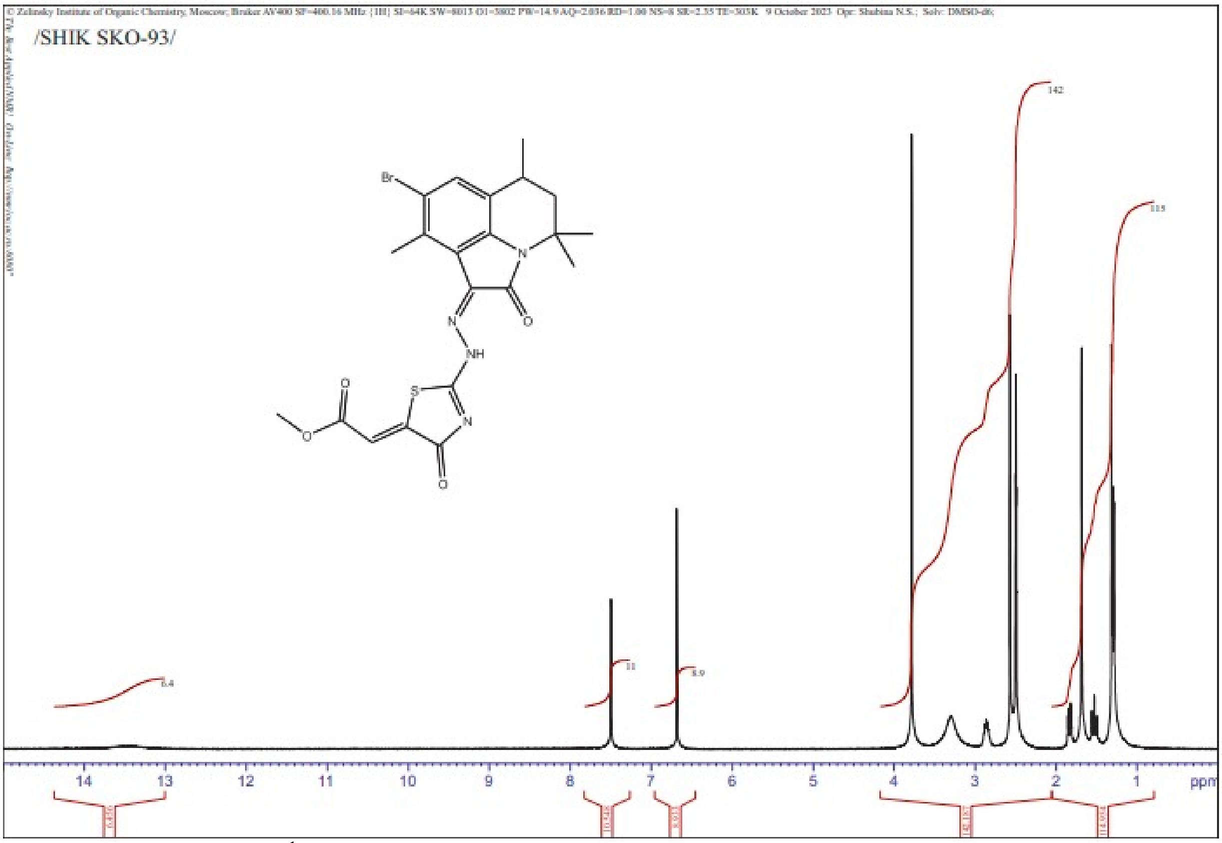

Figure S22. ^{1}H NMR spectrum of compound **3h**

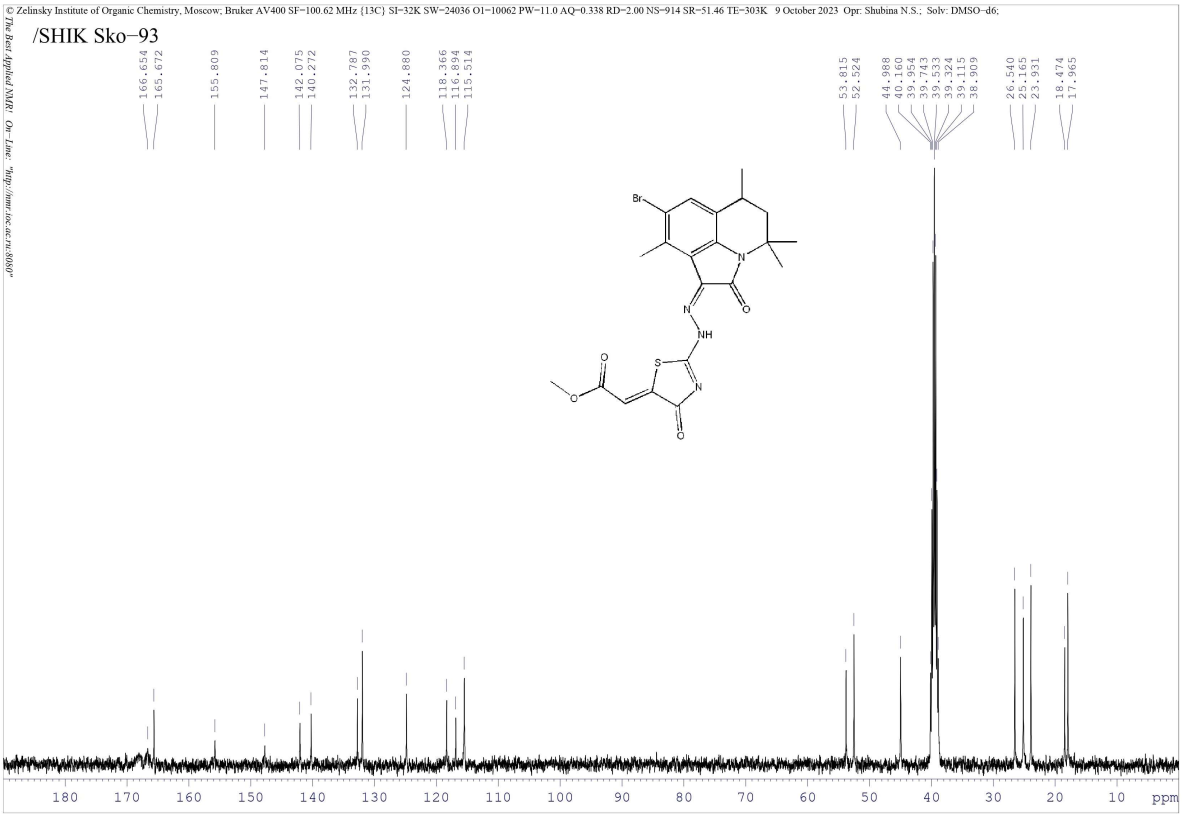

Figure S23. ^{13}C NMR spectrum of compound **3h**

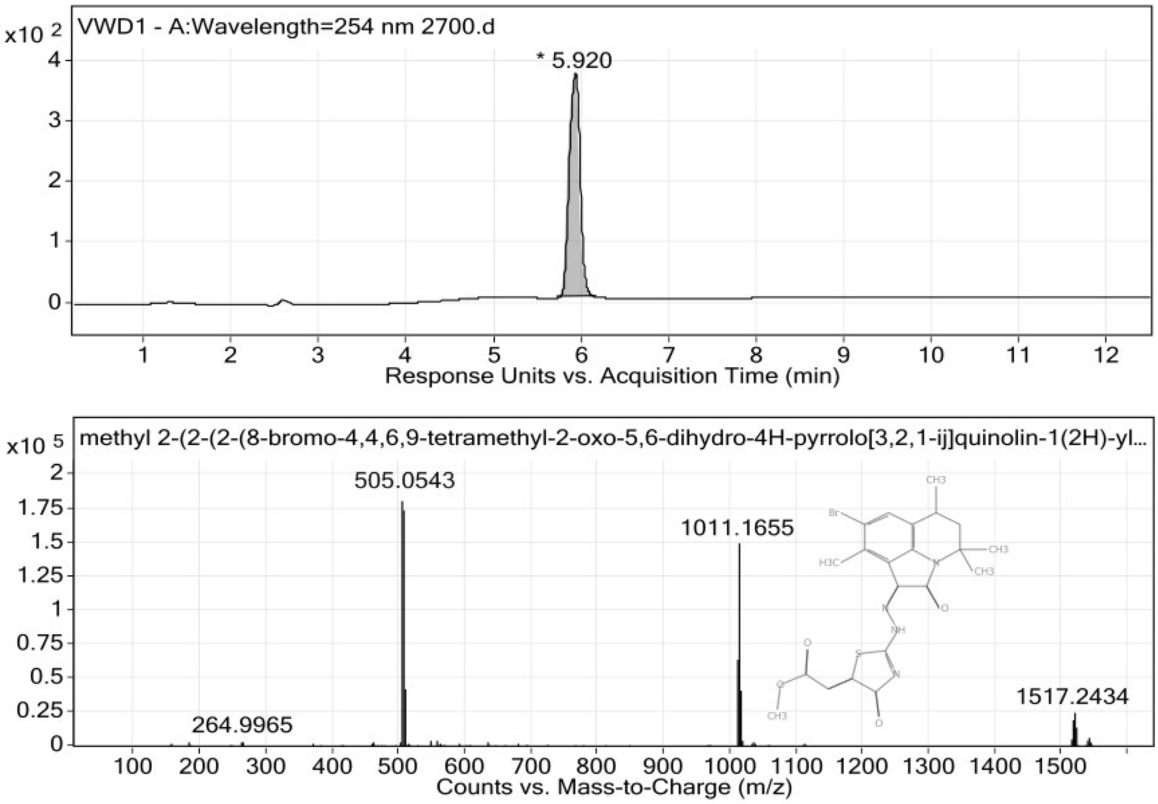

Figure S24. Data of HPLC-MS-ESI analysis of **3h**

^{1}H, ^{13}C NMR and data HPLC-HRMS-ESI spectra of methyl (Z)-2-(2-(2-((Z)-6-(4-chlorophenyl)-4,4,6-trimethyl-2-oxo-5,6-dihydro-4*H*-pyrrolo[3,2,1-*ij*]quinolin-1(2*H*)-ylidene)hydrazineyl)-4-oxothiazol-5(4*H*)-ylidene)acetate 3i

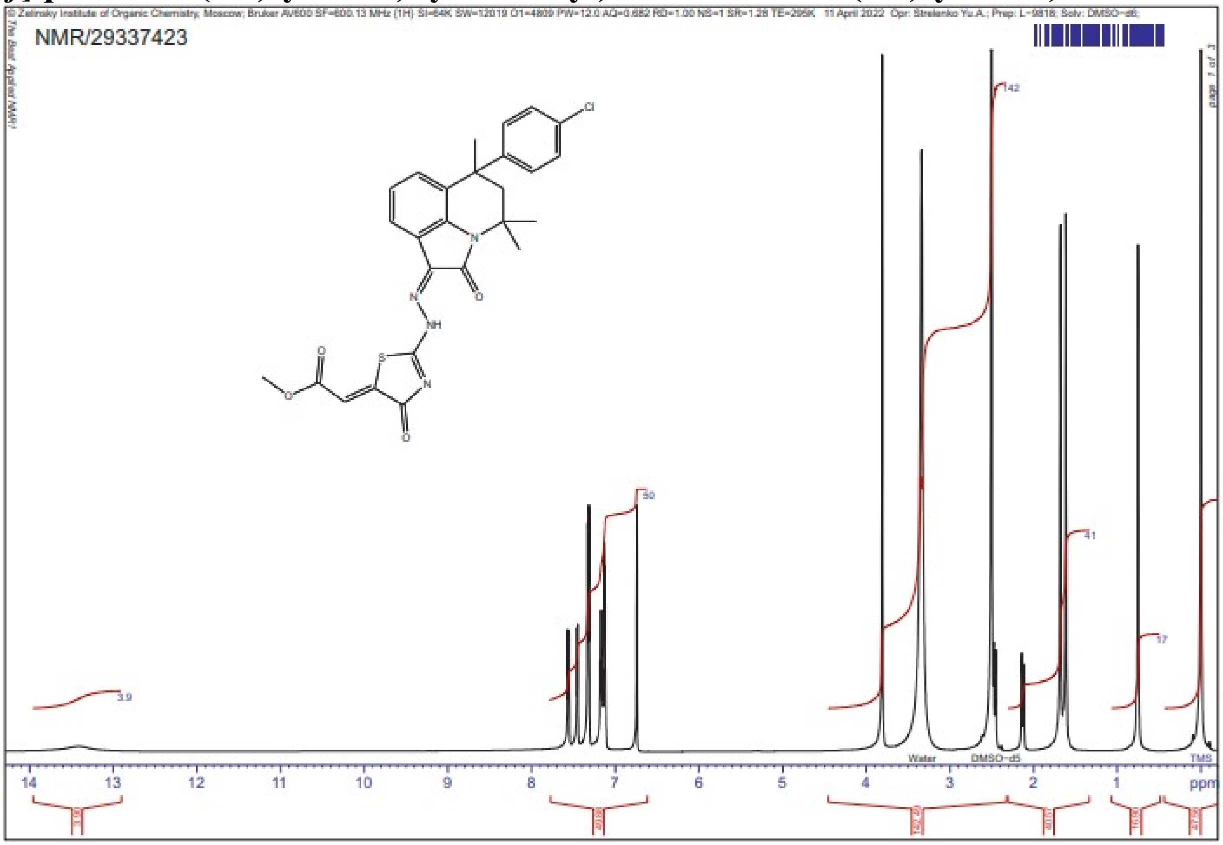

Figure S25. ^{1}H NMR spectrum of compound **3i**

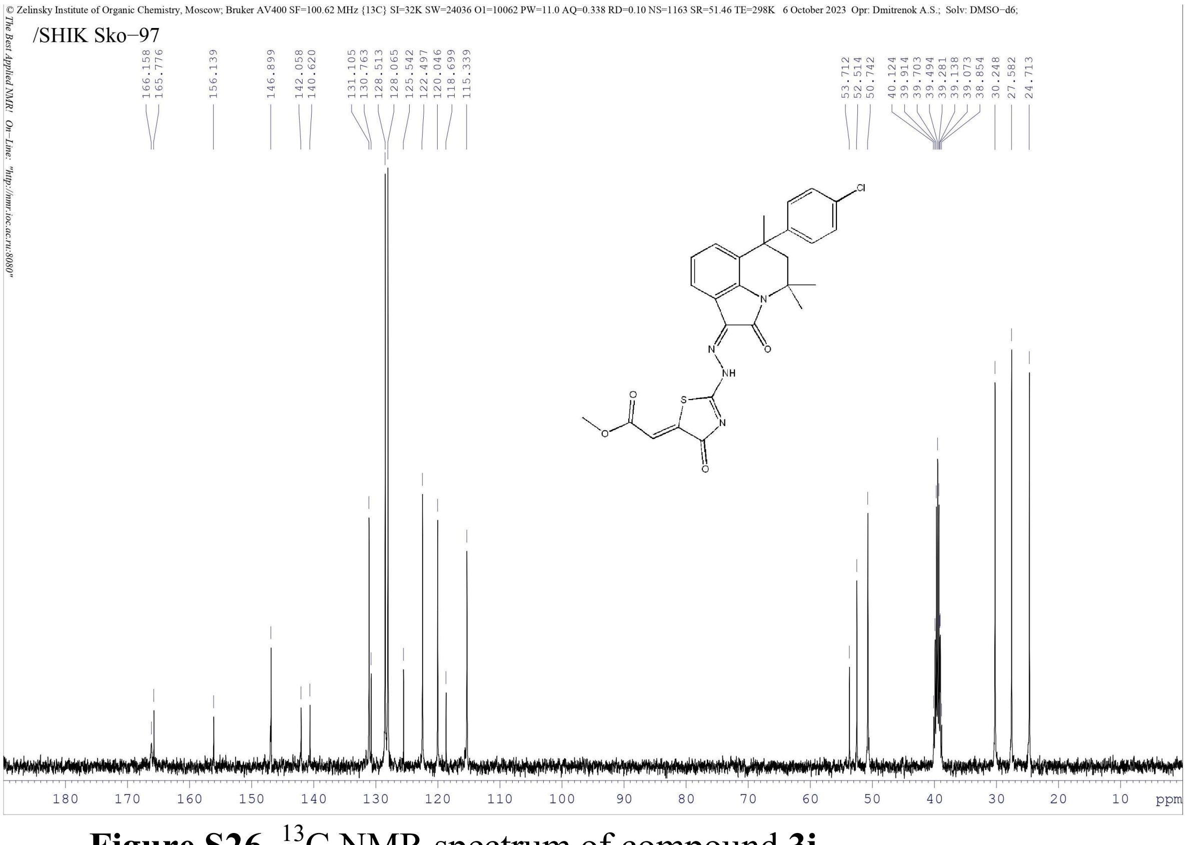

Figure S26. ^{13}C NMR spectrum of compound **3i**

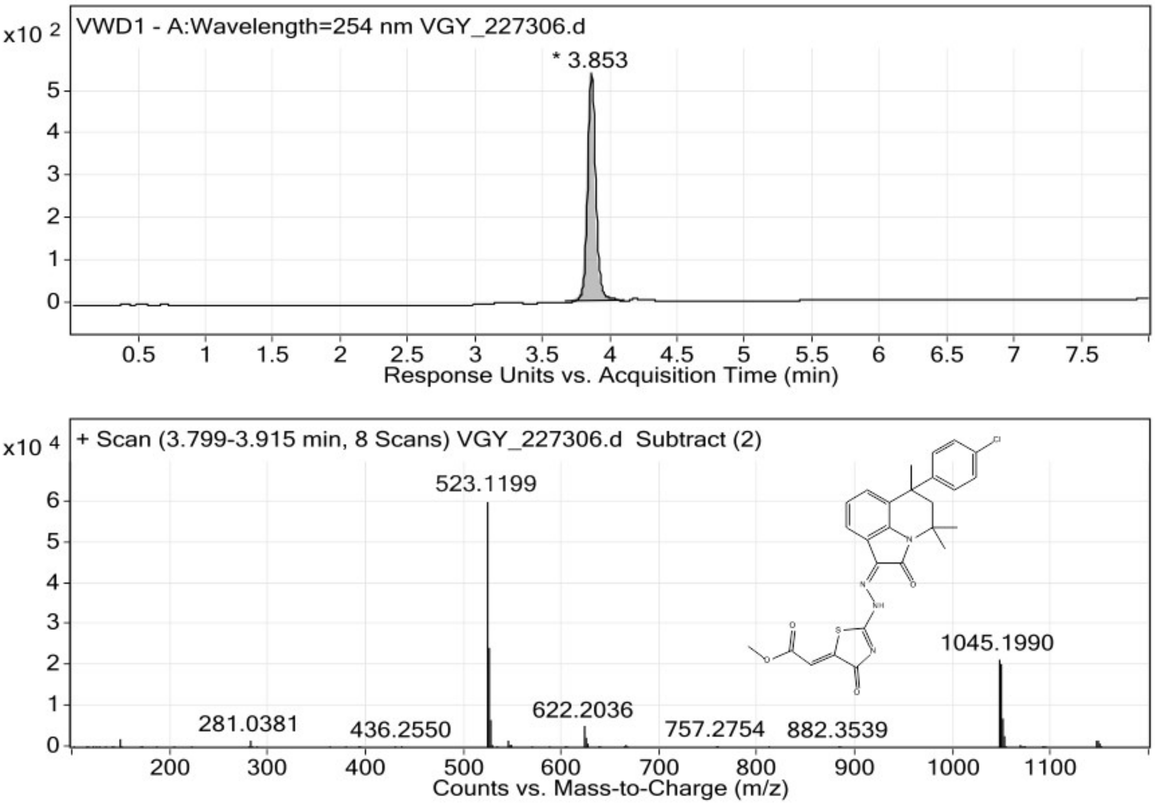

Figure S27. Data of HPLC-MS-ESI analysis of **3i**

^{1}H, ^{13}C NMR and data HPLC-HRMS-ESI spectra of methyl 2-(2-(2-(6-(4-chlorophenyl)-4,4,6,8-tetramethyl-2-oxo-5,6-dihydro-4*H*-pyrrolo[3,2,1-*ij*]quinolin-1(2*H*)-ylidene)hydrazineyl)-4-oxothiazol-5(4*H*)-ylidene)acetate 3j

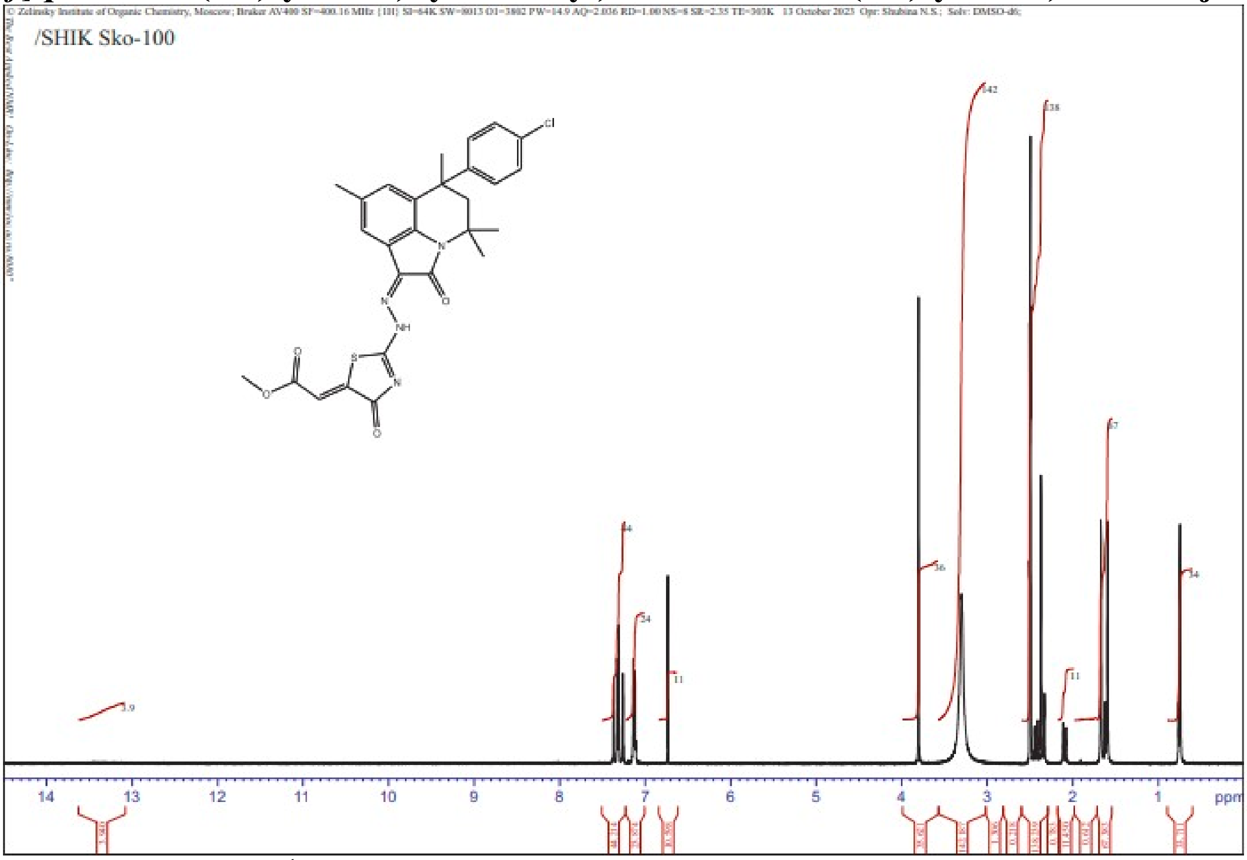

Figure S28. ^{1}H NMR spectrum of compound **3j**

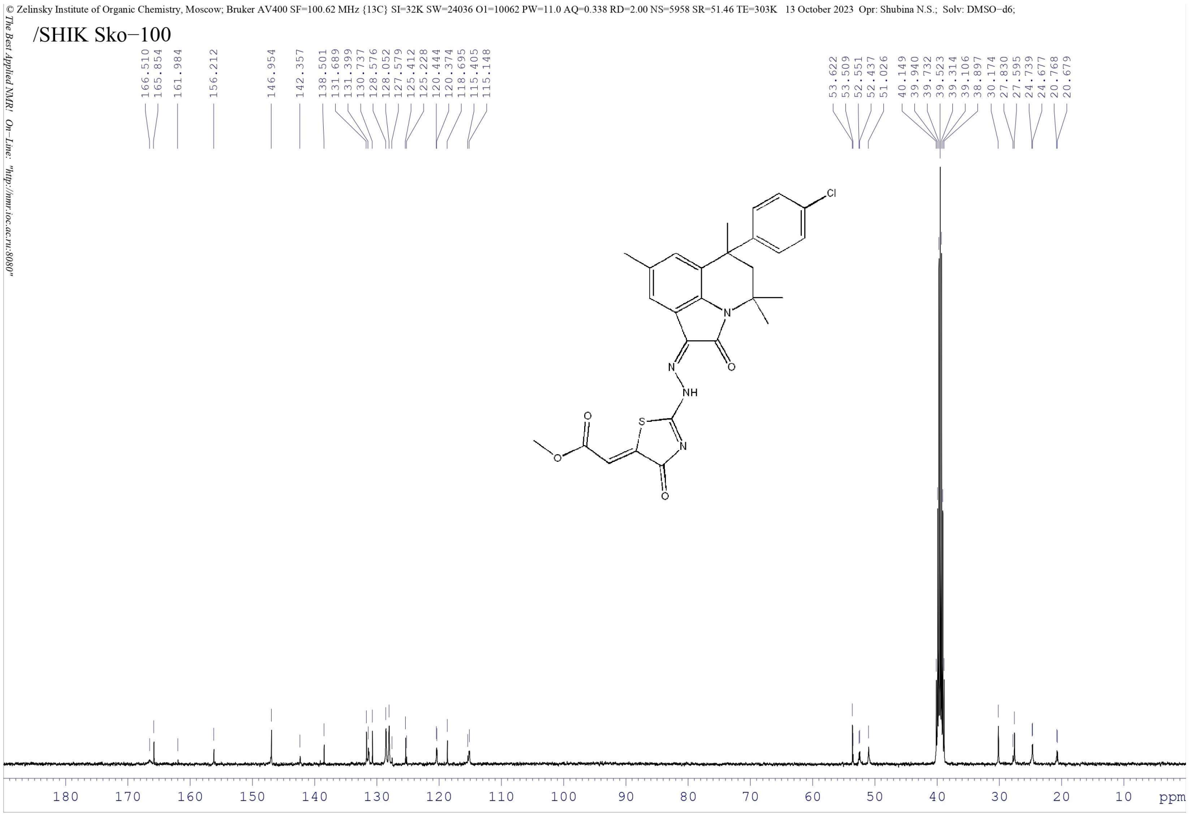

Figure S29. ^{13}C NMR spectrum of compound **3j**

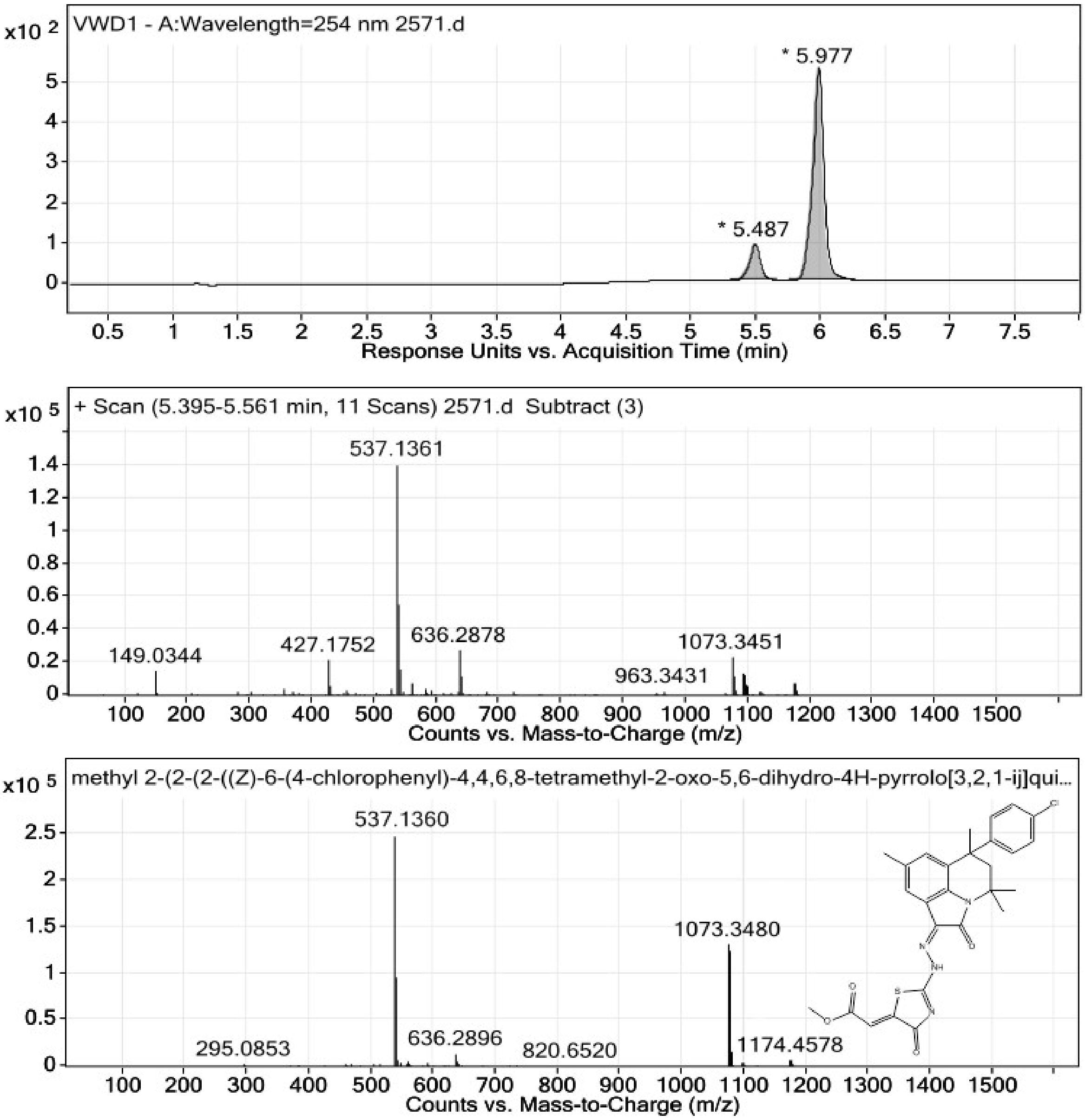

Figure S30. Data of HPLC-MS-ESI analysis of **3j**

^{1}H, ^{13}C NMR and data HPLC-HRMS-ESI spectra of methyl (Z)-2-(2-(2-((Z)-8-chloro-6-(4-chlorophenyl)-4,4,6-trimethyl-2-oxo-5,6-dihydro-4*H*-pyrrolo[3,2,1-*ij*]quinolin-1(2*H*)-ylidene)hydrazineyl)-4-oxothiazol-5(4*H*)-ylidene)acetate 3k

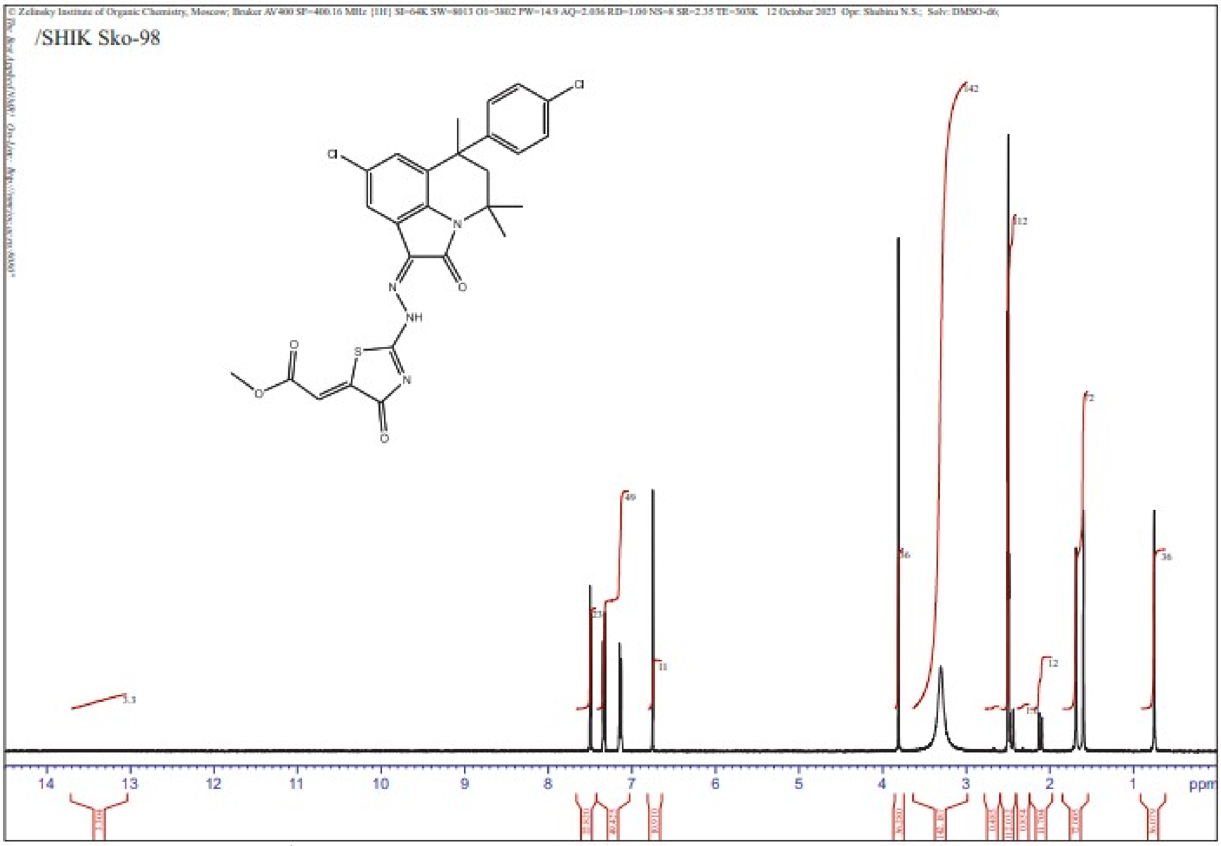

Figure S31. ^{1}H NMR spectrum of compound **3k**

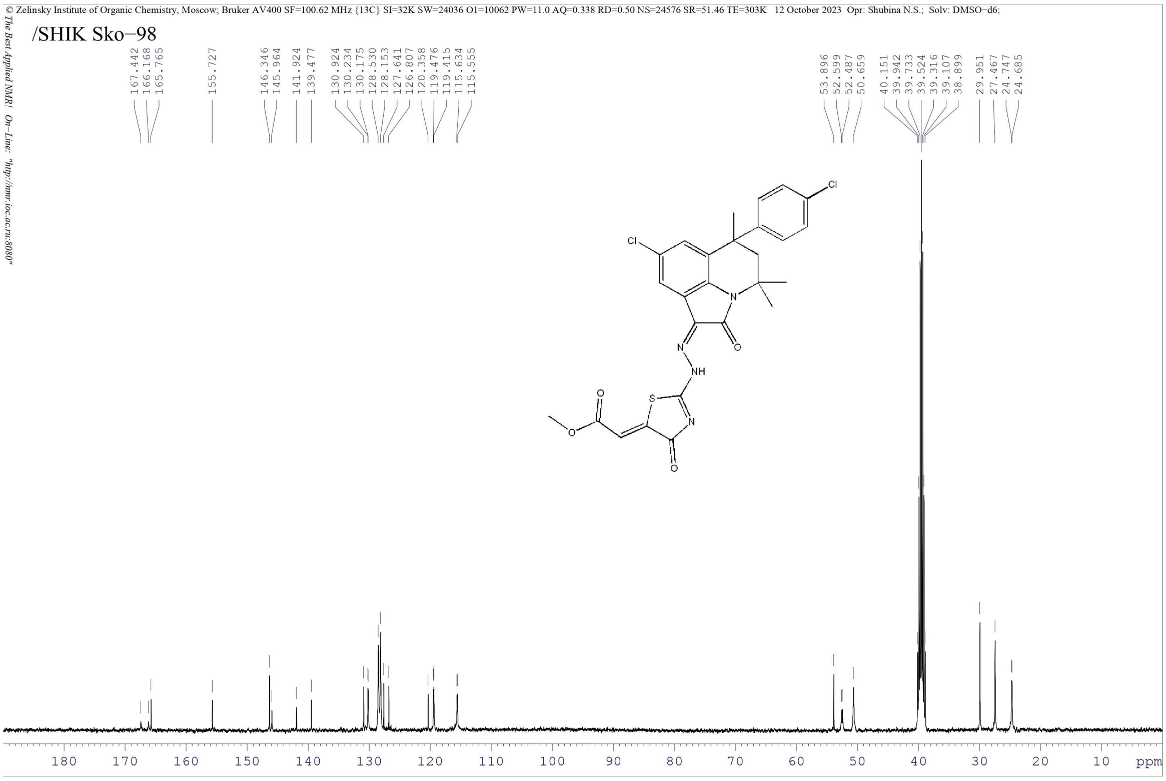

Figure S32. ^{13}C NMR spectrum of compound **3k**

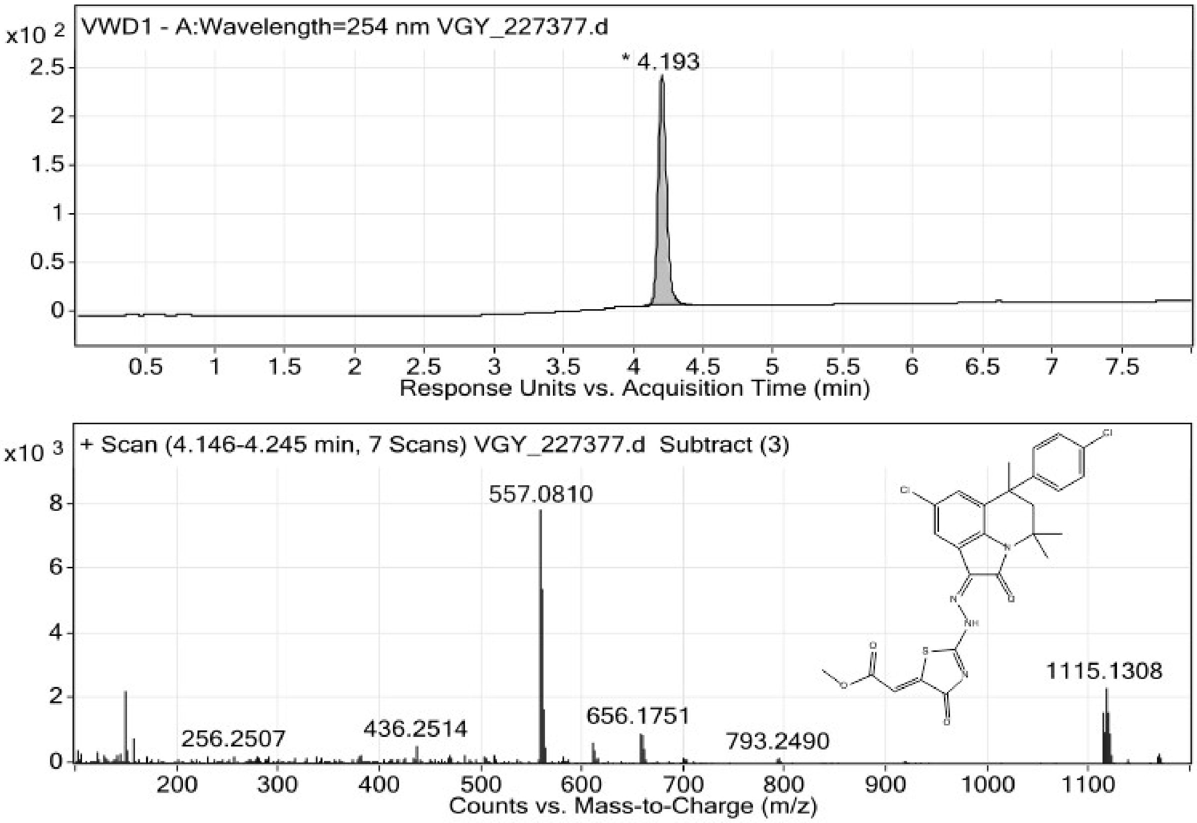

Figure S33. Data of HPLC-MS-ESI analysis of **3k**

^{1}H, ^{13}C NMR and data HPLC-HRMS-ESI spectra of methyl (Z)-2-(2-(2-((Z)-8-bromo-6-(4-chlorophenyl)-4,4,6-trimethyl-2-oxo-5,6-dihydro-4*H*-pyrrolo[3,2,1-*ij*]quinolin-1(2*H*)-ylidene)hydrazineyl)-4-oxothiazol-5(4*H*)-ylidene)acetate 3l

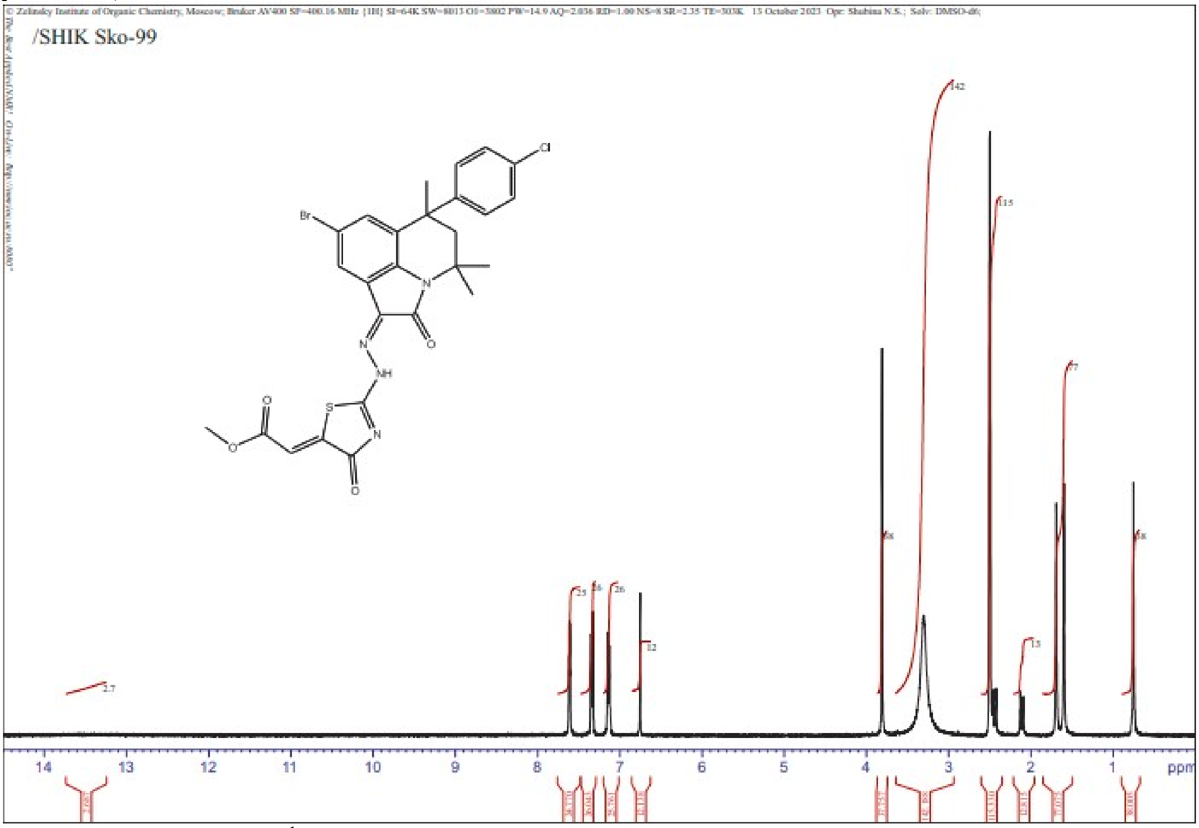

Figure S34. ^{1}H NMR spectrum of compound **3l**

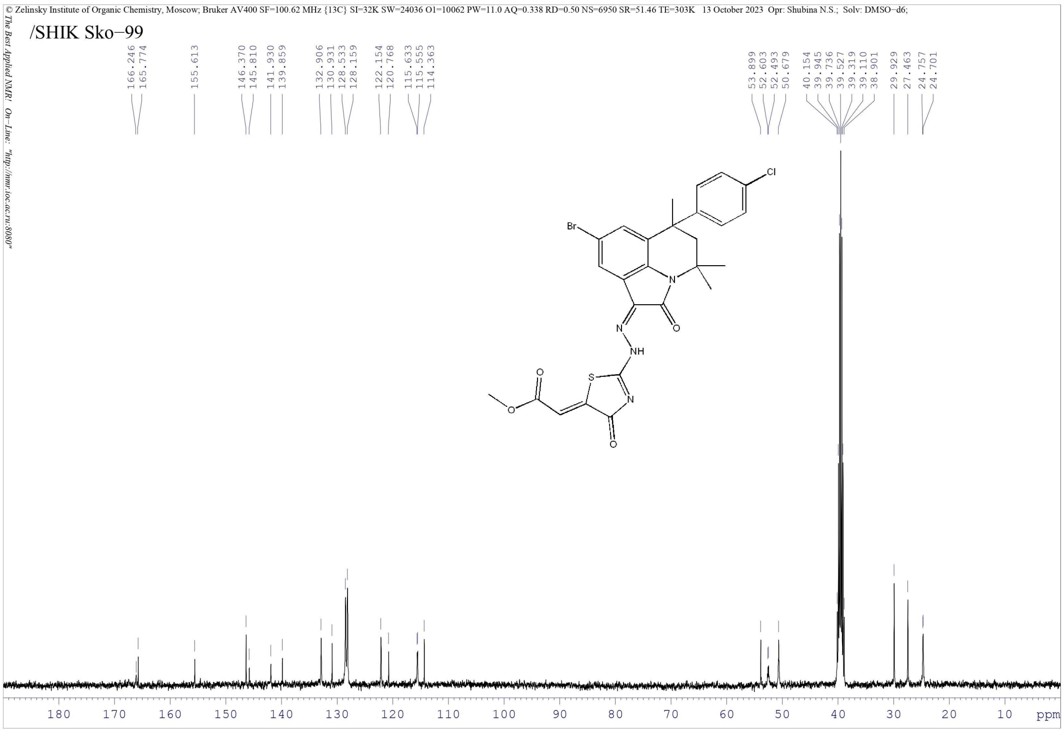

Figure S35. ^{13}C NMR spectrum of compound **3l**

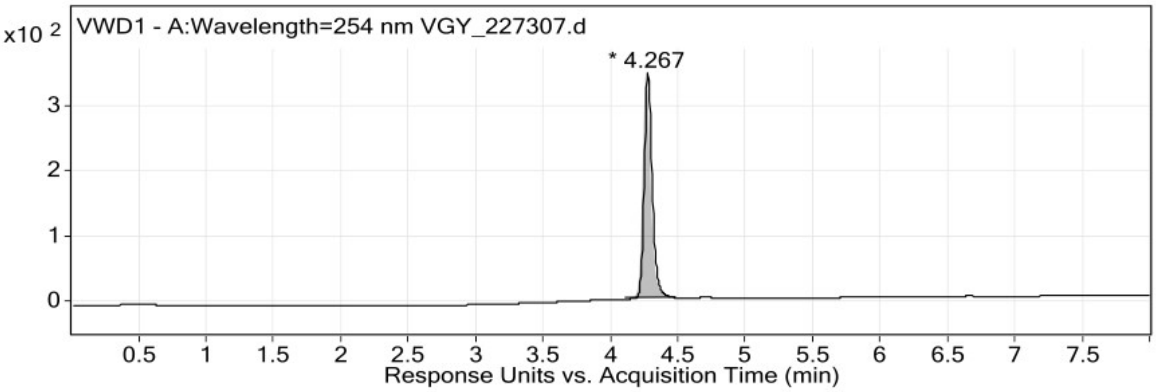

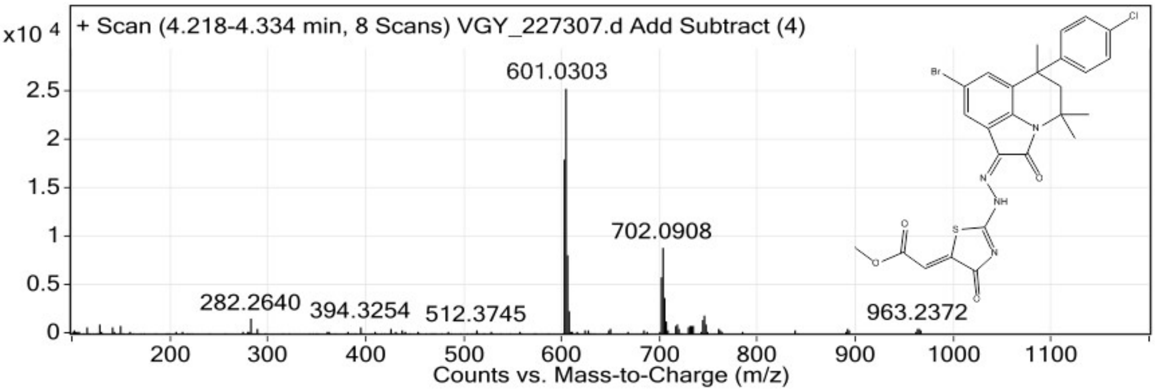

Figure S36. Data of HPLC-MS-ESI analysis of **3l**

^{1}H, ^{13}C NMR and data HPLC-HRMS-ESI spectra of methyl 2-(2-(2-(6-(4-chlorophenyl)-8-fluoro-4,4,6-trimethyl-2-oxo-5,6-dihydro-4*H*-pyrrolo[3,2,1-*ij*]quinolin-1(2*H*)-ylidene)hydrazineyl)-4-oxothiazol-5(4*H*)-ylidene)acetate 3m

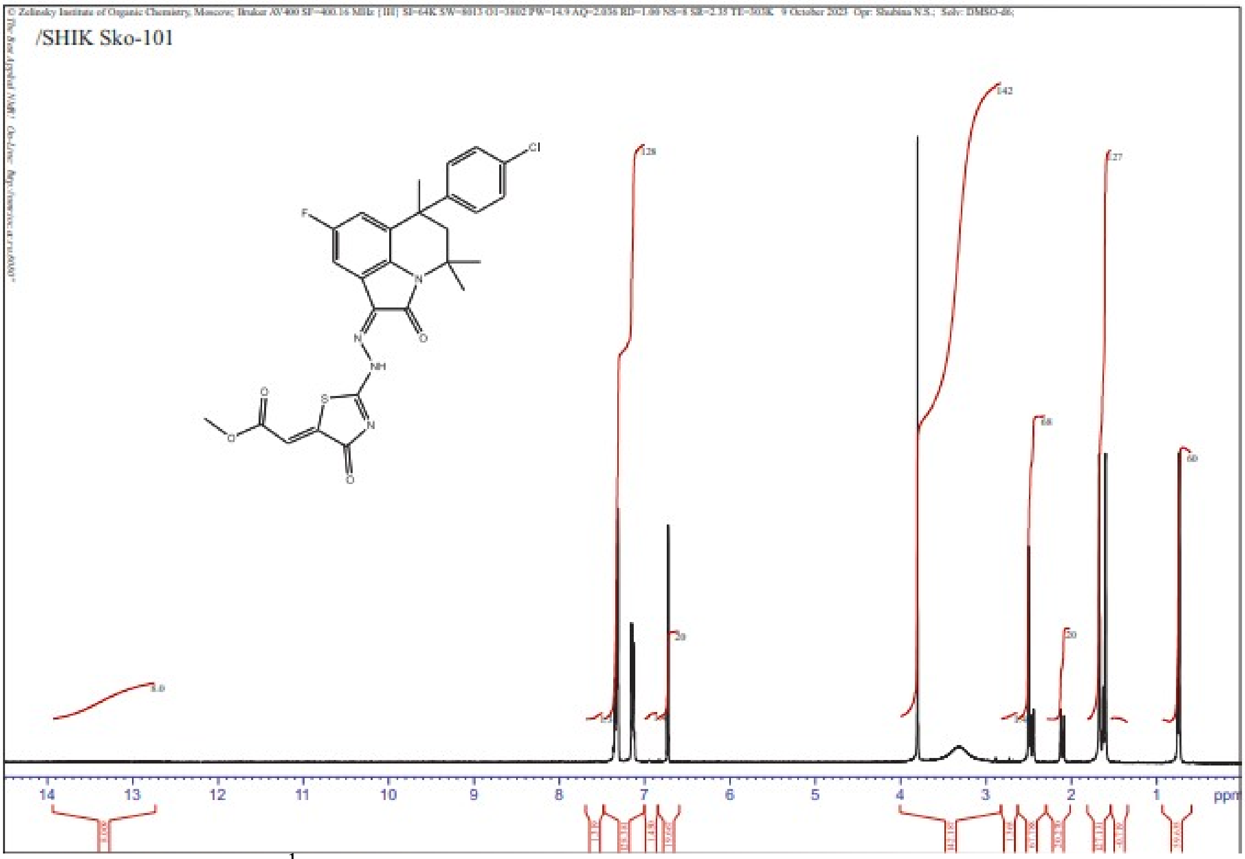

Figure S37. ^{1}H NMR spectrum of compound **3m**

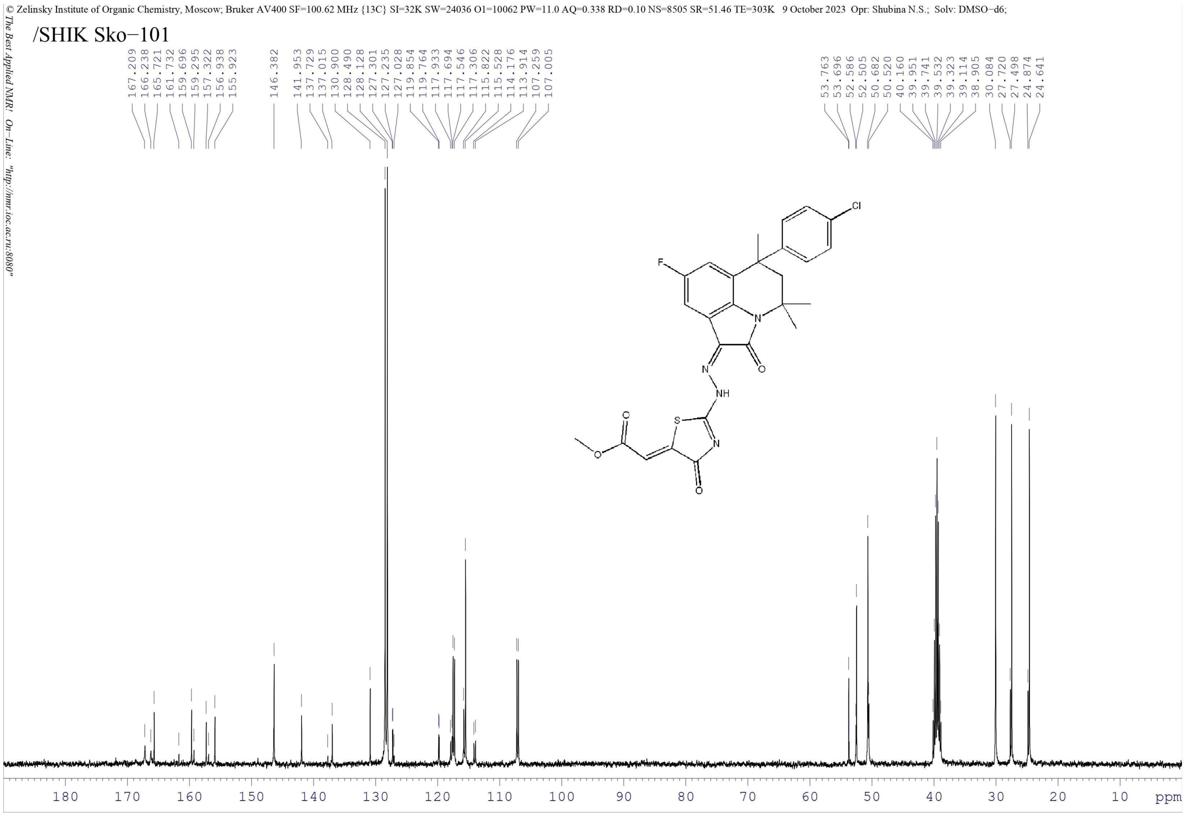

Figure S38. ^{13}C NMR spectrum of compound **3m**

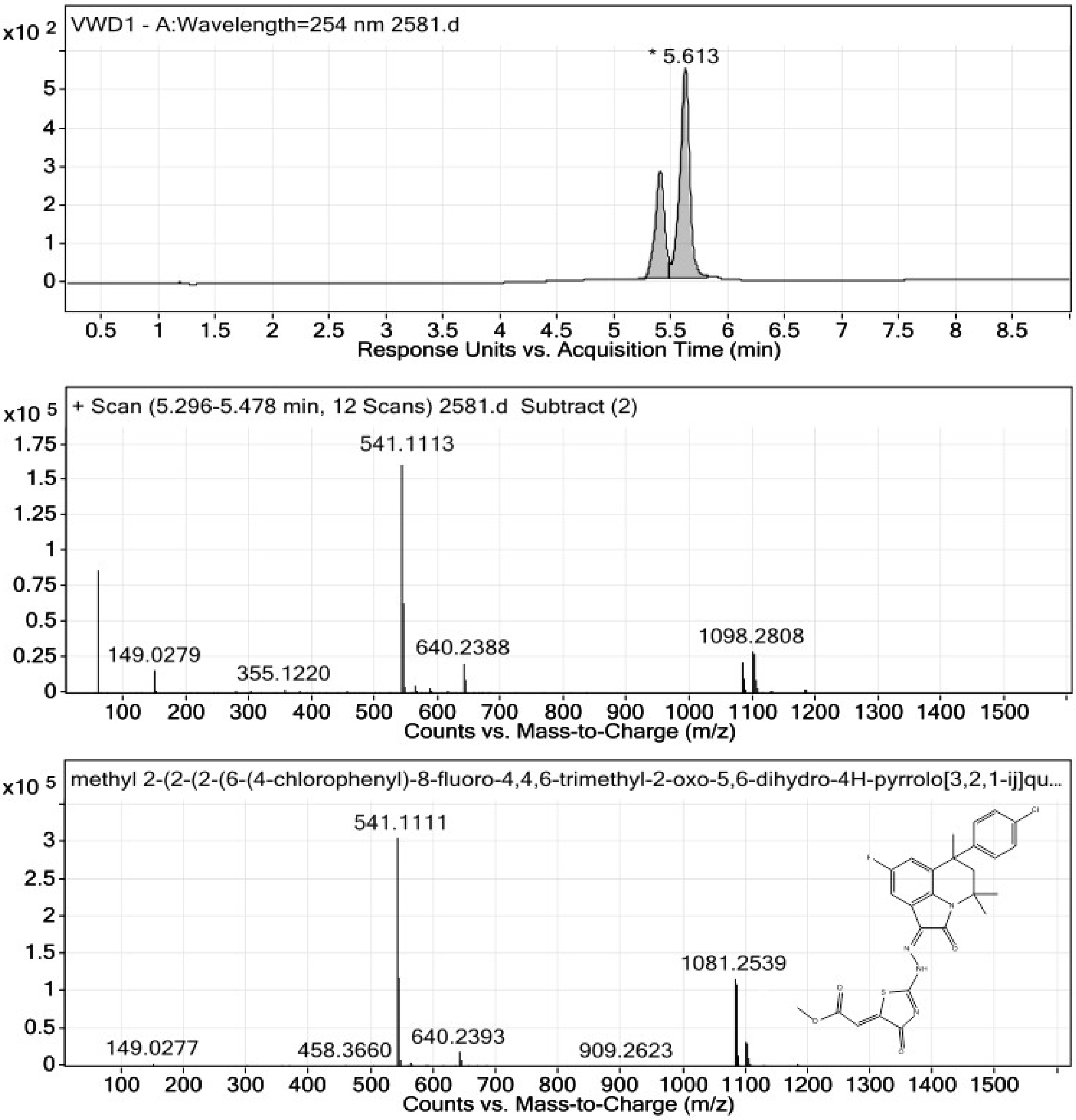

Figure S39. Data of HPLC-MS-ESI analysis of **3m**

^{1}H, ^{13}C NMR and data HPLC-HRMS-ESI spectra of methyl (Z)-2-(4-oxo-2-(2-((Z)-4,4,6-trimethyl-2-oxo-6-phenyl-5,6-dihydro-4*H*-pyrrolo[3,2,1-*ij*]quinolin-1(2*H*)-ylidene)hydrazineyl)thiazol-5(4*H*)-ylidene)acetate 3n

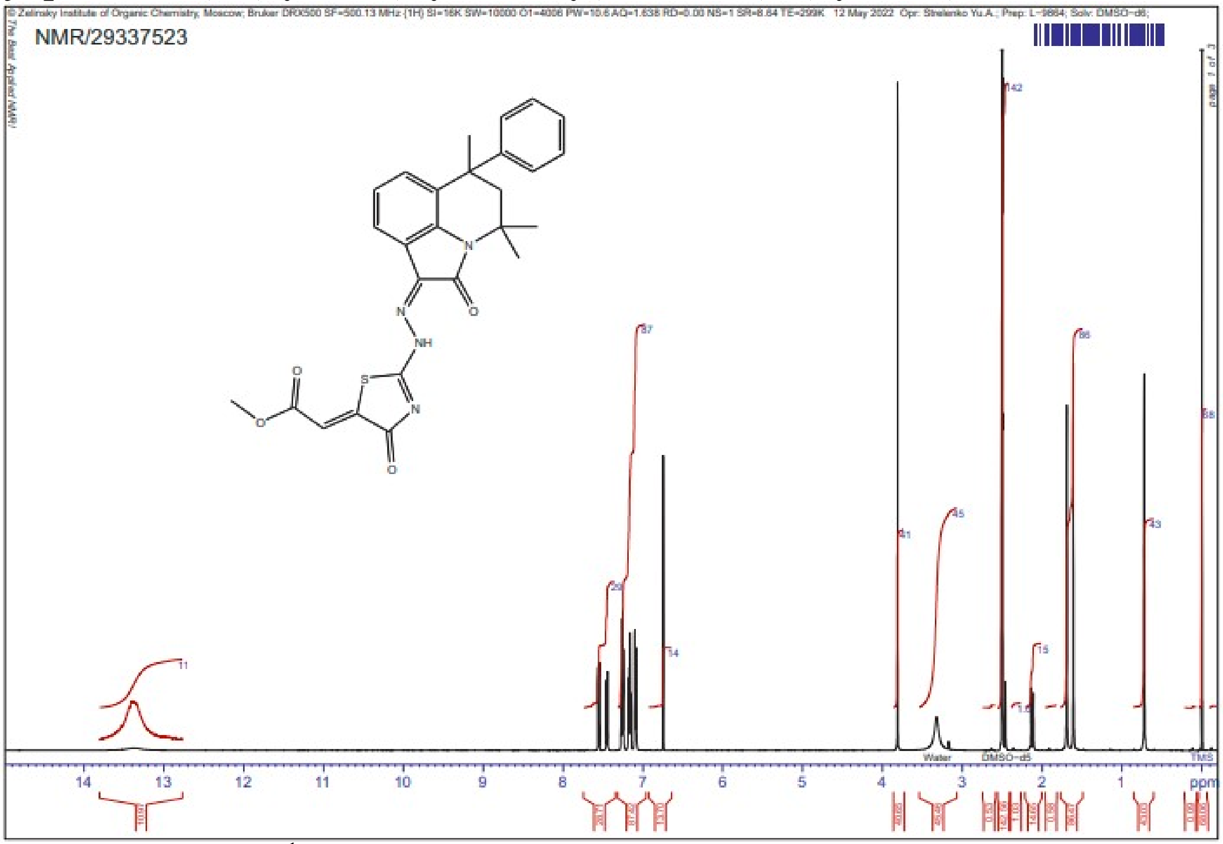

Figure S40. ^{1}H NMR spectrum of compound **3n**

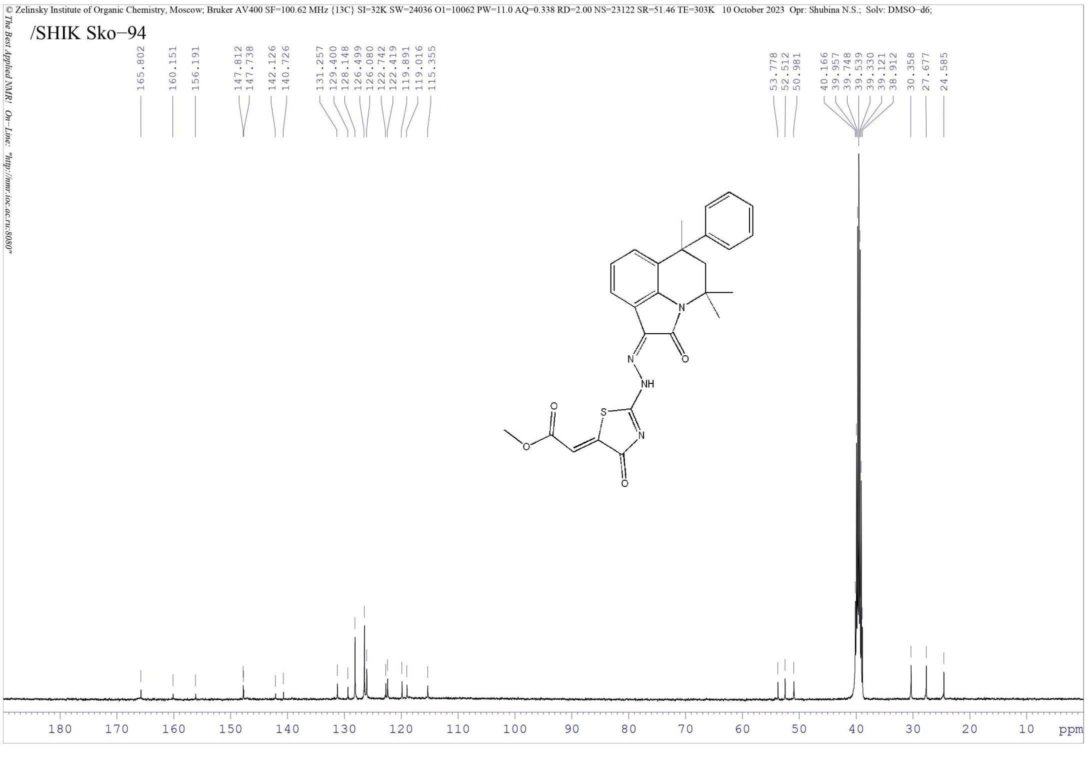

Figure S41. ^{13}C NMR spectrum of compound **3n**

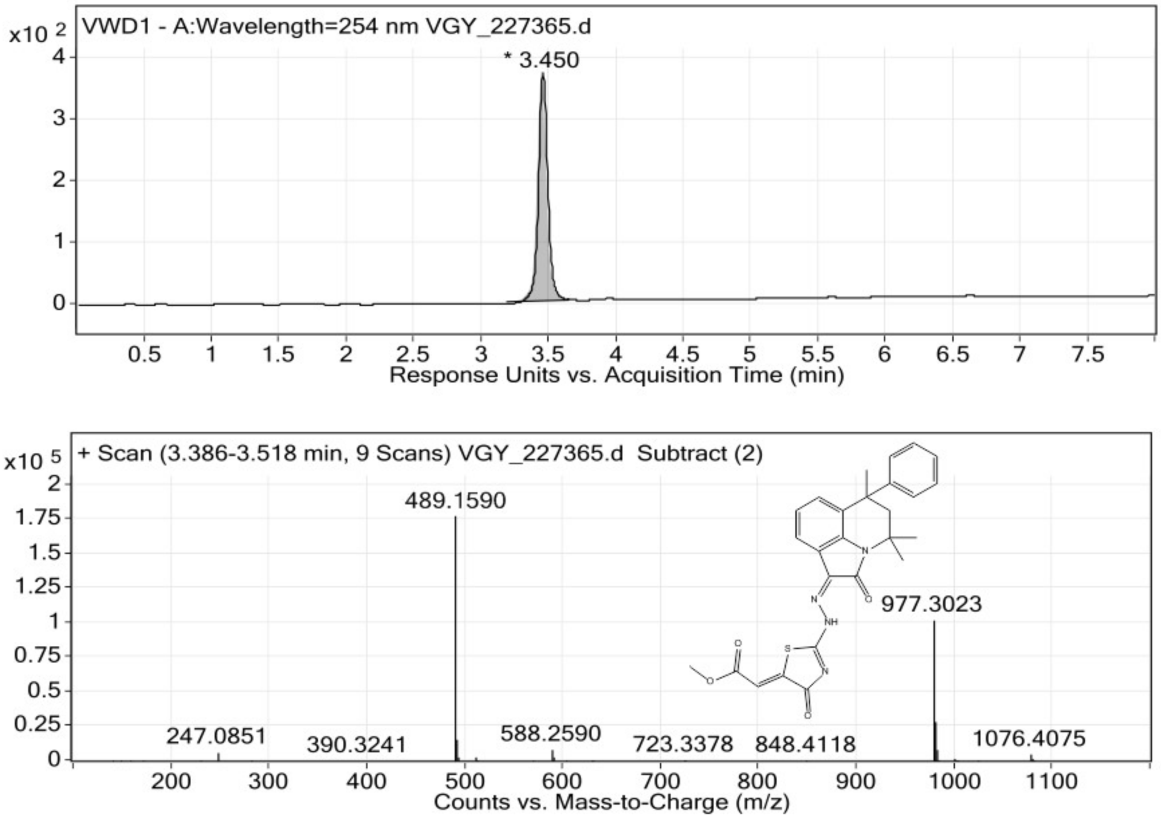

Figure S42. Data of HPLC-MS-ESI analysis of **3n**

^{1}H, ^{13}C NMR and data HPLC-HRMS-ESI spectra of methyl (Z)-2-(2-(2-((Z)-8-chloro-4,4,6-trimethyl-2-oxo-6-phenyl-5,6-dihydro-4*H*-pyrrolo[3,2,1-*ij*]quinolin-1(2*H*)-ylidene)hydrazineyl)-4-oxothiazol-5(4*H*)-ylidene)acetate 3o

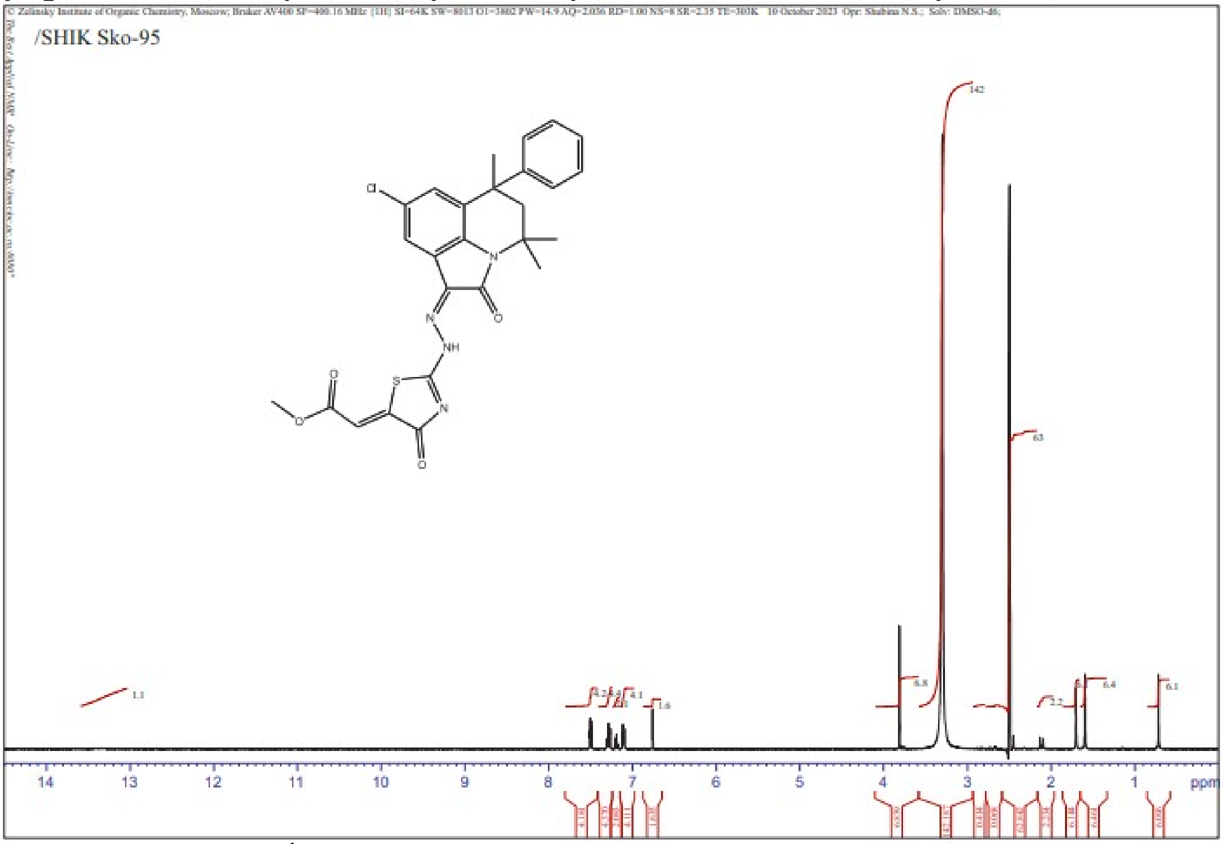

Figure S43. ^{1}H NMR spectrum of compound **3o**

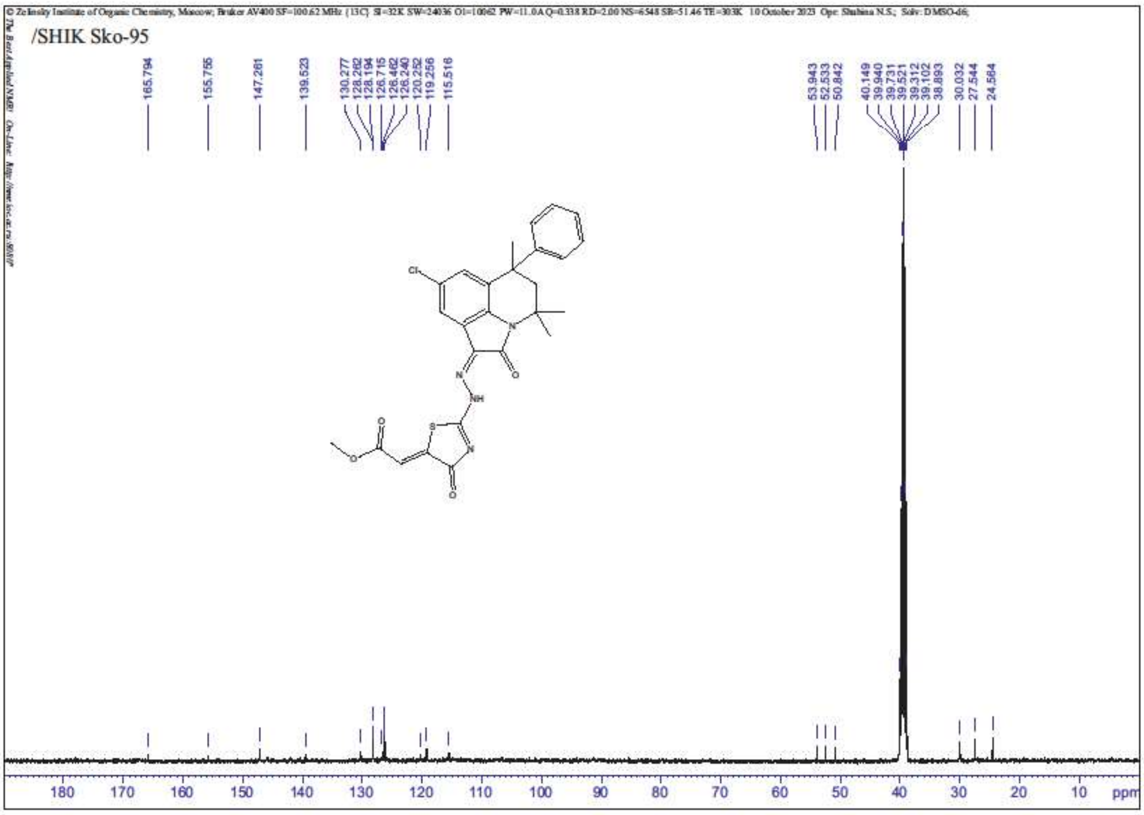

Figure S44. ^{13}C NMR spectrum of compound **3o**

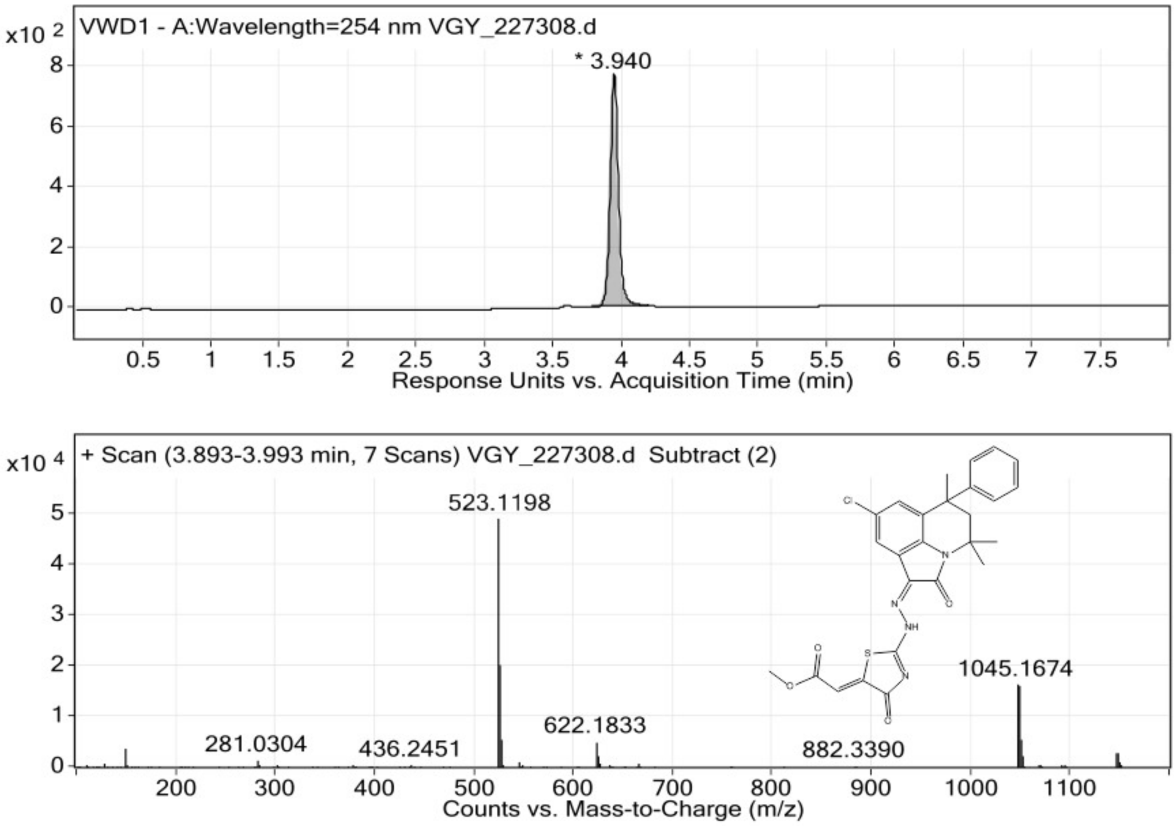

Figure S45. Data of HPLC-MS-ESI analysis of **3o**

^{1}H, ^{13}C NMR and data HPLC-HRMS-ESI spectra of methyl (Z)-2-(2-(2-((Z)-8-bromo-4,4,6-trimethyl-2-oxo-6-phenyl-5,6-dihydro-4*H*-pyrrolo[3,2,1-*ij*]quinolin-1(2*H*)-ylidene)hydrazineyl)-4-oxothiazol-5(4*H*)-ylidene)acetate 3p

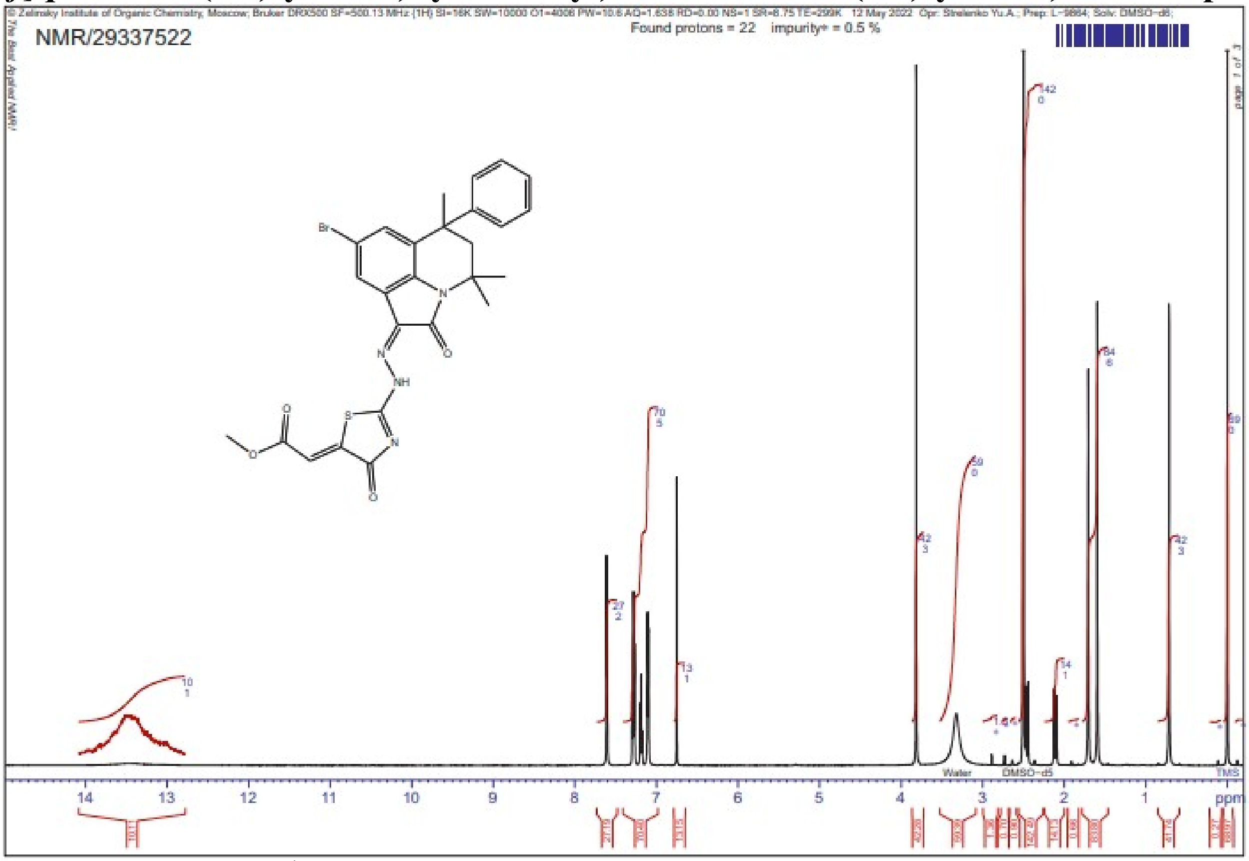

Figure S46. ^{1}H NMR spectrum of compound **3p**

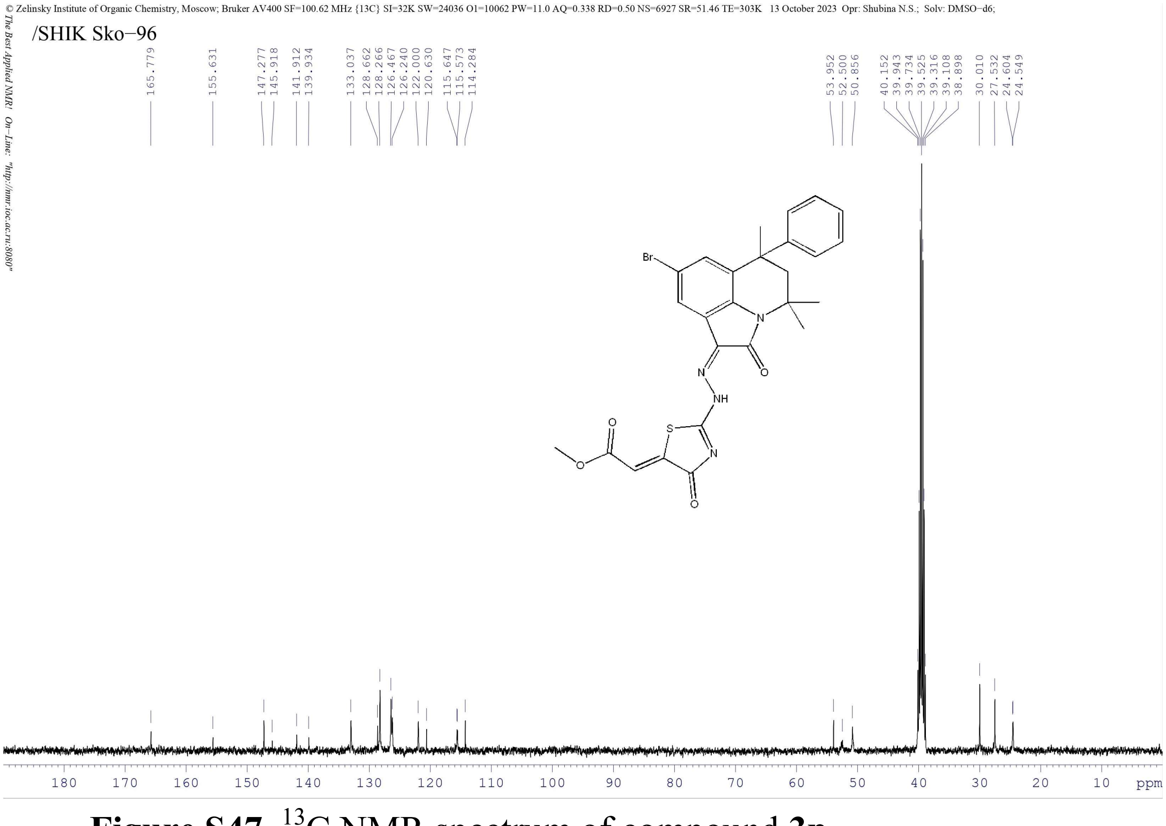

Figure S47. ^{13}C NMR spectrum of compound **3p**

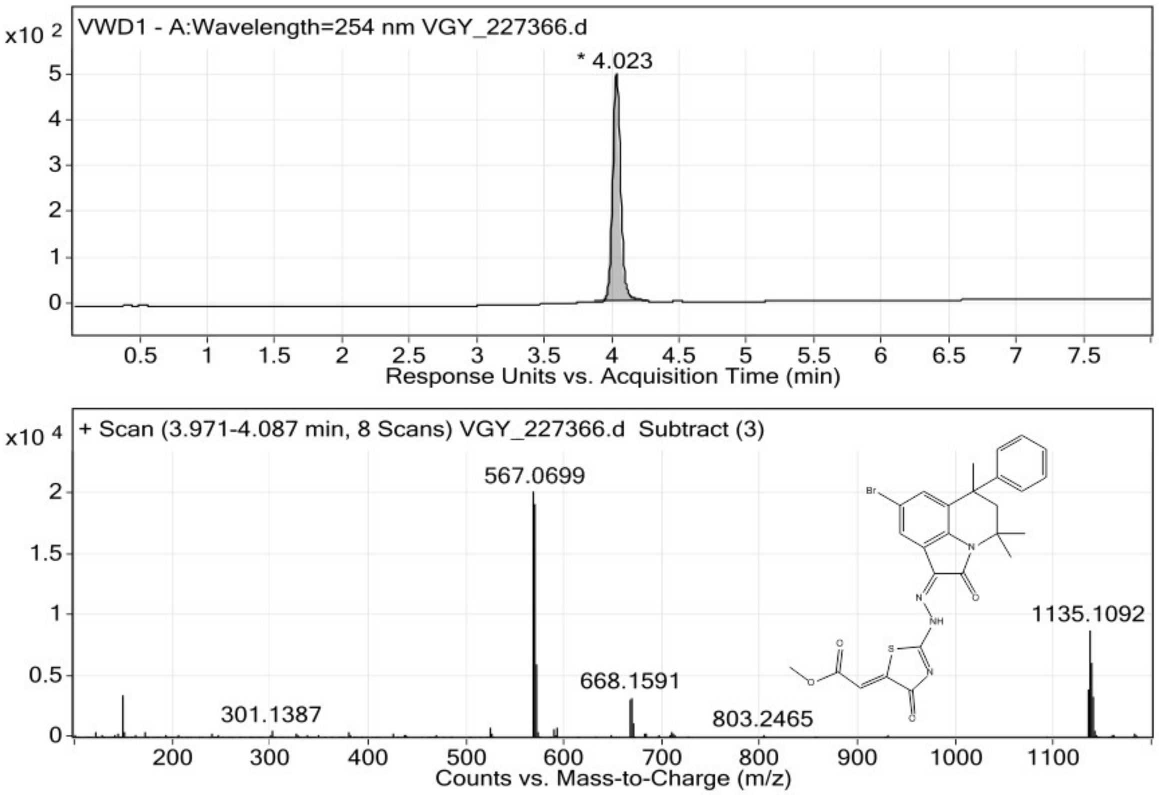

Figure S48. Data of HPLC-MS-ESI analysis of **3p**

^{1}H, ^{13}C NMR and data HPLC-HRMS-ESI spectra of methyl (Z)-2-(2-(2-((Z)-8-fluoro-4,4,6-trimethyl-2-oxo-6-phenyl-5,6-dihydro-4*H*-pyrrolo[3,2,1-*ij*]quinolin-1(2*H*)-ylidene)hydrazineyl)-4-oxothiazol-5(4*H*)-ylidene)acetate 3q

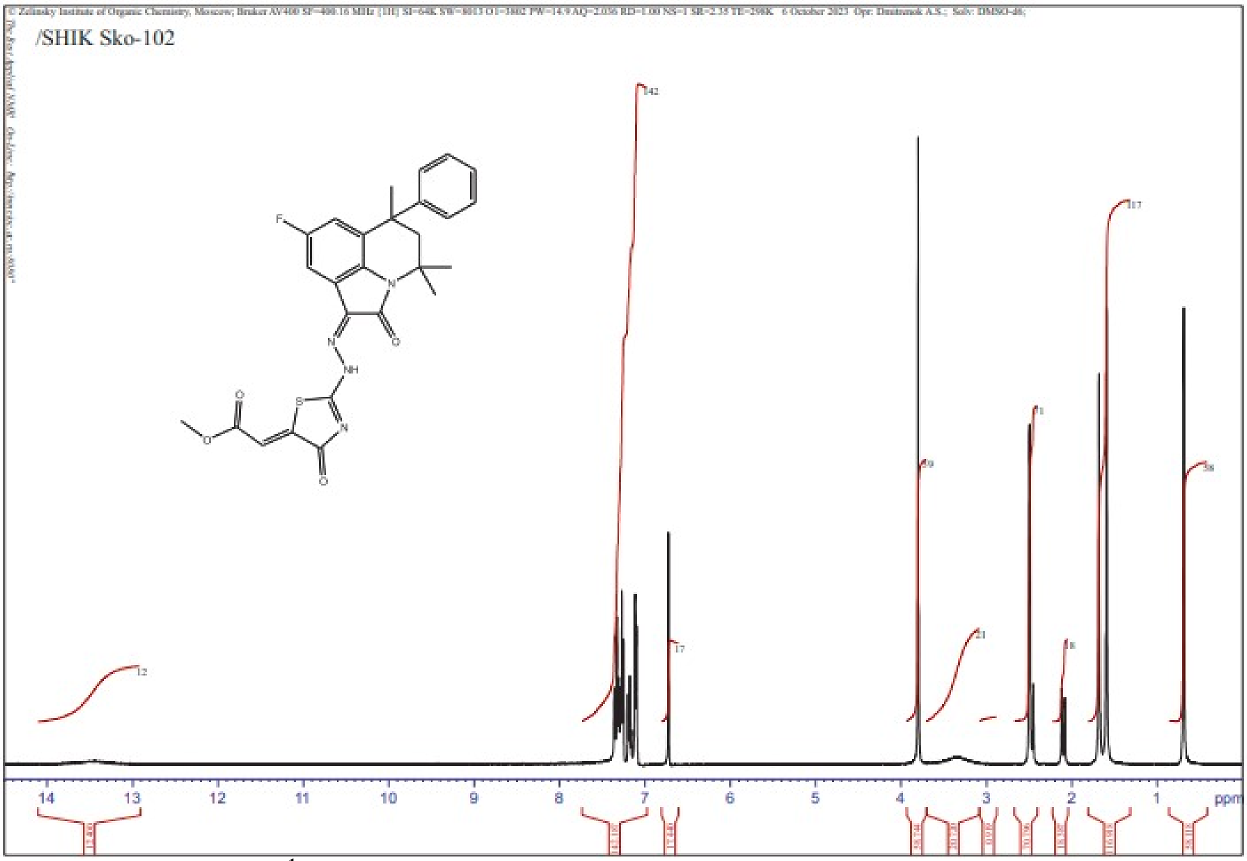

Figure S49. ^{1}H NMR spectrum of compound **3q**

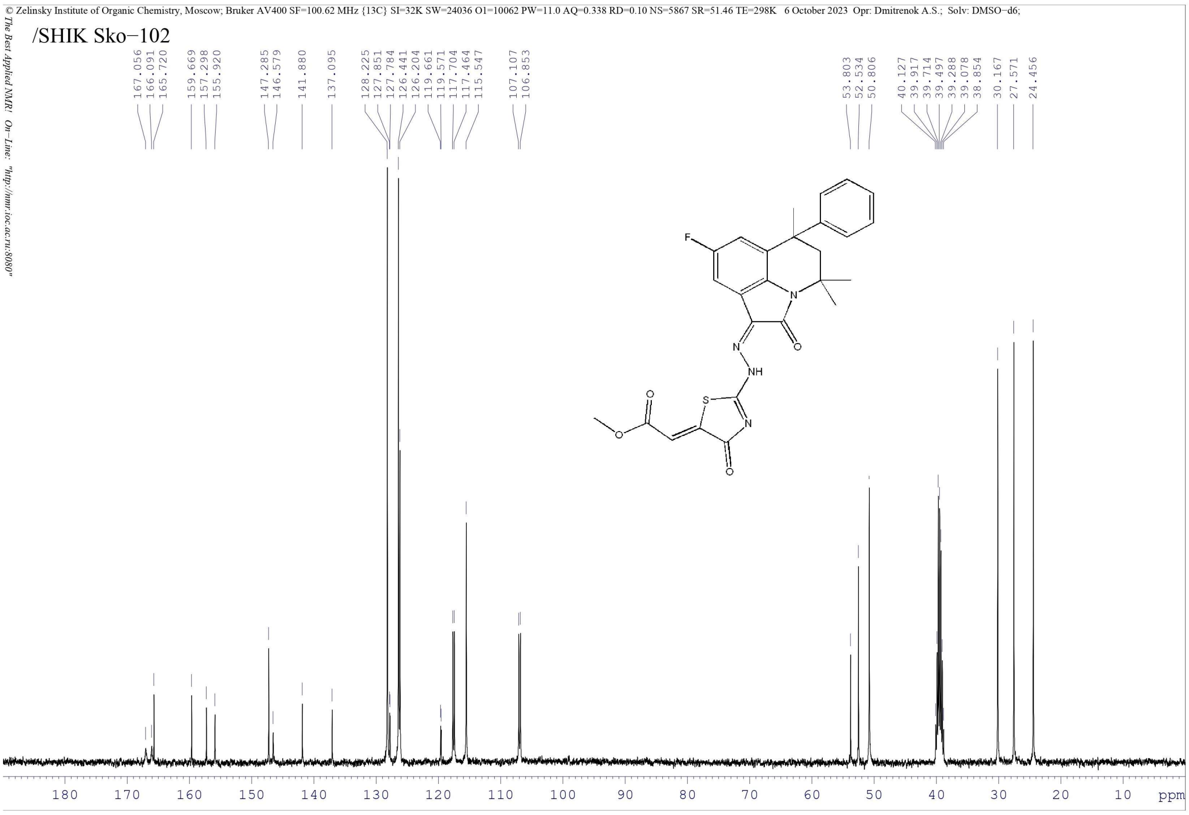

Figure S50. ^{13}C NMR spectrum of compound **3q**

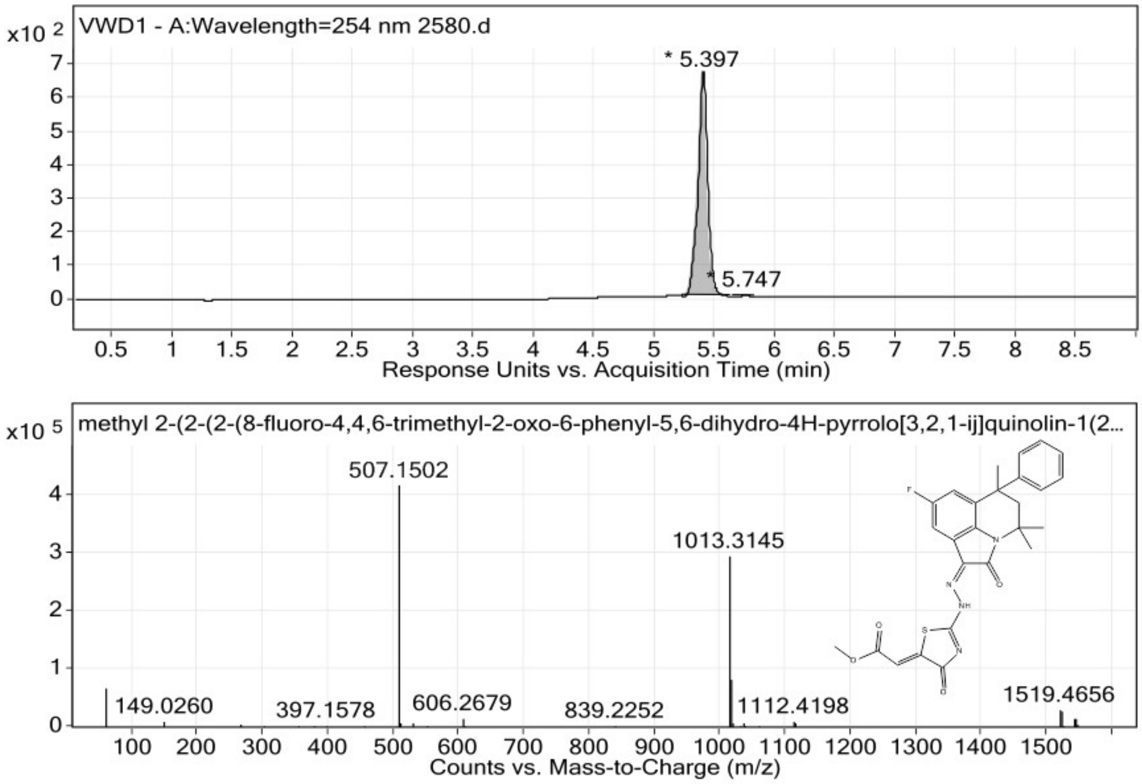

Figure S51. Data of HPLC-MS-ESI analysis of **3q**

The dependence of inhibition of factor Xa- and XIa-induced chromogenic substrate hydrolysis on the compound concentration.

The dependence of inhibition of factor Xa- and XIa-induced chromogenic substrate hydrolysis on the concentration of 3b

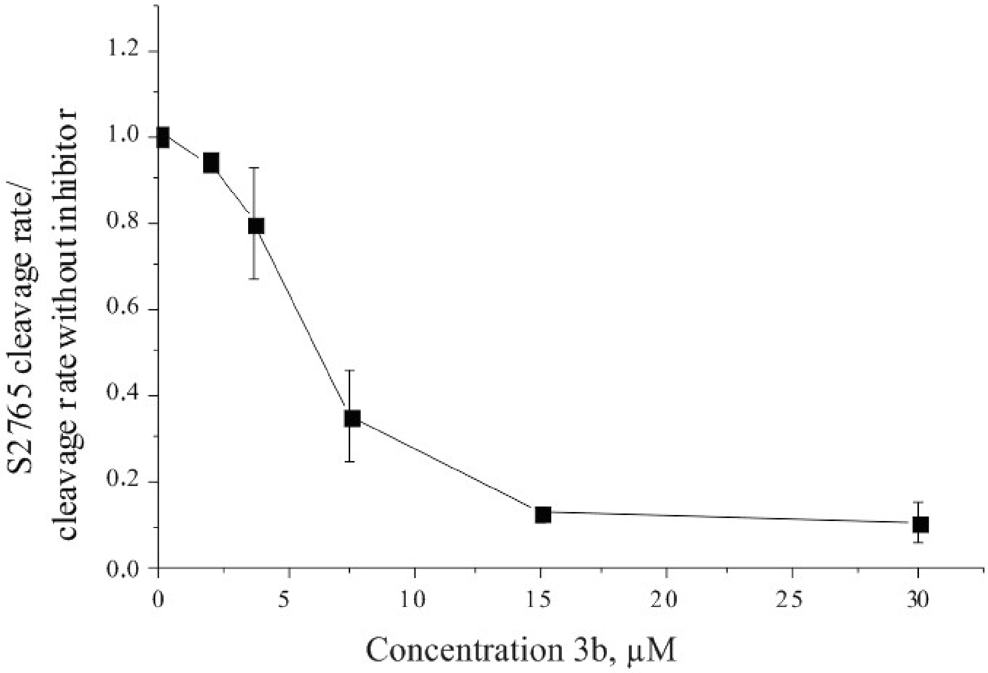

Figure S52. The dependence of inhibition of factor Xa-induced chromogenic substrate hydrolysis on the concentration of **3b**

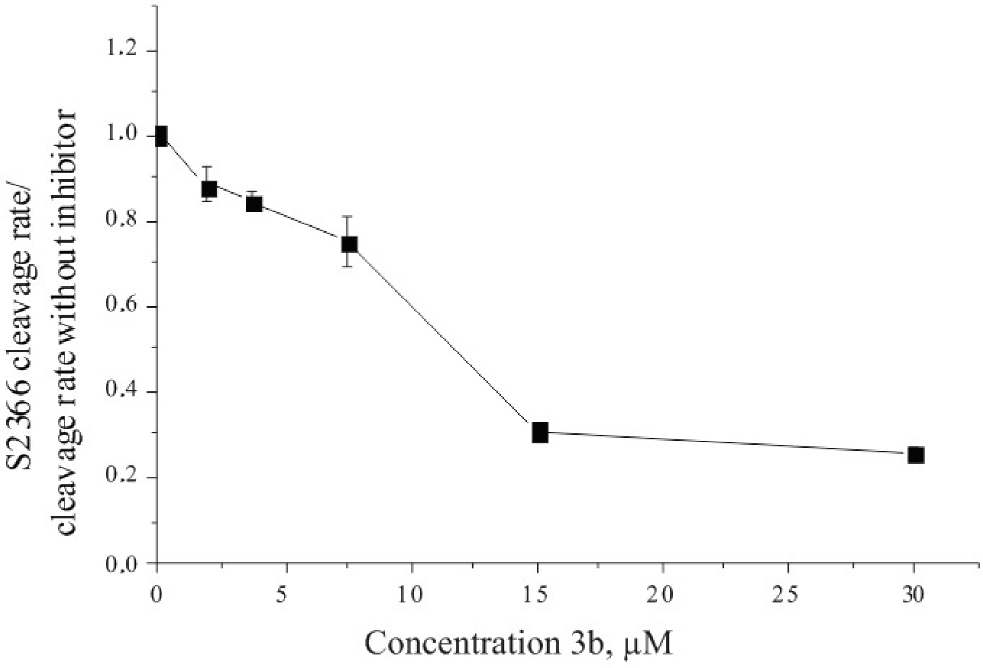

Figure S53. The dependence of inhibition of factor XIa-induced chromogenic substrate hydrolysis on the concentration of **3b**

The dependence of inhibition of factor Xa- and XIa-induced chromogenic substrate hydrolysis on the concentration of 3c

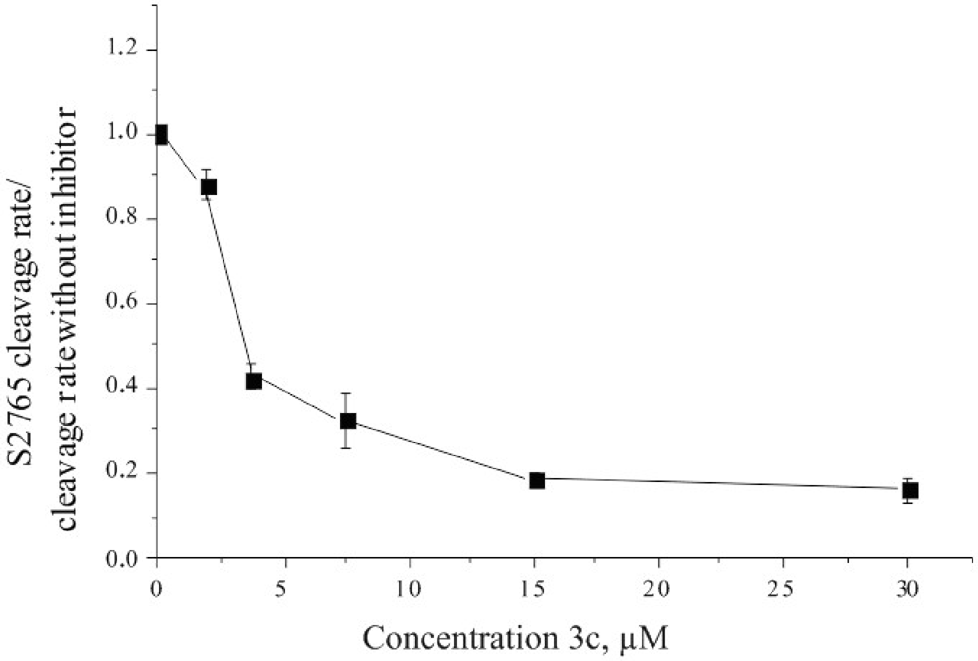

Figure S54. The dependence of inhibition of factor Xa-induced chromogenic substrate hydrolysis on the concentration of **3c**

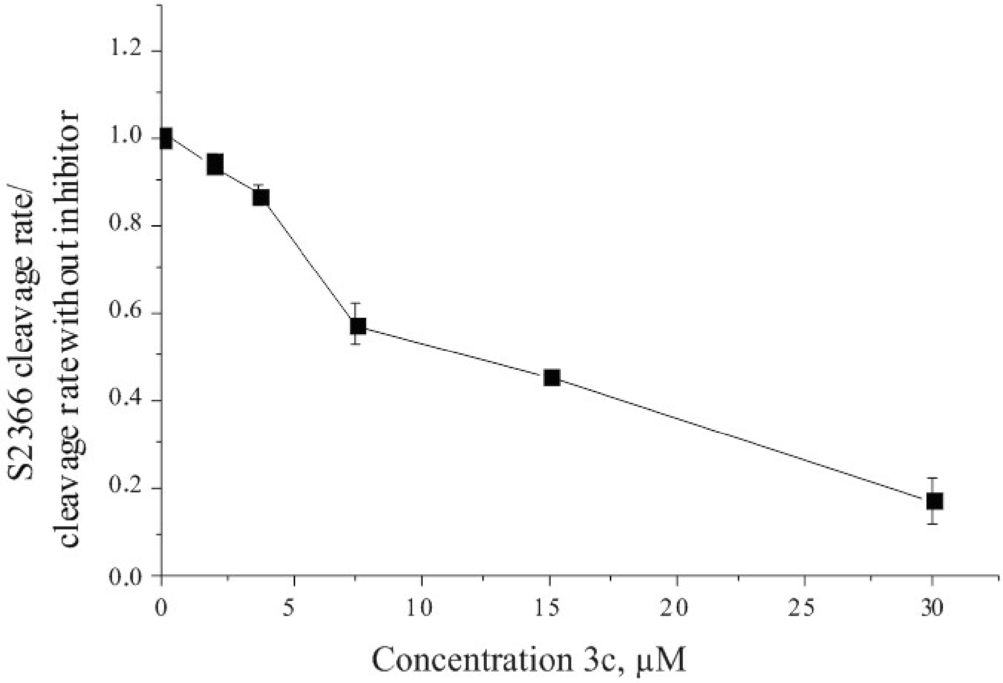

Figure S55. The dependence of inhibition of factor XIa-induced chromogenic substrate hydrolysis on the concentration of **3c**

The dependence of inhibition of factor Xa- and XIa-induced chromogenic substrate hydrolysis on the concentration of 3e

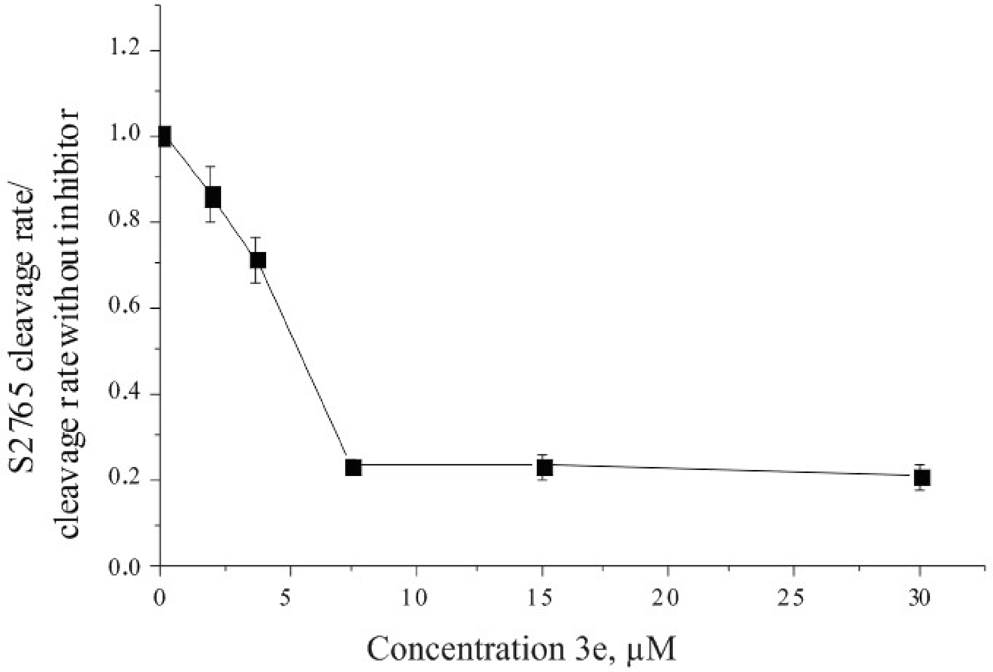

Figure S56. The dependence of inhibition of factor Xa-induced chromogenic substrate hydrolysis on the concentration of **3e**

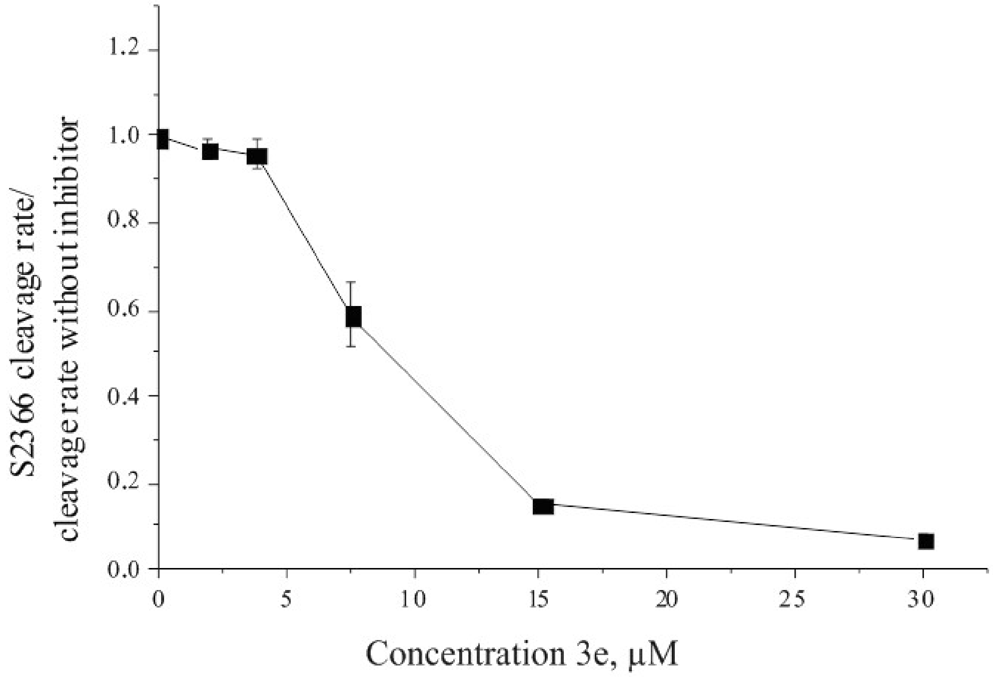

Figure S57. The dependence of inhibition of factor XIa-induced chromogenic substrate hydrolysis on the concentration of **3e**

The dependence of inhibition of factor Xa- and XIa-induced chromogenic substrate hydrolysis on the concentration of 3f

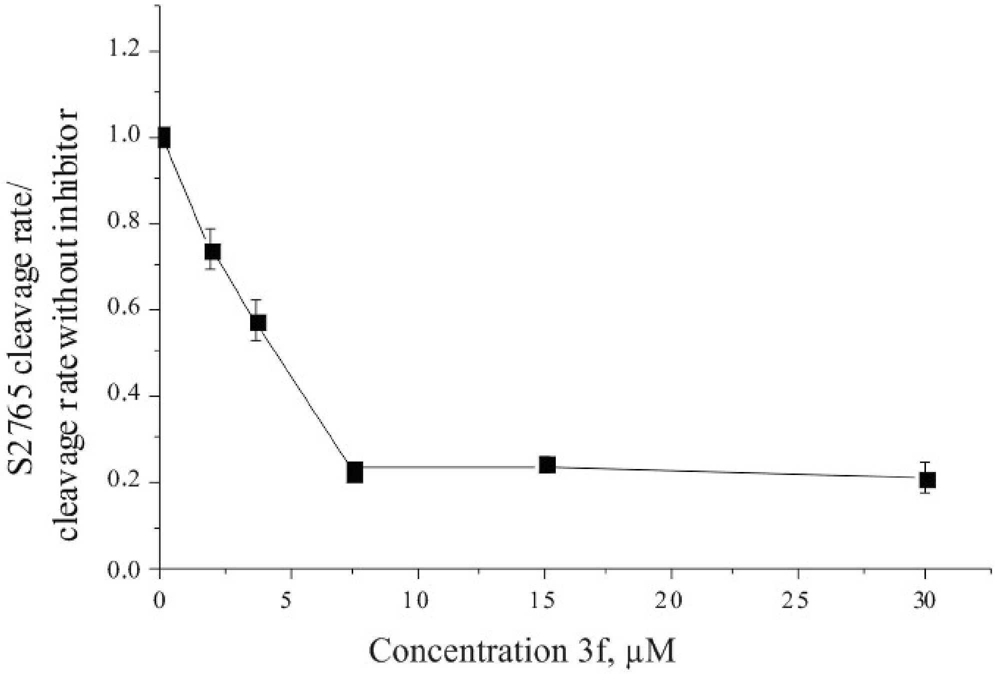

Figure S58. The dependence of inhibition of factor Xa-induced chromogenic substrate hydrolysis on the concentration of **3f**

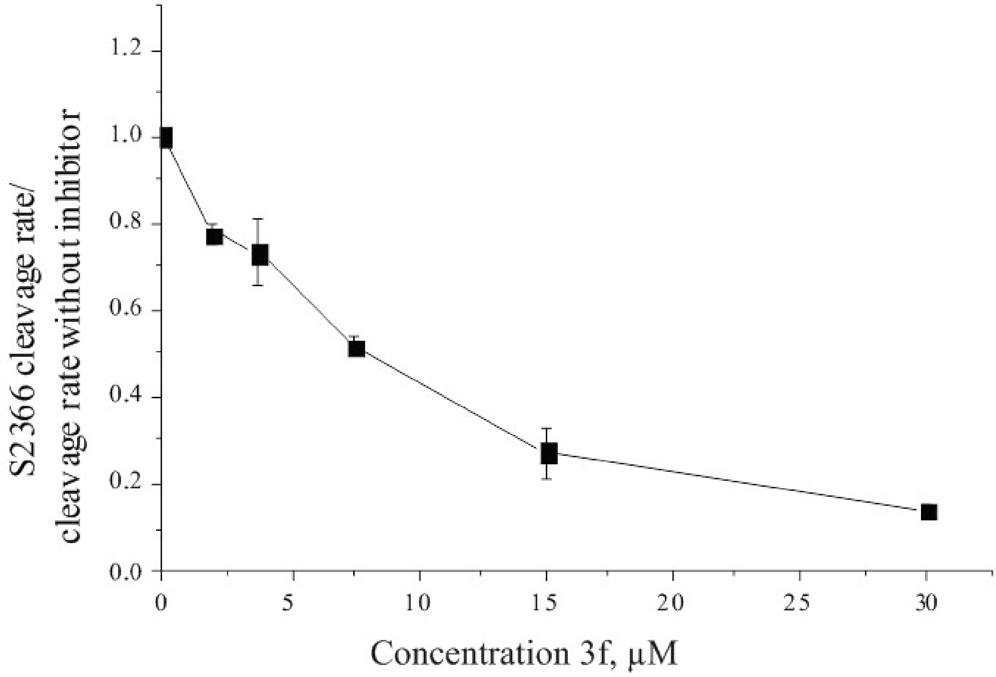

Figure S59. The dependence of inhibition of factor XIa-induced chromogenic substrate hydrolysis on the concentration of **3f**

The dependence of inhibition of factor Xa- and XIa-induced chromogenic substrate hydrolysis on the concentration of 3k

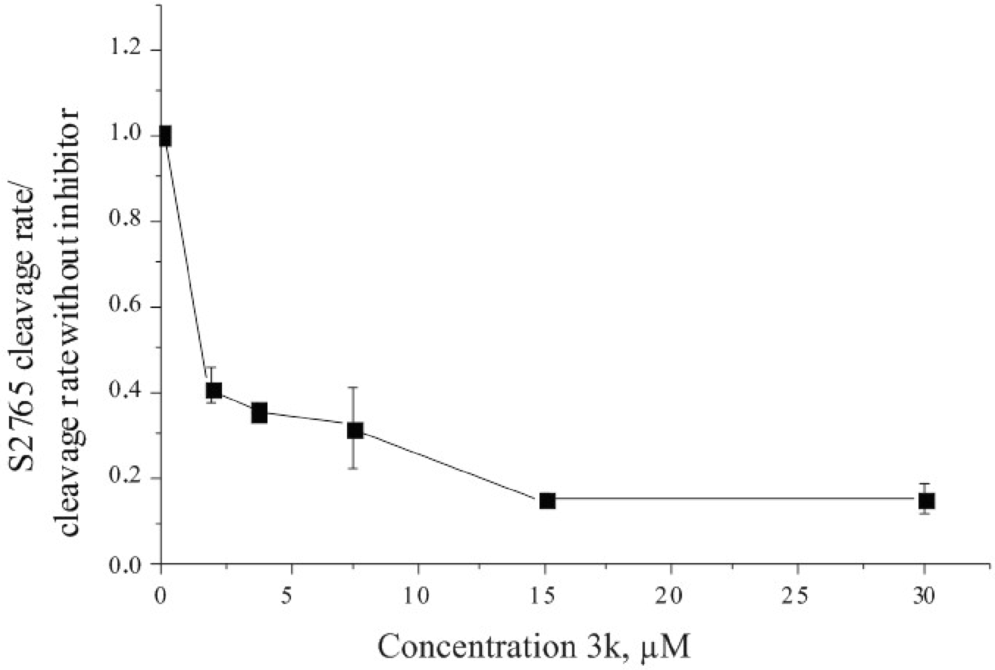

Figure S60. The dependence of inhibition of factor Xa-induced chromogenic substrate hydrolysis on the concentration of **3k**

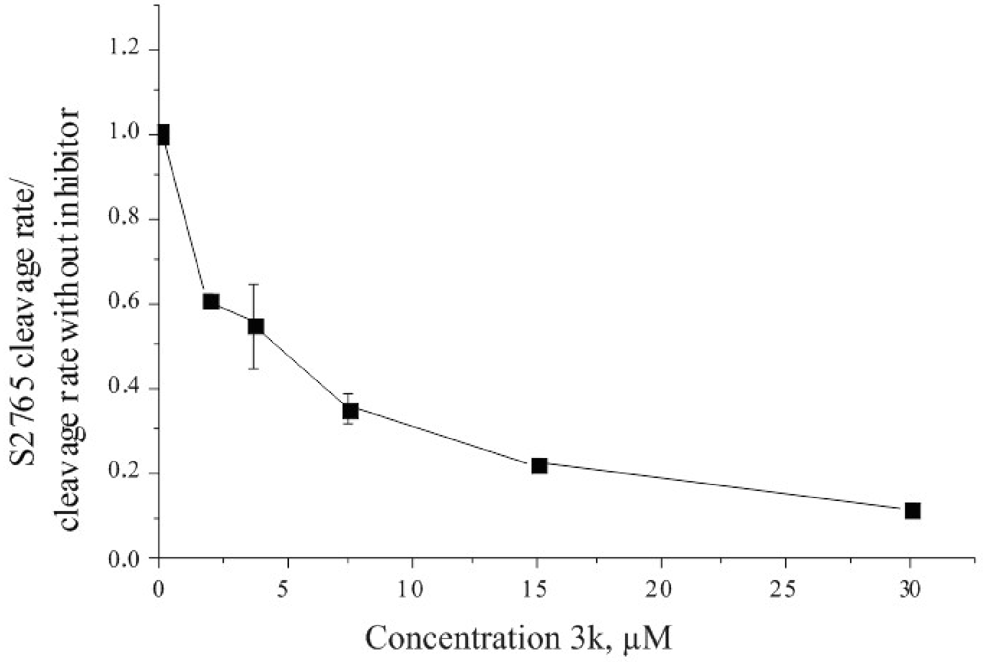

Figure S61. The dependence of inhibition of factor XIa-induced chromogenic substrate hydrolysis on the concentration of **3k**

The dependence of inhibition of factor Xa- and XIa-induced chromogenic substrate hydrolysis on the concentration of 3l

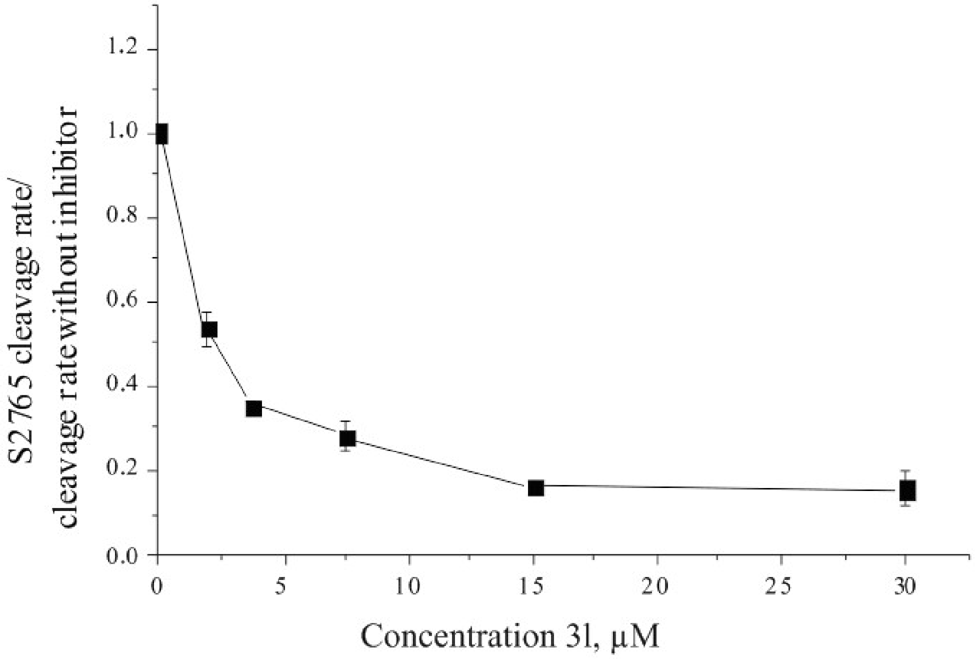

Figure S62. The dependence of inhibition of factor Xa-induced chromogenic substrate hydrolysis on the concentration of **3l**

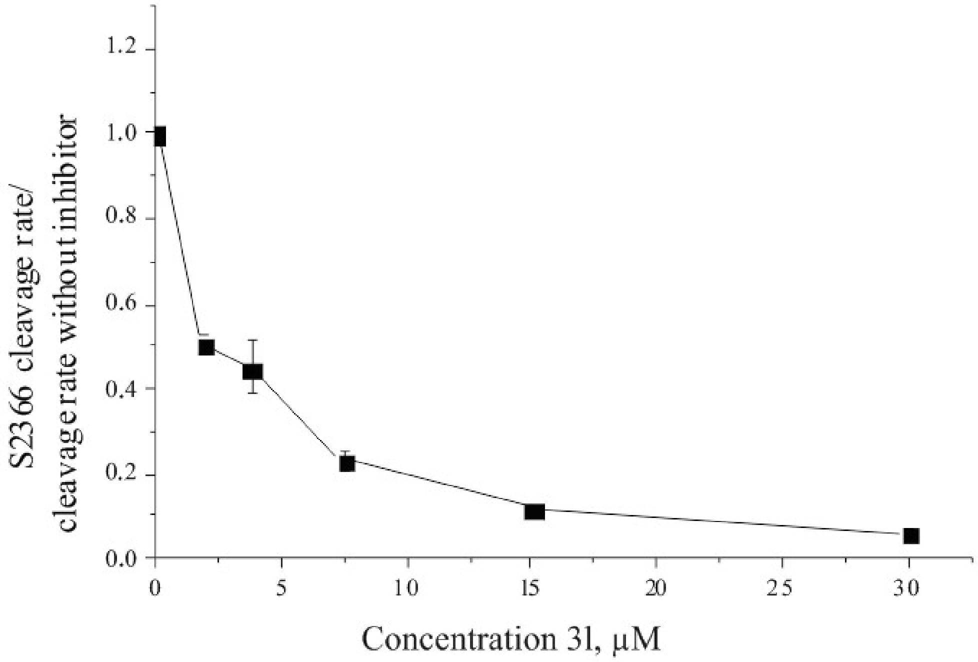

Figure S63. The dependence of inhibition of factor XIa-induced chromogenic substrate hydrolysis on the concentration of **3l**

The dependence of inhibition of factor Xa- and XIa-induced chromogenic substrate hydrolysis on the concentration of 3n

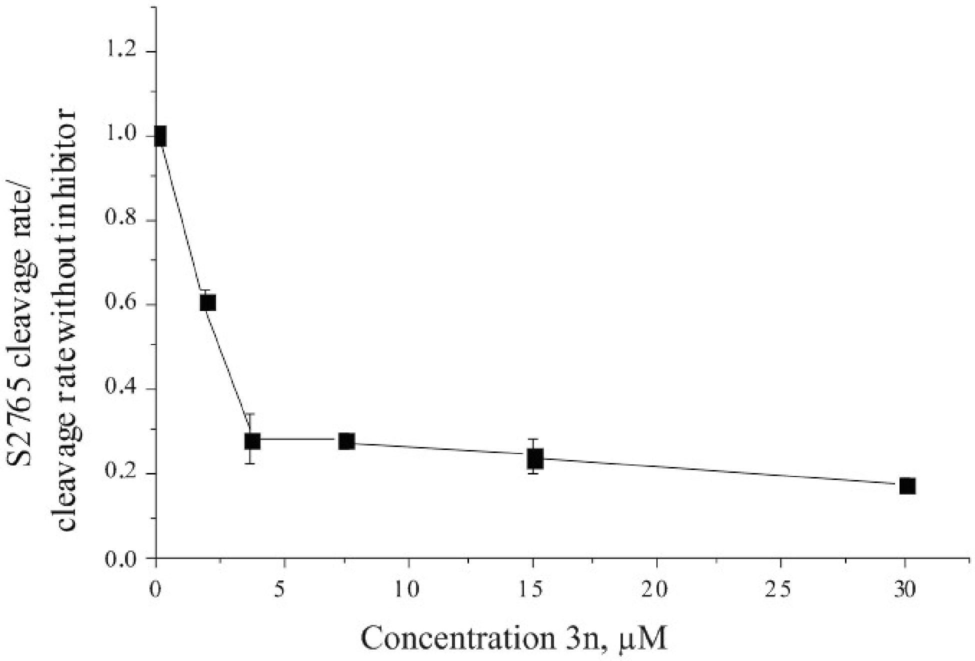

Figure S64. The dependence of inhibition of factor Xa-induced chromogenic substrate hydrolysis on the concentration of **3n**

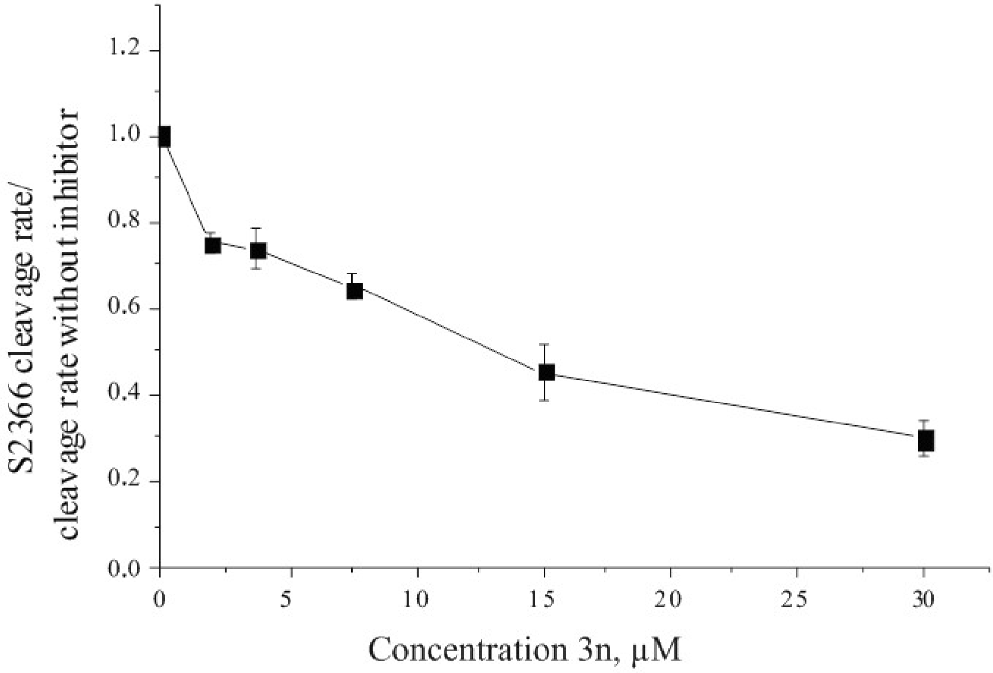

Figure S65. The dependence of inhibition of factor XIa-induced chromogenic substrate hydrolysis on the concentration of **3n**

The dependence of inhibition of factor Xa- and XIa-induced chromogenic substrate hydrolysis on the concentration of 3o

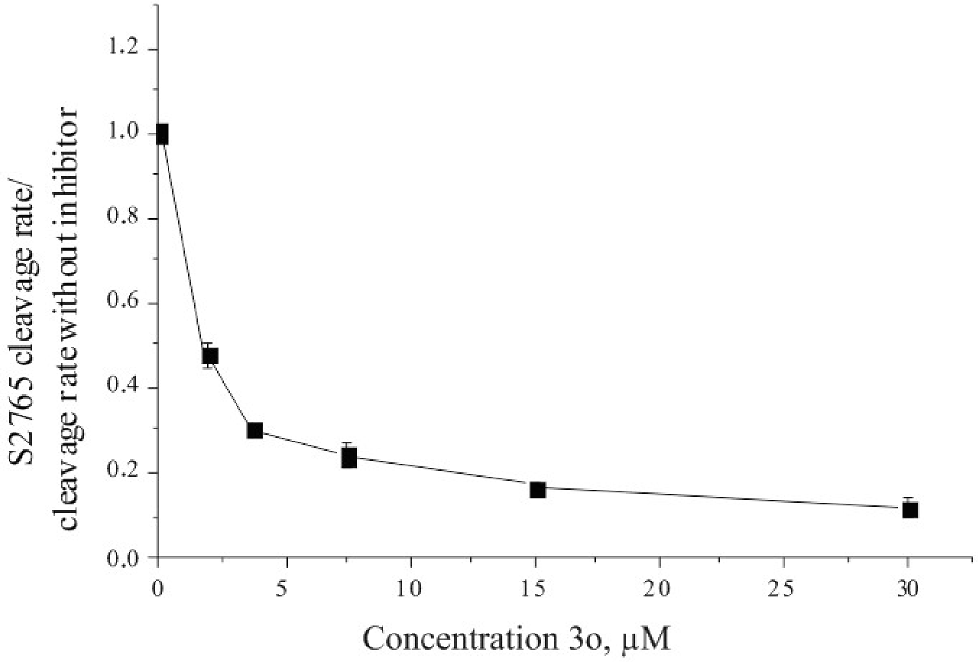

Figure S66. The dependence of inhibition of factor Xa-induced chromogenic substrate hydrolysis on the concentration of **3o**

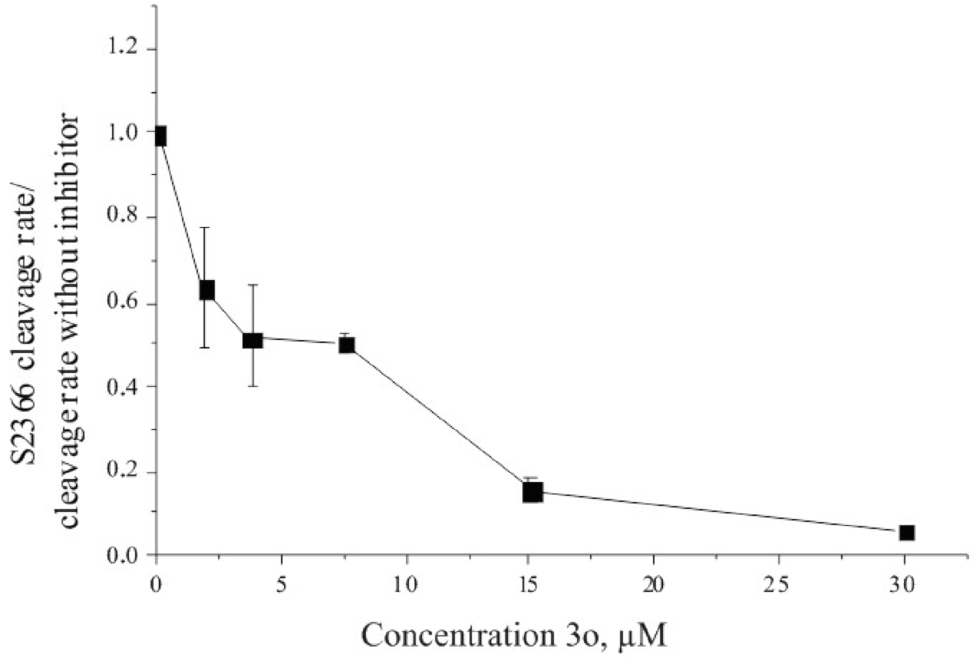

Figure S67. The dependence of inhibition of factor XIa-induced chromogenic substrate hydrolysis on the concentration of **3o**

The dependence of inhibition of factor Xa- and XIa-induced chromogenic substrate hydrolysis on the concentration of 3p

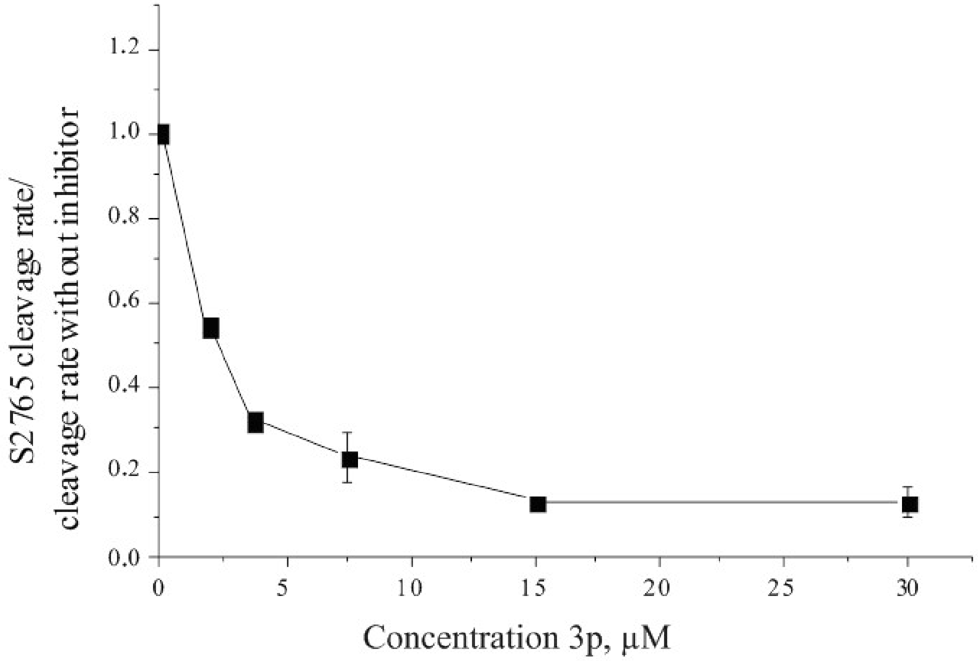

Figure S68. The dependence of inhibition of factor Xa-induced chromogenic substrate hydrolysis on the concentration of **3p**

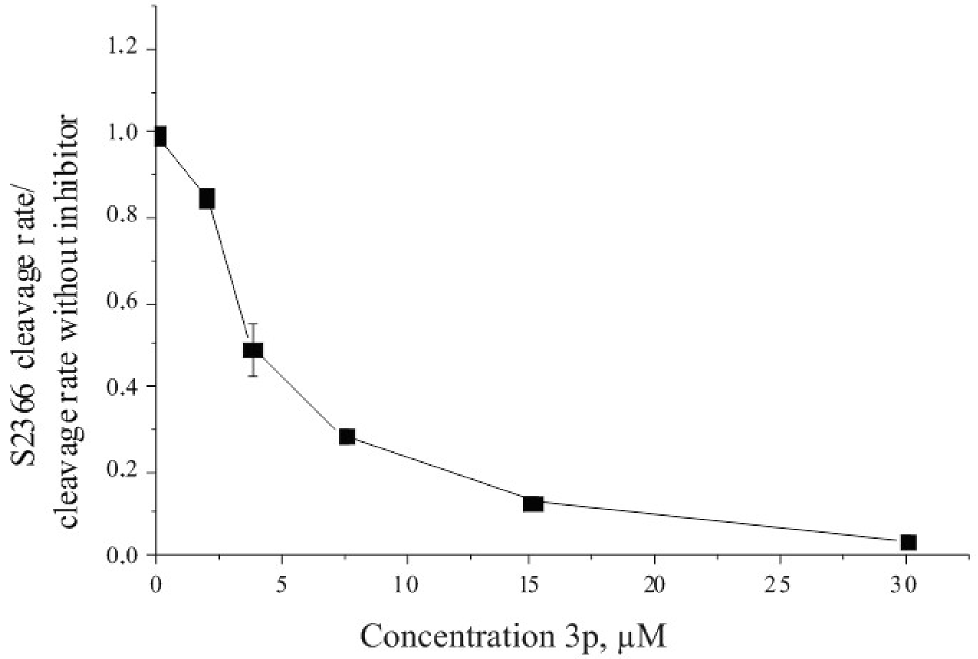

Figure S69. The dependence of inhibition of factor XIa-induced chromogenic substrate hydrolysis on the concentration of **3p**

LCMS analysis of the reaction mass after 1 h of interaction of compound 1b with thiosemicarbazide to obtain 2b

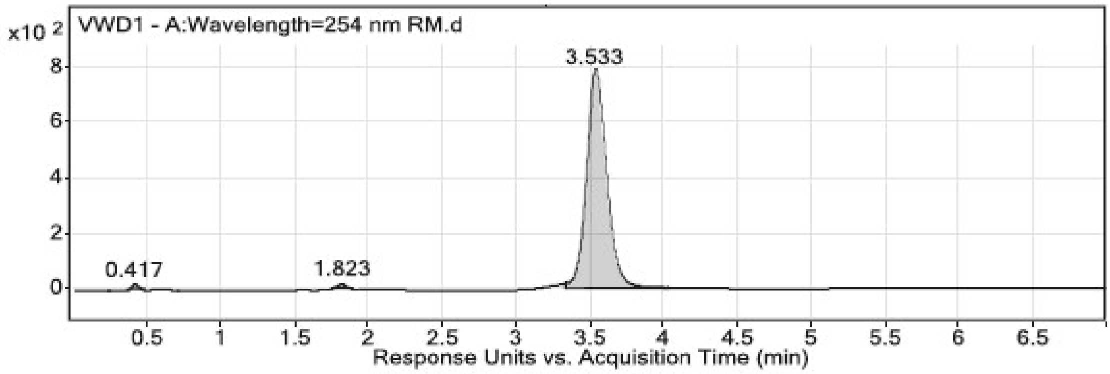

Integration Peak List

Peak	Start	RT	End	Area	Area %
1	0.359	0.417	0.489	137.86	1.78
2	1.748	1.823	1.912	120.82	1.56
3	3.34	3.533	4.037	7744.89	100

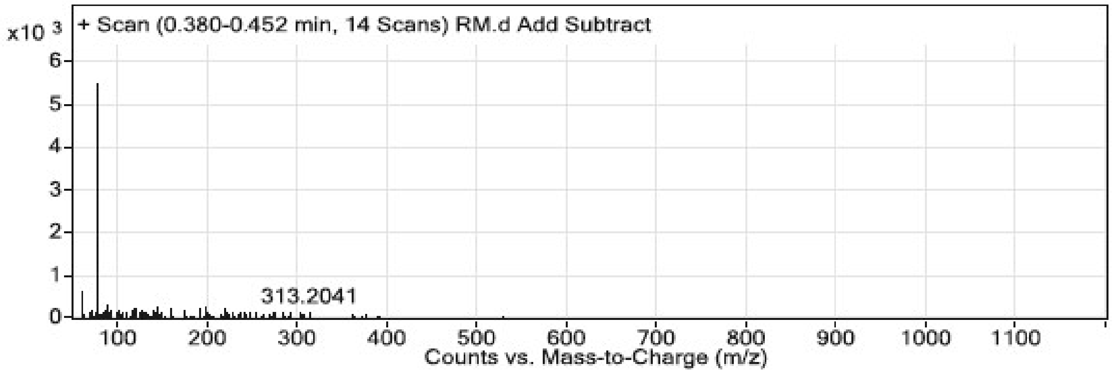

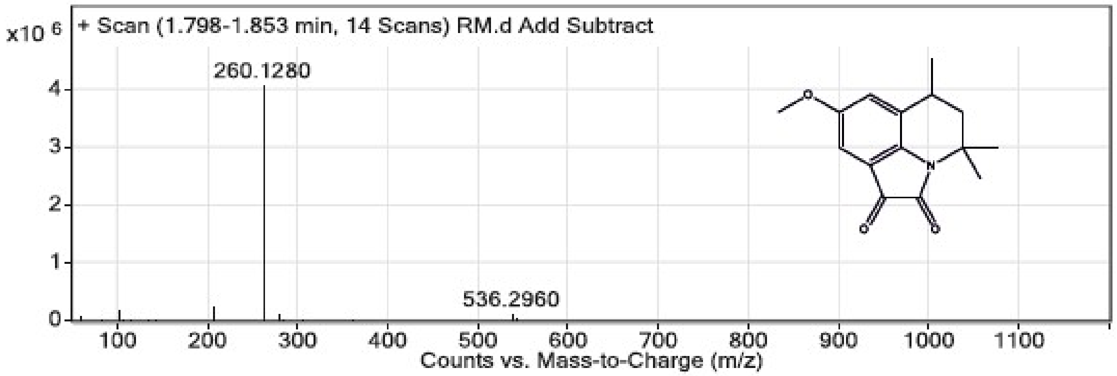

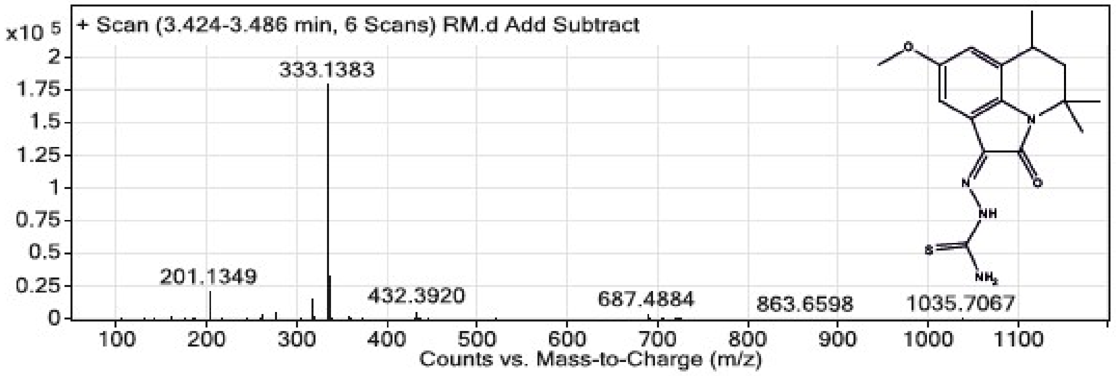

Figure S70. LCMS analysis of the reaction mass after 1 h of interaction of compound 1b with thiosemicarbazide to obtain **2b**
